# Intercepting IRE1 kinase‐FMRP signaling prevents atherosclerosis progression

**DOI:** 10.15252/emmm.202115344

**Published:** 2022-02-22

**Authors:** Zehra Yildirim, Sabyasachi Baboo, Syed M Hamid, Asli E Dogan, Ozlem Tufanli, Sabrina Robichaud, Christina Emerton, Jolene K Diedrich, Hasan Vatandaslar, Fotis Nikolos, Yanghong Gu, Takao Iwawaki, Elizabeth Tarling, Mireille Ouimet, David L Nelson, John R Yates, Peter Walter, Ebru Erbay

**Affiliations:** ^1^ Department of Cardiology Smidt Heart Institute Cedars‐Sinai Medical Center Los Angeles CA USA; ^2^ Department of Molecular Biology and Genetics National Nanotechnology Center Bilkent University Ankara Turkey; ^3^ Department of Molecular Medicine The Scripps Research Institute La Jolla CA USA; ^4^ Lagone Medical Center New York University New York NY USA; ^5^ Department of Biochemistry, Microbiology and Immunology Heart Institute University of Ottawa Ottawa ON Canada; ^6^ Institute of Molecular Health Sciences Swiss Federal Institute of Technology (ETH) Zürich Switzerland; ^7^ Samuel Oschin Cancer Center Cedars‐Sinai Medical Center Los Angeles CA USA; ^8^ Department of Molecular and Human Genetics Baylor College of Medicine Houston TX USA; ^9^ Department of Life Science Medical Research Institute Kanazawa Medical University Ishikawa Japan; ^10^ Department of Biochemistry and Biophysics Howard Hughes Medical Institute University of California at San Francisco San Francisco CA USA; ^11^ David Geffen School of Medicine University of California at Los Angeles Los Angeles CA USA

**Keywords:** atherosclerosis, cholesterol homeostasis, efferocytosis, ER stress, translational regulation, Cardiovascular System, Immunology, Metabolism

## Abstract

Fragile X Mental Retardation protein (FMRP), widely known for its role in hereditary intellectual disability, is an RNA‐binding protein (RBP) that controls translation of select mRNAs. We discovered that endoplasmic reticulum (ER) stress induces phosphorylation of FMRP on a site that is known to enhance translation inhibition of FMRP‐bound mRNAs. We show ER stress‐induced activation of Inositol requiring enzyme‐1 (IRE1), an ER‐resident stress‐sensing kinase/endoribonuclease, leads to FMRP phosphorylation and to suppression of macrophage cholesterol efflux and apoptotic cell clearance (efferocytosis). Conversely, FMRP deficiency and pharmacological inhibition of IRE1 kinase activity enhances cholesterol efflux and efferocytosis, reducing atherosclerosis in mice. Our results provide mechanistic insights into how ER stress‐induced IRE1 kinase activity contributes to macrophage cholesterol homeostasis and suggests IRE1 inhibition as a promising new way to counteract atherosclerosis.

## Graphical abstract

Targeting IRE1 function and substrate(s) provides a novel therapeutic approach to atherosclerosis. We found a key role for IRE1‐mediated FMRP phosphorylation that suppresses the expression of cholesterol transporters and efferocytosis receptors in macrophages and promotes atherosclerosis progression.

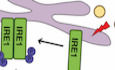

**The paper explained**

**Problem**

A maladaptive immune response to lipid imbalance drives atherosclerosis. Lipid accumulation in plaque‐infiltrating macrophages stresses the ER and promotes atherosclerosis progression. Alleviating ER stress by modulating IRE1 reduces atherosclerosis in a murine model. However, whether IRE1 kinase activity contributes to the atherosclerotic process has remained elusive. Targeting chronic ER stress in atherosclerosis is challenging due to the essential cellular homeostatic functions governed by IRE1. A meticulous exploration of IRE1 kinase function in macrophages and mechanistically authenticated IRE1 kinase substrate(s) can develop into novel therapeutic approaches in atherosclerosis.

**Results**

Here, we identify FMRP as a kinase substrate of IRE1. We show that IRE1 kinase activation leads to FMRP phosphorylation and suppression of macrophage cholesterol efflux and apoptotic cell clearance in a mouse model of atherosclerosis.

**Impact**

Altogether, our findings provide mechanistic insight into the translational regulation of cholesterol efflux and efferocytosis in ER‐stressed macrophages and highlight IRE1 kinase domain and its effector, FMRP, as novel therapeutic targets for atherosclerosis.

## Introduction

Atherosclerosis is a chronic inflammatory disease triggered by imbalanced lipid metabolism (Rocha & Libby, 2009; Weber & Noels, 2011). In atherosclerotic plaques, macrophages ingest lipoproteins and transform into lipid‐laden foam cells. The foamy macrophages lose their ability to migrate away from the plaques, where they sustain a local state of sterile inflammation, which occurs in the absence of pathogens and is typically associated with the release of immune‐recognizable cellular content from damaged or dying cells (Randolph, 2014). Cholesterol efflux and efferocytosis (the engulfment and clearance of apoptotic cells (AC)) by macrophages, on the other hand, help to resolve inflammation and contribute to plaque stability as counterbalancing mechanisms that oppose plaque rupture (Khera *et al*, 2011; Westerterp *et al*, 2013, 2018; Kojima *et al*, 2017; Yurdagul *et al*, 2017). Cholesterol efflux by plaque macrophages is the first step in a multistep process, referred to as “reverse cholesterol transport” (RCT), that reduces lipid accumulation in plaques. Macrophages efflux intracellular cholesterol using their plasma membrane cholesterol transporters (such as the ATP‐binding cassette (ABC) transporters subfamily A member‐1 (ABCA1) and subfamily G member‐1 (ABCG1)), which in turn hand the exported cholesterol over to lipid‐poor apolipoproteins, forming high‐density lipoprotein (HDL) particles (Costet *et al*, 2000; Oram *et al*, 2000). The cholesterol efflux pathway is transcriptionally activated by the metabolic by‐products of AC‐derived cholesterol in the efferocytic macrophages. As such, efferocytosis and RCT synergize to reduce necrosis and resolve inflammation in plaques (Klucken *et al*, 2000; Joyce *et al*, 2002; Tall & Yvan‐Charvet, 2015; Zimmer *et al*, 2016; Guo *et al*, 2018).

Transcriptional and post‐transcriptional regulation play critical roles to set sterile inflammation in motion but also in its resolution and, eventually, plaque regression. Post‐transcriptional regulatory steps often involve RNA‐binding proteins (RBPs) that interact with mRNA to alter its processing, transport, translation, and degradation. Several RBPs have been shown to alter the mRNA stability and translation of cytokines to turn‐off the inflammatory response and key molecular regulators of cholesterol homeostasis and some have been linked to atherosclerosis development (atherogenesis) (Chiu *et al*, 1997; Kang *et al*, 2011; Zhang *et al*, 2013; Ramírez *et al*, 2014; Mobin *et al*, 2016; Haneklaus *et al*, 2017). Fragile X Mental Retardation Protein (FMRP) is an RBP that has been widely studied in neurons due to its causal role in the Fragile X Mental Retardation Syndrome (FXS) (Hersh & Saul, 2011; Bagni *et al*, 2012; Hunter *et al*, 2014). In FXS, a hypermethylated CGG repeat expansion in the 5′ untranslated region (5′ UTR) of the *FMR1* mRNA results in its transcriptional silencing. A subpopulation of the individuals afflicted with FXS and FMRP‐deficient (Fmr1^−/−^) mice have lower cholesterol levels (Leboucher *et al*, 2019b). While a prior study has shown FMRP protein expression is induced in macrophage‐enriched areas of the human atherosclerotic plaque (Hansmeier *et al*, 2018), FMRP’s contribution to the atherosclerotic process has not been investigated directly, and our knowledge of FMRP function has remained largely limited to studies in neurons (Darnell *et al*, 2011). A key serine phosphorylation (S500 in human; S499 in mouse) on FMRP triggers hierarchical phosphorylation of surrounding serines and threonines, while enhancing FMRP’s translation‐repressing activity on many synaptic function‐linked mRNAs bound by it. The identity of the kinase(s) that phosphorylates FMRP has remained subject to intense discussion (Ceman *et al*, 2003; Narayanan *et al*, 2008; Coffee *et al*, 2012; Niere *et al*, 2012; Bartley *et al*, 2014, 2016; Prieto *et al*, 2020).

In this study, we present evidence that ER stress and hypercholesterolemia in mice induces the phosphorylation of macrophage FMRP on S500 by the inositol‐requiring enzyme‐1 (IRE1), a conserved endoplasmic reticulum (ER) stress‐sensing kinase/endoribonuclease (RNase). Metazoans have two IRE1 paralogues, IRE1α (referred to as IRE1 in this paper) and IRE1β. While IRE1α is ubiquitously expressed, IRE1β expression is restricted to gastrointestinal epithelium (Cloots *et al*, 2021). To date, IRE1 has been described to *trans‐*autophosphorylate as a first step in the activation of its RNase modality, which initiates a nonconventional RNA‐splicing reaction and the production of the transcription factor known as spliced X box protein‐1 (XBP1s), which is one of the key drivers of the unfolded protein response (UPR) (Walter & Ron, 2011; Çimen *et al*, 2016a; Robblee *et al*, 2016). IRE1 senses both protein folding stress induced by an accumulation of unfolded proteins in the ER lumen and ER membrane lipid bilayer stress induced by an accumulation of cholesterol or saturated fatty acids (SFA) (Seimon *et al*, 2010; Sukhorukov *et al*, 2020). While previous studies by us and others have shown that ER stress and subsequent IRE1 activation are causally associated with atherosclerosis progression, the mechanism by which IRE1 contributes to the disease pathogenesis has remained elusive (Erbay *et al*, 2009; Zhou & Tabas, 2013; Tufanli *et al*, 2017). Here, we show that IRE1 phosphorylates FMRP on S500, which in turn leads to post‐transcriptional suppression of cholesterol efflux and efferocytosis by macrophages. FMRP deficiency and IRE1 kinase inhibition both enhance RCT and efferocytosis *in vivo*, reducing foam cell formation and atherosclerosis progression in mice. These findings reveal a novel role for FMRP in macrophages in the regulation of cholesterol homeostasis and efferocytosis and provide mechanistic insight into IRE1‐driven atherosclerotic processes during hypercholesterolemia.

## Results

### Lipids induce FMRP phosphorylation in IRE1 kinase‐dependent manner

**Figure 1:**
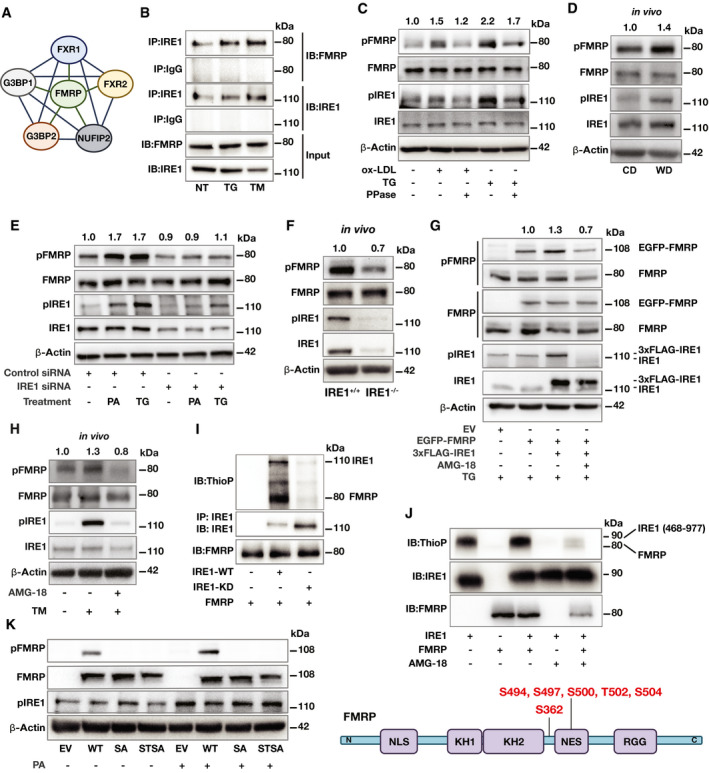
FMRP is a novel IRE1 kinase substrate STING analysis of published IRE1 interactome proteins in relation to FMRP (Acosta‐Alvear *et al*, 2018).HEK293T cells were co‐transfected with IRE1 and FMRP plasmids and stimulated with TG (600 nM) or TM (1 mg/ml) for 2 h. Protein lysates were immunoprecipitated (IP) with anti‐IRE1 or IgG (control) antibodies and analyzed by Western blotting using specific antibodies for FMRP and IRE1 (*n* = 3 biological replicates).RAW 264.7 mouse macrophages were treated with either oxLDL (50 µg/ml) or TG (300 nM) for 6 h. Protein lysates were treated with λ Phosphatase (PPase) for 30 min and analyzed by Western blotting using specific antibodies for pFMRP, FMRP, pIRE1, IRE1, and β‐Actin. pFMRP/FMRP fold induction is depicted above the blots (*n* = 6 biological replicates).Apoe^−/−^ mice were fed with chow diet (CD) or western diet (WD) for 16 weeks followed by peritoneal macrophage (PM) isolation. Protein lysates were analyzed by Western blotting using specific antibodies for pFMRP, FMRP, pIRE1, IRE1, and β‐Actin. pFMRP/FMRP‐fold induction is depicted above the blots (*n* = 5 mice per group).Control‐ or IRE1‐siRNA transfected HEK293T cells were stimulated by either PA (500 µM) or TG (600 nM) for 4 h. Protein lysates were analyzed by Western blotting using specific antibodies for pFMRP, FMRP, pIRE1, IRE1, and β‐Actin. pFMRP/FMRP fold induction is depicted above the blots (*n* = 4 biological replicates).Protein lysates of thioglycolate‐elicited PM from IRE1α^+/+^ and IRE1α^−/−^ mice (after 16 weeks on WD) were analyzed by Western blotting using specific antibodies for pFMRP, FMRP, pIRE1, IRE1, and β‐Actin. pFMRP/FMRP fold induction is depicted above the blots (*n* = 4 mice per group).MEF cells were transfected with either empty vector, EGFP‐FMRP or 3xFLAG‐IRE1 plasmids then pre‐treated either with vehicle (dimethyl sulfoxide, DMSO) or AMG‐18 (25 µM; 1 h) followed by TG (600 nM) stimulation for 4 h. Protein lysates were analyzed by Western blotting using specific antibodies for pFMRP, FMRP, pIRE1, IRE1, and β‐Actin. pFMRP/FMRP fold induction is depicted above the blots (*n* = 4 biological replicates).C57BL/6 were injected either with DMSO or AMG‐18 (30 mg/kg; 8 h), followed by TM injection (1 mg/kg; 8 h). Protein lysates of thioglycolate‐elicited PM were analyzed by Western blotting using antibodies for pFMRP, FMRP, pIRE1, IRE1, and β‐Actin. pFMRP/FMRP fold induction is depicted above the blots (*n* = 4 mice per group).HEK293T cells were transfected with either empty vector (EV), IRE1‐WT, or IRE1–KD plasmids and stimulated by TG (600 nM; 1 h). Protein lysates from each transfection were separately immunoprecipitated (IP) with anti‐IRE1 antibody and subjected to a kinase reaction with purified hFMRP protein and ATP‐γ‐S (100 µM) in kinase buffer. The IP protein were analyzed by Western blotting using specific antibodies for thiophosphate esters (ThioP), IRE1, and FMRP (*n* = 3 biological replicates).Purified FMRP and IRE1 kinase (activated) proteins were subjected to kinase assay and analyzed by Western blotting using specific antibodies for ThioP, IRE1, and FMRP (*n* = 3 biological replicates) and with LC‐MS/MS. Identified IRE1 kinase‐mediated FMRP phosphorylation sites (bottom).Fmr1^−/−^ mouse embryonic fibroblasts (MEF) were transfected either with EV, WT‐FMRP, SA‐FMRP, or STSA‐FMRP plasmids followed by PA treatment (500 µM; 6 h). Protein lysates were analyzed by Western blotting using specific antibodies for FMRP, pFMRP, pIRE1, and β‐Actin (*n* = 3 biological replicates). Data information: A representative blot is shown. In D, E, G, and H data are cumulative results of two independent experiments. Data are mean ± SEM. Unpaired *t‐*test with Welch’s correction or paired *t‐*test. Source data are available online for this figure.

Previously published mass spectrometry‐based IRE1 interactome data revealed multiple FMRP‐interacting proteins potentially associating with IRE1 (Fig 1A) (Acosta‐Alvear *et al*, 2018). These proteins share significant sequence homology with FMRP and are usually found in homo/heteromeric complexes with FMRP (Zhang *et al*, 1995). Based on these observations, we reasoned that FMRP might also physically interact with IRE1. Indeed, FMRP co‐immunoprecipitated with IRE1 in non‐stress and ER stress conditions induced by thapsigargin (TG; an inhibitor of the ER Ca^2+^ pump) and tunicamycin (TM; an inhibitor of N‐linked glycosylation) from human embryonic kidney cell line (HEK293T) cells that were transiently transfected with plasmids encoding both proteins (Fig 1B).

Since FMRP phosphorylation is critical for translation suppression and the association with IRE1 juxtaposes it to a kinase whose substrate is unclear, we wondered whether ER stress alters FMRP phosphorylation state. To assess this possibility, we treated cultured macrophages with known ER stressors, such as TG and oxidized low‐density lipoprotein (oxLDL, another inducer of ER stress). Using specific antibodies that recognize S724 phosphorylation on IRE1 and S500 phosphorylation on FMRP (Reynolds *et al*, 2015; Wang *et al*, 2021), we found that TG and oxLDL significantly induced IRE1 autophosphorylation and FMRP phosphorylation but did not affect levels of FMRP protein or Fmr1 mRNA (Fig 1C and Appendix Fig S1A and B). Phosphatase treatment of the samples partially reversed the ER stress‐induced increase in the pFMRP/FMRP ratio (Fig 1C, Appendix Fig S1B), implying FMRP phosphorylation is enhanced by these ER stressors.

Previous studies showed that hyperlipidemia induces ER stress in plaque macrophages *in vivo* (Moore *et al*, 2013; Kim *et al*, 2016). We next investigated whether hyperlipidemia also has an effect on FMRP phosphorylation on S499 (mouse S499 corresponds to human S500). To this end, we used mice deficient in apolipoprotein E (Apoe^−/−^) as this is a protein found in plasma lipoprotein particles and facilitates cholesterol clearance from the circulation (Davignon *et al*, 1999). In agreement with previous reports, we observed that ER stress (as monitored by IRE1 autophosphorylation) was induced in the peritoneal macrophages (PM) obtained from hyperlipidemic, Apoe^−/−^ mice that were fed with a Western diet (WD) for 16 weeks when compared to Apoe^−/−^ mice fed with chow diet (CD) (Fig 1D). We found that FMRP S499 phosphorylation, which leads to FMRP‐mediated translational suppression, was 1.4‐fold elevated by a chronic exposure to hypercholesterolemia, whereas FMRP protein and Fmr1 mRNA expression levels remained unchanged (Fig 1D; Appendix Fig S1C and D).

We next asked what role IRE1 plays in ER stress‐induced FMRP phosphorylation. To address this question, we transfected IRE1‐specific siRNA to suppress IRE1 expression in a human cell line, followed by ER stress induction by a saturated fatty acid, palmitate (PA), that is known to induce ER stress, or TG. While both ER stressors induced the phosphorylation of IRE1 and FMRP, this was prevented in IRE1 knock‐down cells (Fig 1E and Appendix Fig S1E). In addition, we analyzed hypercholesterolemia‐induced FMRP phosphorylation in PMs obtained from Apoe^−/−^ mice with a genetic deletion of IRE1α in the myeloid lineage (IRE1^−/−^) (see Methods) after feeding with WD for 16 weeks. FMRP phosphorylation, but not FMRP protein or Fmr1 mRNA, was reduced in PM isolated from IRE1^−/−^ mice when compared to those isolated from IRE1^+/+^ mice (Fig 1F, Appendix Fig S1F and G). While these results show a clear reduction in FMRP phosphorylation upon siRNA treatment or gene knock‐out, we observed residual signal in both cases (Fig 1E and F). As we confirm below (Fig 1K), the antibody used in these experiments is phosphorylation specific. We, therefore, surmise that partial phosphorylation on S500/S499 may also be mediated by the other kinases that are known to phosphorylate this residue on FMRP (Narayanan *et al*, 2008; Bartley *et al*, 2016), in addition to IRE1 as we show here. Collectively, our *in vitro* and *in vivo* results strongly support that ER stress‐induced activation of IRE1 kinase leads to enhanced phosphorylation of FMRP on S500/S499.

### IRE1 phosphorylates FMRP

To begin investigating the role of IRE1’s kinase activity in ER stress‐induced FMRP phosphorylation, we expressed a 3xFLAG‐tagged IRE1 (FLAG‐IRE1) and/or EGFP‐FMRP in wild‐type mouse embryonic fibroblasts (MEFs). Endogenous FMRP and IRE1 migrated faster in SDS–PAGE gel than the epitope tagged EGFP‐FMRP and FLAG‐IRE1, respectively. In all conditions, the MEFs were TG‐treated (to stimulate IRE1 kinase activity) in the absence or presence of an IRE1 kinase‐specific inhibitor (AMG‐18) (Papandreou *et al*, 2011; Ghosh *et al*, 2014; Tufanli *et al*, 2017; Harnoss *et al*, 2019). In the absence of AMG‐18, both IRE1 and FMRP were phosphorylated. AMG‐18 treatment prevented IRE1 phosphorylation while clearly reducing FMRP phosphorylation (Fig 1G and Appendix Fig S1H). While these data further support the notion that IRE1 phosphorylates FMRP during ER stress, alternative kinase(s) appears to mediate the same reaction. This is consistent the published data that several kinases can phosphorylate the same residue on FMRP (Narayanan *et al*, 2008; Bartley *et al*, 2016). Furthermore, in an *in vivo* setting where mice were injected with TM to induce IRE1 kinase activity, treatment with AMG‐18 inhibited IRE1 kinase activity and reduced FMRP phosphorylation in PM (Fig 1H and Appendix Fig S1I). Taken together, our findings support the notion that IRE1 kinase activity makes an important contribution to ER stress‐induced FMRP S499 phosphorylation in mouse macrophages and MEFs as well as S500 in HEK293T cells. Our data also support that other known or unknown FMRP kinase(s) are responsible for basal FMRP phosphorylation that is observed in non‐stress conditions.

To ask whether IRE1 can phosphorylate FMRP directly, we employed *in vitro* assays. To this end, we immunoprecipitated wild type (WT) or kinase dead (KD) mutant IRE1 from HEK293T cells (after treating with ER stressor) and incubated the immunoprecipitates with purified, recombinant human FMRP protein in a kinase reaction. The reaction included ATP‐γ‐S instead of ATP, which allows kinases to thio‐phosphorylate their substrates. The resultant kinase reaction was analyzed by Western blotting using an anti‐thiophosphate ester antibody. In the reaction containing IRE1‐WT, both proteins were thio‐phosphorylated, but not in the reaction containing the IRE1‐KD mutant (Fig 1I).

To determine the specific amino acids phosphorylated by IRE1, we performed the kinase reaction using purified, recombinant human IRE1‐kinase/RNase domains (amino acids 468–977) and human FMRP proteins. Both IRE1 and FMRP were phosphorylated in this reaction in an AMG‐18‐sensitive manner (Fig 1J). Liquid chromatography‐mass spectrometry (LC‐MS/MS)‐based analysis of the phosphorylated FMRP residues in this kinase reaction revealed eight IRE1‐induced phosphorylation sites on FMRP at serine (S362, S494, S497, S500, S504) and threonine (T502, T592, T594) (Fig 1J, Appendix Fig S1J and K). Importantly, S500 in human FMRP is the previously identified FMRP phosphorylation site that was shown to enhance FMRP‐mediated translational suppression (Ceman *et al*, 2003; Narayanan *et al*, 2008; Coffee *et al*, 2012; Niere *et al*, 2012; Bartley *et al*, 2014, 2016; Prieto *et al*, 2020).

Using site‐directed mutagenesis, we engineered two mutant versions of FMRP, in which we either changed S500 to alanine (SA mutant) or S500, T502, and S504 to alanine (STSA triple mutant) to block phosphorylation at S500 and amino acids in its proximity. We then induced ER stress with PA in Fmr1^−/−^ MEFs transiently transfected with WT FMRP and FMRP mutants (SA and STSA). PA induced FMRP phosphorylation in WT FMRP‐reconstituted cells but failed to do so in cells expressing the SA and STSA mutants of FMRP (Fig 1J). These data confirm the specificity of the FMRP antibody for phosphorylated S500 and is consistent with the notion that IRE1 kinase directly phosphorylates human FMRP protein on S500.

### IRE1‐FMRP signaling induces foam cell formation while suppressing RCT

**Figure 2:**
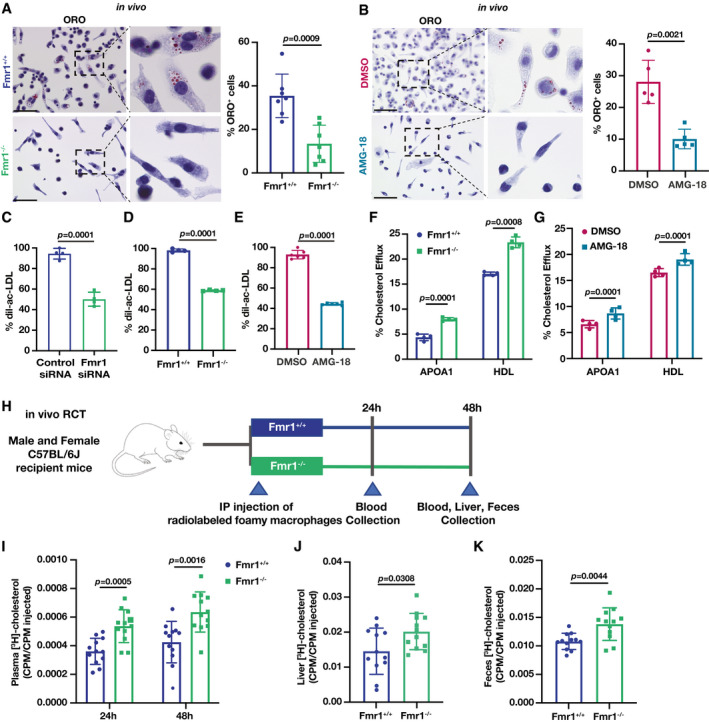
FMRP deficiency enhances RCT while reducing foam cell formation *in vivo* AFmr1^+/+^ and Fmr1^−/−^ mice were injected with AAV_PCSK9 and fed with 16 weeks of WD. Residential PM were stained with Oil Red O (ORO) and imaged (*n* = 7 mice per group; Scale bar = 50 µm).BApoe^−/−^ mice were fed with WD (12 weeks) and injected with vehicle (DMSO) or AMG‐18 (30 mg/kg/day) in the last 4 weeks of WD. Residential PM were stained with ORO and imaged (*n* = 5 mice per group; Scale bar = 50 µm).C–EFlow cytometry analysis of BMDMs after dil‐ac‐LDL (25 µg/ml) loading for 24 h; (C) control‐ or Fmr1‐siRNA transfected BMDM (*n* = 4 biological replicates), (D) Fmr1^+/+^ and Fmr1^−/−^ BMDM (*n* = 4 biological replicates), (E) BMDM pre‐treated with either vehicle (DMSO) or AMG‐18 (5 µM; 1 h) (*n* = 6 biological replicates).F, GMacrophages were pre‐loaded with fluorescently labeled cholesterol (16 h) followed by incubation in efflux medium including APOA1 (25 µg/ml) or HDL (50 µg/ml) as acceptors for 6 h. % Efflux was calculated as cholesterol signal in medium/cholesterol signal in medium and cell: Cholesterol efflux in (F) Fmr1^+/+^ and Fmr1^−/−^ BMDM (*n* = 4 biological replicates) and in (G) BMDM that were pre‐treated either with DMSO or AMG‐18 (5 µM; 1 h) (*n* = 4 biological replicates).H–KRCT experiment: (H) Schematic representation of C57BL6 mice were injected with [^3^H]‐cholesterol‐loaded foamy Fmr1^+/+^ and Fmr1^−/−^ BMDM, (I) plasma cholesterol levels after 24 and 48 h, (J) liver cholesterol levels after 48 h, and (K) feces cholesterol levels after 48 h (*n* = 12 mice per group). Data information: Data are mean ± SEM. Unpaired *t‐*test with Welch’s correction. Source data are available online for this figure.

We next wondered what role FMRP plays in macrophage biology that is relevant to atherosclerotic plaque development. We performed an *in vivo* macrophage foam cell formation assay in the peritoneum of Fmr1^−/−^ and Fmr1^+/+^ mice, using a well‐established method in which we induced hyperlipidemia using a combination of adenoviral‐delivery of proprotein convertase subtilisin kexin 9 (AAV_PCSK9), a protein that directs hepatic low‐density lipoprotein (LDL) receptors for degradation, and feeding with WD for 16 weeks (Li *et al*, 2007; Tsimikas *et al*, 2011; Peled *et al*, 2017). FMRP deficiency significantly reduced foam cell formation *in vivo* (Fig 2A). Next, we fed Apoe^−/−^ mice with a WD (12 weeks) and injected them daily with the IRE1 kinase inhibitor, AMG‐18, or vehicle for the last 4 weeks. AMG‐18 reduced foam cell formation in the peritoneum *in vivo* (Fig 2B). This result indicates that IRE1‐FMRP signaling axis enhances foam cell formation.

We also transfected wild type BMDMs with either Fmr1‐specific or control siRNA and followed by loading the cells with 3,3′‐dioctadecylindocarbocyanine (dil)‐labeled acetylated LDL (Ac‐LDL) (Carotti *et al*, 2021). Flow cytometry analysis revealed that Fmr1 silencing significantly reduced % dil‐acLDL internalized in macrophages (Fig 2C, Appendix Fig S2A). Likewise, Fmr1^−/−^ BMDM displayed reduced % dil‐acLDL internalization when compared to Fmr1^+/+^ BMDM (Fig 2D). A similar reduction in foam cell formation was observed with the IRE1 kinase inhibitor (Fig 2E). These results indicate that both the inhibition of IRE1 kinase activity and the genetic deletion of its proposed substrate, FMRP, reduce foam cell formation *in vitro* and *in vivo*. Reduced foam cell formation could be explained with less cholesterol uptake, however, neither FMRP knock down nor IRE1 kinase inhibition altered cholesterol uptake in macrophages (Appendix Fig S2B). We reasoned that this observation is most likely related to an increase in cholesterol export (RCT; due to increased translation of cholesterol exporters) from Fmr1^−/−^ macrophages. Indeed, FMRP deficiency led to an increase in cholesterol efflux coupled with its loading onto the cholesterol carriers, apolipoprotein‐A1 (APOA1) and HDL (Fig 2F), and, likewise, the IRE1 kinase inhibitor enhanced cholesterol efflux (Fig 2G). Thus, ER stress‐associated reduction in cholesterol efflux is dependent on both IRE1 kinase activity and FMRP.

Next, we determined whether the absence of FMRP in BMDMs also enhances RCT *in vivo*. To this end, we pre‐loaded Fmr1^+/+^ and Fmr1^−/−^ BMDMs with [^3^H]‐cholesterol and injected the cells subcutaneously into WT mice (Fig 2H). FMRP deficiency in macrophages significantly increased radioactivity counts ([^3^H]‐cholesterol) in the plasma, liver, and feces of the recipient mice, when compared to recipient mice that received Fmr1^+/+^ macrophages (Fig 2I–K), demonstrating that FMRP deficiency in macrophages enhances RCT in mice.

### FMRP regulates macrophage efferocytosis

**Figure 3:**
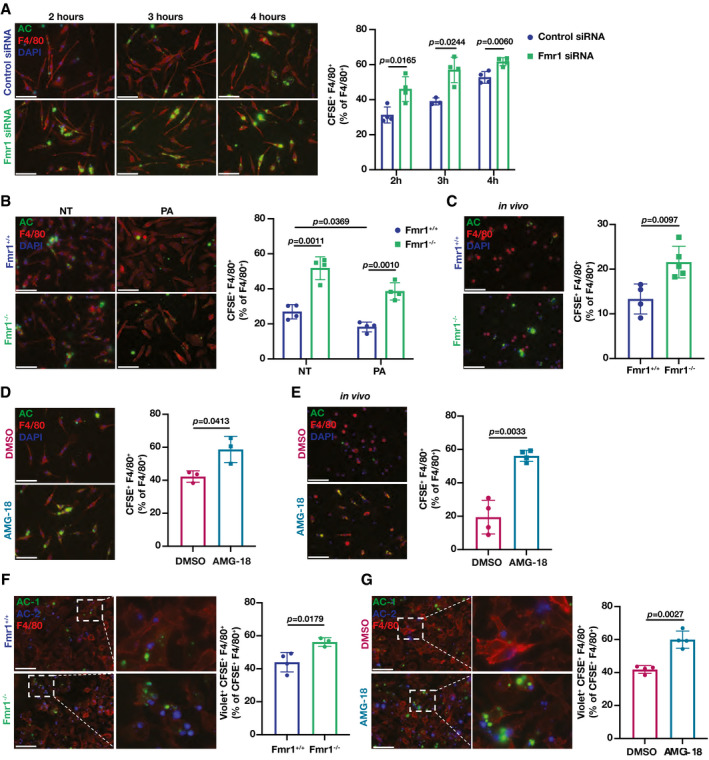
FMRP deficiency increases efferocytosis *in vivo* A–E
*In vitro* and *in vivo* efferocytosis experiments, where percentage of macrophages F4/80^+^ (red) that ingested apoptotic cells (AC) labeled with carboxyfluorescein succinimidyl ester (CFSE)^+^ (green) were reported as % efferocytosis. (A) BMDMs were transfected with Fmr1‐ or control‐siRNA and incubated CFSE‐labeled AC for the indicated hours (*n* = 4 biological replicates). (B) Fmr1^+/+^ and Fmr1^−/−^ BMDMs were treated with PA (500 µM) for 6 h and then incubated with CFSE‐labeled ACs for 4 h (*n* = 4 biological replicates). (C) Fmr1^+/+^ and Fmr1^−/−^ mice were fed WD (16 weeks) and injected intraperitoneally with CFSE‐labeled AC (1.5 h), followed by PM elicitation (*n* = 4–5 mice per group). (D) BMDM were pre‐treated either with vehicle (DMSO) or AMG‐18 (5 µM) for 1 h then incubated with CFSE‐labeled Acs for 4 h (*n* = 3 biological replicates). (E) C57BL/6 mice were injected with AMG‐18 (30 mg/kg) or vehicle (DMSO) for 8 h, followed by intraperitoneal injection with CFSE‐labeled ACs for 1.5 h and PM elicitation (*n* = 4 mice per group).F, G
*In vitro* continuous *efferocytosis experiments*, where macrophages were stained for F4/80^+^ (red), AC were labeled with CFSE (AC‐1; green) or Violet (AC2; violet). % continuous efferocytosis was determined by the ratio of F4/80^+^, CFSE^+^, and Violet^+^ (triple positive) cells to total F4/80^+^ and CFSE^+^ (double positive) cells. (F) Fmr1^+/+^ and Fmr1^−/−^ BMDM were incubated with AC‐1 for 2 h, and after 2 h interval, incubated with AC‐2 for 2 more hours (*n* = 4–3 biological replicates). (G) BMDM were pre‐treated either with vehicle (DMSO) or AMG‐18 (5 µM) for 1 h, incubated with CFSE‐labeled AC‐1 for 2 h, followed by incubation with Violet‐labeled AC‐2 for 2 h and PM collection (*n* = 4 biological replicates). Data information: For all images scale bar = 50 µm; Red: Macrophages, Green: AC/AC‐1, Violet: AC‐2. Data are mean ± SEM. Unpaired *t‐*test with Welch’s correction. Source data are available online for this figure.

We next investigated the impact of FMRP deficiency on efferocytosis, a primary process that promotes atherosclerotic plaque regression by removing apoptotic macrophages in the lesion area. To this end, we transfected BMDM (red fluorescent stained) with Fmr1‐specific or control siRNA and incubated them with green fluorescent‐labeled apoptotic cells (ACs), in which apoptosis was induced by ultraviolet (UV) irradiation. FMRP knock‐down increased efferocytosis of ACs (as measured by colocalization of the fluorescent markers), when compared to control (Fig 3A). FMRP‐deficient BMDMs also increased efferocytosis when compared to wild type BMDMs under both no‐stress and PA‐induced ER stress conditions (Fig 3B). Next, we induced hyperlipidemia in Fmr1^−/−^ and Fmr1^+/+^ mice as described above using a combination of AAV_PCSK9 injection and feeding with a WD. We then injected the mice intraperitoneally with green fluorescent‐labeled ACs and harvested PM macrophages. FMRP‐deficient PMs displayed enhanced efferocytosis compared to WT PMs (Fig 3C).

We next asked how IRE1 kinase activity impacts efferocytosis by macrophages. We observed that AMG‐18 increased efferocytosis of ACs in BMDMs (Fig 3D). Next, we injected wild type mice with AMG‐18 (for 8 h) followed by injection of green fluorescent‐labeled ACs. AMG‐18 also induced efferocytosis of ACs by PM *in vivo* (Fig 3E).

Continued clearance of ACs by macrophages prevents the accumulation of necrotic cells and is an important process that promotes atherosclerosis regression (Yurdagul *et al*, 2017). To determine whether Fmr1^−/−^ macrophages can efficiently internalize multiple ACs over consecutive rounds of engulfment, we incubated macrophages with green fluorescent‐labeled AC for 2 h, followed by second incubation with violet fluorescent‐labeled AC for 2 more hours. FMRP‐deficient BMDMs displayed an increase in continued efferocytosis when compared to wild type BMDMs (Fig 3F). Likewise, treatment of wild type BMDM with AMG‐18 enhanced continued efferocytosis of ACs (Fig 3G). Collectively, these results demonstrate that the ablation of IRE1 kinase activity and its proposed substrate, FMRP, enhances efferocytosis *in vitro* and *in vivo*.

### Translational suppression of cholesterol transporters and efferocytosis regulators by FMRP during ER stress

**Figure 4:**
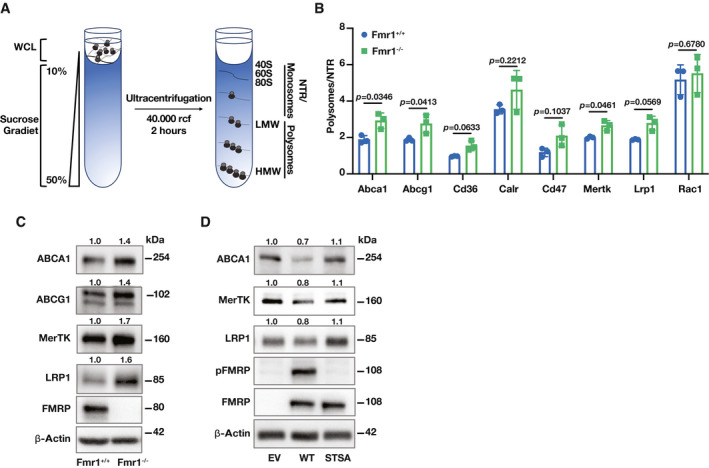
FMRP targets in macrophages A, BRNA lysates from Fmr1^+/+^ and Fmr1^−/−^ BMDM that were treated with PA (500 µM; 6 h) were fractionated using a 10–50% sucrose gradient and separated to polysome, monosome/NTR fractions. The absorbance (260 nm) of RNA was measured and plotted as a function of time (*n* = 3 biological replicates). (A) Representative profile for RNA distribution from genotypes based on UV absorbance readings after sucrose gradient fractionation. (B) The ratio of the Abca1, Abcg1, Mertk, Lrp1, Cd36, Cd47, and Rac1 mRNA in polysome to NTR fraction (*n* = 3 biological replicates).CBMDM were isolated from Fmr1^+/+^ and Fmr1^−/−^, and protein lysates were analyzed by Western blotting using specific antibodies for ABCA1, ABCG1, MerTK, LRP1, FMRP, and β‐Actin antibodies and fold inductions relative to β‐Actin are depicted above the blots (*n* = 6 biological replicates).DFmr1^−/−^ MEF cells were transfected with EV, WT‐FMRP, or STSA‐FMRP plasmids followed by PA treatment (500 µM; 6 h). Protein lysates were analyzed by Western blotting using specific antibodies for ABCA1, MerTK, LRP1, pFMRP, FMRP, and β‐Actin and fold inductions relative to β‐Actin are depicted above the blots (*n* = 5 biological replicates). Data information: A representative blot is shown. In C and D, data are cumulative results of 2 and 3 independent experiments, respectively. In Western blots, the protein expression fold change was calculated relative to β‐Actin and depicted above the blots and a representative blot was shown. Data are mean ± SEM. Unpaired *t‐*test with Welch’s correction. Source data are available online for this figure.

Consistent with our observations that macrophage FMRP plays a role in suppressing cholesterol efflux and efferocytosis, published data describing the FMRP‐RNA interactome also suggest that FMRP interacts with mRNA‐encoding proteins involved in cholesterol trafficking (such as Abca1 and Abcg1) and efferocytosis receptors (such as c‐Mer tyrosine kinase (Mertk) and LDL receptor‐related protein 1 (Lrp1)), suggesting FMRP may impair their translation (Darnell *et al*, 2011; Ascano *et al*, 2012). To assess this notion in macrophages, we next performed polyribosome profiling in Fmr1^+/+^ and Fmr1^−/−^ BMDMs under PA‐induced ER stress conditions (Fig 4A and B, Appendix Fig S3A). Indeed, the mRNA abundance for the cholesterol transporters, Abca1 and Abcg1, and the mRNA abundance for efferocytosis regulators, Mertk, Lrp1, and Cd36, were increased in translating polysome fractions and decreased in non‐translating (NTR) fractions in the Fmr1^−/−^ BMDMs when compared to Fmr1^+/+^ BMDMs (Fig 4B), while the abundance of these mRNAs in the total cell lysate was unchanged (Appendix Fig S3B).

We next analyzed the corresponding protein expression changes for the FMRP‐regulated mRNA targets from the same experiment in Fig 4A and B. As expected, ABCA1, ABCG1, MerTK, and LRP1 protein expression levels were induced in Fmr1^−/−^ BMDMs (Fig 4C and Appendix Fig S3C). To further assess the impact of IRE1‐mediated FMRP phosphorylation on the translation of FMRP’s targets, we overexpressed the FMRP phosphorylation‐deficient mutant (STSA) or WT‐FMRP in Fmr1^−/−^ MEFs and treated with PA to induce ER stress. ABCA1, LRP1, and MerTK expression levels were reduced in the Fmr1^−/−^ MEF expressing WT‐FMRP, but not in Fmr1^−/−^ MEFs expressing STSA‐FMRP (Fig 4D and Appendix Fig S3D). Taken together, our data demonstrate that IRE1‐mediated FMRP phosphorylation tunes cholesterol transporters and efferocytosis regulators expression in macrophages.

### FMRP knock‐down and IRE1 kinase inhibition alleviates atherosclerosis

**Figure 5:**
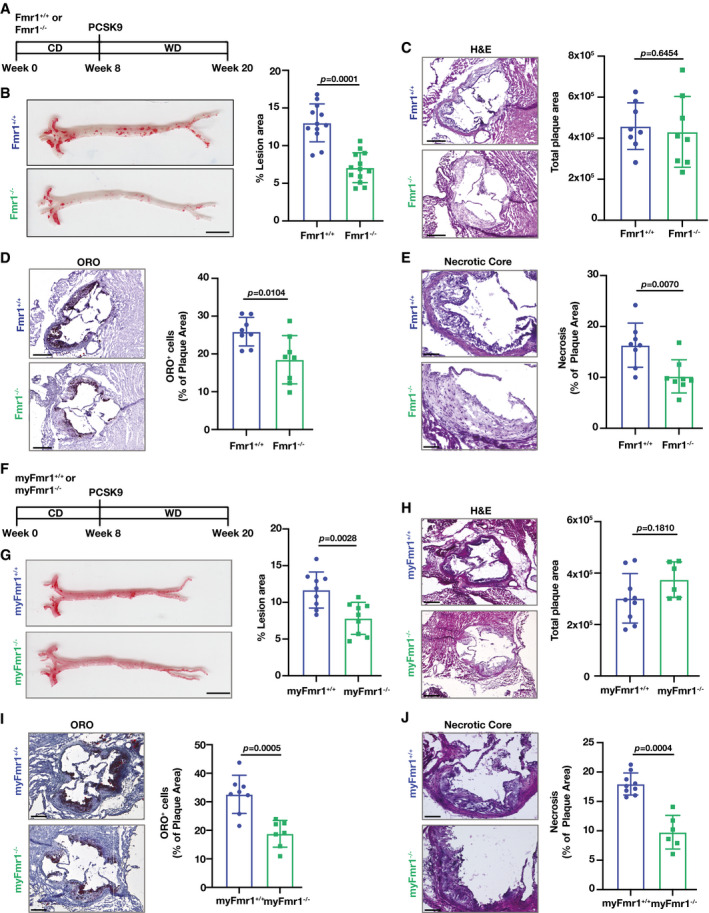
FMRP‐deficiency alleviates atherosclerosis Atherosclerosis experiment design in Fmr1^+/+^ and Fmr1^−/−^ mice that were injected with AAV_PCSK9 and fed WD (16 weeks).Lesion area calculated from *en face* aorta, stained with ORO (*n* = 12–13 mice per group; Scale bar = 5 mm).Total plaque area was calculated from hematoxylin & eosin (H&E)‐stained aortic root sections (*n* = 8 mice per group; Scale bar = 300 µm).Foam cell area was calculated from ORO‐stained aortic root sections (*n* = 8 mice per group; Scale bar = 300 µm).Necrotic area was calculated from H&E‐stained aortic root sections (*n* = 8 mice per group; Scale bar = 100 µm).Atherosclerosis experiment design in myFmr1^+/+^ and myFmr1^−/−^ mice that were injected with AAV_PCSK9 and fed WD (16 weeks).Lesion area calculated from *en face* aorta, stained with ORO (*n* = 9 mice per group; Scale bar = 5 mm).Total plaque area was calculated from H&E‐stained aortic root sections (*n* = 9–6 mice per group; Scale bar = 300 µm).Foam cell area was calculated from ORO‐stained aortic root sections (*n* = 9–6 mice per group; Scale bar = 300 µm).Necrotic area was calculated from H&E‐stained aortic root sections (*n* = 9–6 mice per group; Scale bar = 100 µm). Data information: Data are mean ± SEM; Mann Whitney *U* test. Source data are available online for this figure.

Our findings demonstrate that both IRE1 kinase inhibition and FMRP deficiency result in increased RCT, reduced foam cell formation, and enhanced efferocytosis *in vivo*, suggesting that FMRP deficiency in mice leads to protection from atherosclerosis. We tested this notion using Fmr1^−/−^ and Fmr1^+/+^ mice in which hyperlipidemia was induced by combining AAV_PCSK9 injection with a WD as described above (Fig 5A). Although there was a very slight decrease in body weight ratio; the plasma glucose, total plasma cholesterol (TPC), lipoprotein levels, and the number of circulating, major type of immune cells were indistinguishable between the Fmr1^−/−^ and Fmr1^+/+^ genotypes (Appendix Fig S4A–F). Yet, FMRP deficiency resulted in a significant reduction in atherosclerotic lesions in *en face* aorta preparations (Fig 5B). FMRP deficiency did not alter aortic root lesion area despite a significantly decreased foam cell area (as assessed by Oil Red O staining) (Fig 5C and D). We next asked what may be contributing to this phenotype. The necrotic core area in the lesions from Fmr1^−/−^ mice was significantly less than in Fmr1^+/+^ lesions (Fig 5E), indicating improved AC clearance by Fmr1^−/−^ macrophages in plaques. We also observed a significant reduction in lesion macrophages (as assessed by the anti‐monocyte macrophage 2 (MOMA‐2)‐stained area) and the number of apoptotic cells (TUNEL‐stained) per macrophage area (Appendix Fig S4G and H). We further investigated whether apoptosis is altered in Fmr1^−/−^ and AMG‐18‐treated macrophages. There was no significant change between the groups (Appendix Fig S4I), supporting the notion that the primary consequence of inhibiting IRE1‐FMRP signaling is efficient clearance of apoptotic cells through increasing efferocytosis capacity. Additionally, we detected no change in smooth muscle area (stained with smooth muscle actin (SMA)), but there was a significant increase in the collagen content of Fmr1^−/−^ lesions when compared to Fmr1^+/+^ lesions, suggesting this could be the reason why aortic root lesion area is not significantly altered (Appendix Fig S4J and K).

To approach the role of macrophage FMRP in atherosclerosis, we generated a myeloid Fmr1‐deficient mouse model (myFmr1^−/−^) and induced hyperlipidemia (Fig 5F). As expected from the systemic deletion data shown in Fig 5A, the body weight, plasma glucose and TPC were indistinguishable between the myFmr1^−/−^ and myFmr1^+/+^ genotypes (Appendix Fig S4L–N). in general, myeloid‐specific FMRP deficiency paralleled the results obtained for Fmr1^−/−^ mice: it resulted in a significant reduction in atherosclerotic lesions in *en face* aorta preparations (Fig 5G). Myeloid‐specific FMRP deficiency did not alter aortic root lesion area but significantly reduced foam cell area (Fig 5H and I). The necrotic core area in the lesions from myFmr1^−/−^ mice was also significantly less than in myFmr1^+/+^ lesions (Fig 5J), indicating improved AC clearance by Fmr1^−/−^ macrophages in plaques and supporting our observations that FMRP deficiency increases macrophage efferocytosis *in vitro* and *in vivo* (Fig 3). Thus, comparing the data obtained with the Fmr1^−/−^ mice with those obtained from the myFmr1^−/−^ mice indicates that the atheroprotective effects observed in Fmr1^−/−^ mice are mostly, if not exclusively, due to FMRP’s role in myeloid cells such as macrophages.

**Figure 6:**
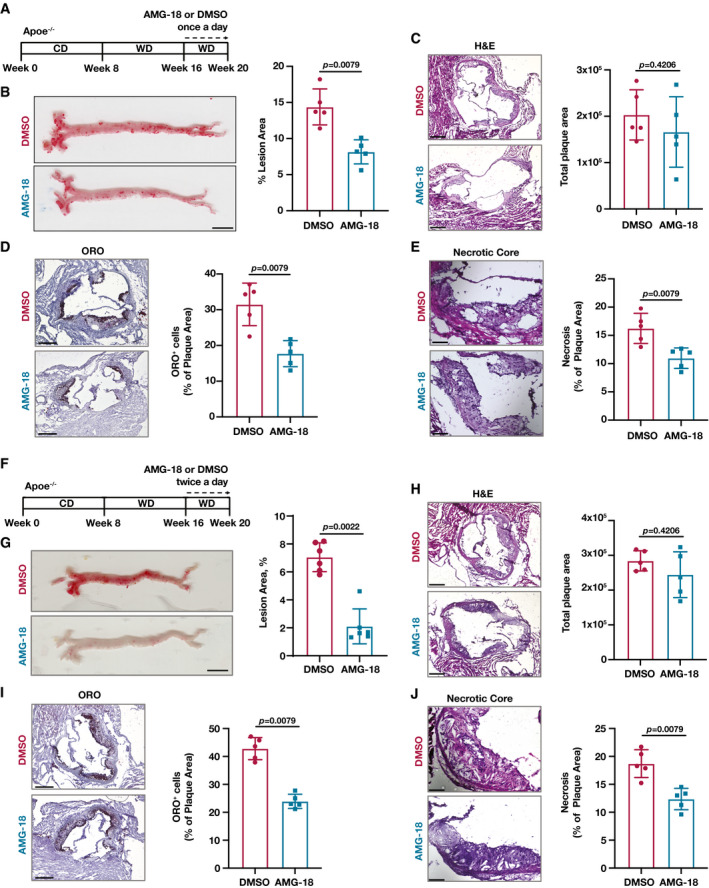
IRE1 Kinase inhibition alleviates atherosclerosis Atherosclerosis experiment design in Apoe^−/−^ mice were fed with WD (12 weeks) and injected with vehicle (DMSO) or AMG‐18 (30 mg/kg) once a day in the last 4 weeks of WD.Lesion area calculated from *en face* aorta, stained with ORO (*n* = 5 mice per group; Scale bar = 5 mm).Total plaque area was calculated from H&E‐stained aortic root sections (*n* = 5 mice per group; Scale bar = 300 µm).Foam cell area was calculated from ORO‐stained aortic root sections (*n* = 5 mice per group; Scale bar = 300 µm).Necrotic area was calculated from H&E‐stained aortic root sections (*n* = 5 mice per group; Scale bar = 100 µm).Atherosclerosis experiment design in Apoe^−/−^ mice were fed with WD (12 weeks) and injected with vehicle (DMSO) or AMG‐18 (30 mg/kg) twice a day in the last 4 weeks of WD.Lesion area calculated from *en face* aorta, stained with ORO (*n* = 6 mice per group; Scale bar = 5 mm).Total plaque area was calculated from H&E‐stained aortic root sections (*n* = 5 mice per group; Scale bar = 300 µm).Foam cell area was calculated from ORO‐stained aortic root sections (*n* = 5 mice per group; Scale bar = 300 µm).Necrotic area was calculated from H&E‐stained aortic root sections (*n* = 5 mice per group; Scale bar = 100 µm). Data information: Data are mean ± SEM; Mann Whitney *U* test. Source data are available online for this figure.

To test the notion that IRE1 functions upstream of FMRP, we next investigated the impact of IRE1 kinase inhibition on atherosclerosis. To this end, we fed Apoe^−/−^ mice with WD for 12 weeks and injected them with AMG‐18 or vehicle once daily for the last 4 weeks of WD (Fig 6A). We observed no significant differences in body weight, plasma glucose or TPC, and the number of circulating immune cells between the groups (Appendix Fig S5A–D). Consistent with an earlier publication that determined the effective and non‐toxic dose for AMG‐18 in mice, the inhibitor engaged its molecular target effectively in the treatment group (as assessed by reduced IRE1 autophosphorylation) (Appendix Fig S5E). IRE1 kinase inhibition led to a decrease in atherosclerotic lesions in *en face* aorta preparations (Fig 6B). As shown above for the Fmr1^−/−^ mice, the aortic root lesion area was not different between the groups (Fig 6C), but foam cell area as well as necrotic core area were significantly decreased (Fig 6D and E).

We also assessed the impact of twice daily injections of the same dose of AMG‐18 on Apoe^−/−^ mice that were fed with WD for 12 weeks (Fig 6F). While we noted a decrease in body weight ratio and TPC, there were no significant differences in plasma glucose levels similar to once daily injection (Appendix Fig S5F–H), and the inhibitor engaged its molecular target effectively (as assessed by reduced IRE1 autophosphorylation) (Appendix Fig S5I and J). IRE1 kinase inhibition led to a significant decrease in atherosclerotic lesions in *en face* aorta preparations (Fig 6G). AMG‐18 injection did not alter aortic root lesion area but significantly decreased foam cell area and necrotic core area (Fig 6H–J). Collectively, our findings thus demonstrate that the inhibition of IRE1 kinase activity by a small‐molecule inhibitor or genetic ablation of FMRP, its kinase substrate, in macrophages can reduce the progression of hypercholesterolemia‐induced atherosclerosis.

## Discussion

**Figure 7:**
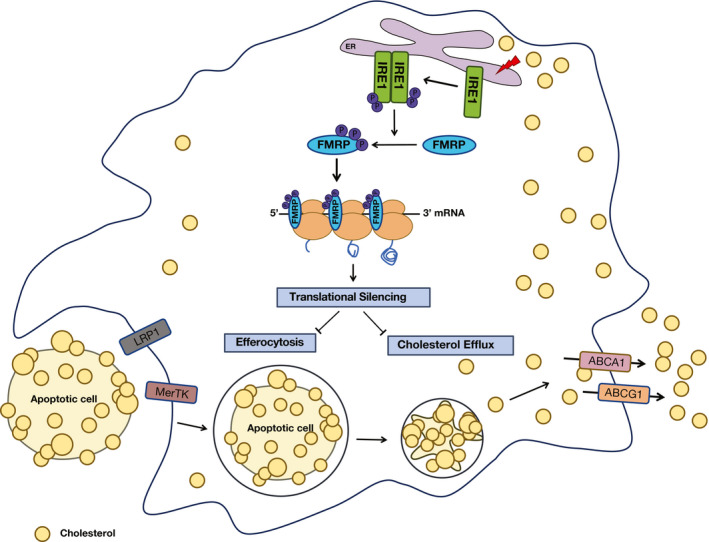
IRE1‐FMRP signaling controls cholesterol efflux and efferocytosis pathways in macrophages IRE1‐mediated FMRP phosphorylation suppresses translation of mRNA for key cholesterol transporters and efferocytosis receptors in macrophages and promotes atherosclerosis.

Chronic lipid accumulation in the ER membranes (e.g., during obesity and in hyperlipidemia) has been shown to impair ER functions and activate UPR signaling (Li *et al*, 2004; Borradaile *et al*, 2006; Fu *et al*, 2011; Volmer *et al*, 2013; Çimen *et al*, 2016b). Numerous studies have shown that ER stress is an important primer for sterile inflammation that drives insulin resistance and atherogenesis (Hotamisligil, 2017). Moreover, alleviating ER stress by inhibiting either PERK or IRE1 signaling prevents atherosclerosis progression (Erbay *et al*, 2009; Tabas, 2010; Tufanli *et al*, 2017; Onat *et al*, 2019). But how does chronic IRE1 kinase activation contribute to the atherosclerotic process? Our work revealed that in ER‐stressed macrophages phosphorylation of FMRP, an RBP functioning as a translational suppressor, is enhanced in an IRE1 kinase‐dependent manner. This phosphorylation event results in a gain‐of‐function for FMRP, which leads to enhanced translational suppression of cholesterol transporters and efferocytosis receptors. Although ubiquitously expressed, prior work focused almost exclusively on FMRP’s role in neurons in the context of its devastating role in FXS pathology (Sethna *et al*, 2014; Davis & Broadie, 2017; Leboucher *et al*, 2019a). Our work reveals a novel function for FMRP in macrophages. Intriguingly, an earlier publication showed that FMRP protein expression is elevated in the macrophage‐enriched area of human atherosclerotic plaques (Tuomisto *et al*, 2003). Moreover, lower cholesterol levels have been measured in a subpopulation of the individuals afflicted with FXS, as well as in Fmr1^−/−^ mice (Berry‐Kravis *et al*, 2015; Lisik *et al*, 2016; Leboucher *et al*, 2019b). Although these findings foreshadowed a role of FMRP in the regulation of cholesterol homeostasis (Tuomisto *et al*, 2003; Darnell *et al*, 2011; Ascano *et al*, 2012), FMRP’s contribution to atherosclerosis was not investigated prior to this study, perhaps because Fmr1 lies on the X chromosome and most genome‐wide association studies in humans focus on variants on autosomal chromosomes excluding sex chromosomes. While characterizing the impact of IRE1 kinase activity and it’s here proposed kinase substrate, FMRP, on macrophage functions, our current study revealed a role for an “IRE1‐FMRP signaling axis” in the regulation of macrophage cholesterol trafficking and efferocytosis, which are among the primary cellular mechanisms that can regress atherosclerosis (Fig 7).

To gain insight into the physiological role of macrophage FMRP, we investigated the consequences of FMRP deficiency on macrophage biology that are relevant to atherosclerosis development and plaque regression, such as foam cell formation, cholesterol efflux, and efferocytosis. Our findings show that FMRP has a prominent role in all three processes. Additionally, our observations are consistent with the finding that several mRNAs encoding cholesterol transporters and efferocytosis receptors interact with FMRP in prior RNA crosslinking immunoprecipitation (CLIP) experiments (Darnell *et al*, 2011; Ascano *et al*, 2012). Our data show that the absence of FMRP in macrophages increases the occupancy of these mRNAs in the polysome fraction, paralleling the increased expression of their corresponding protein products. In agreement with our observations *in cells*, FMRP deficiency in macrophages increased RCT and efferocytosis in mice. Moreover, systemwide and myeloid‐specific Fmr1 knock‐out mice were protected from hypercholesterolemia‐induced atherosclerosis progression. In agreement with this notion, the IRE1 kinase‐specific inhibitor, AMG‐18, phenocopies the beneficial impact of FMRP deficiency (such as enhanced cholesterol efflux and efferocytosis) in macrophages. While our studies provide strong support for the role of macrophage FMRP in atherosclerosis progression, we did not examine the contribution of FMRP activity in other lesion cell types, such as endothelial or vascular smooth muscle cells, on atherosclerosis. The impairment of RCT and efferocytosis by IRE1‐FMRP signaling implies that interventions to ablate FMRP in a macrophage‐specific manner and in the adult organism could promote atherosclerotic plaque stabilization and hinder plaque progression, while escaping an adverse impact of FMRP deficiency on neuronal development.

Two recent studies show that FMRP plays a metabolic role in the liver. For example, systemic FMRP deficiency in mice led to enhanced glucose tolerance and insulin sensitivity, while lowering plasma triglyceride and cholesterol content (Leboucher *et al*, 2019b). Another study showed combined deficiency of FMRP and its paralog FXR2 (Fragile X mental retardation autosomal homolog 2) resulted in lower body fat and increased sensitivity to insulin (Lumaban & Nelson, 2015). Intriguingly, the FMRP loss‐of‐function in liver resulted in increased translation of hepatic mRNA involved in lipid metabolism (Leboucher *et al*, 2019b). In contrast to this previous work, we did not find Fmr1 deficiency‐associated changes in plasma cholesterol or lipoprotein levels in our atherosclerosis mouse models, perhaps due to the severe hypercholesterolemia induced by both genetic and dietary interventions.

In an earlier study, we had shown that small‐molecule inhibitors that are specific for IRE1’s RNase activity prevented lipid‐induced inflammasome activation and secretion of mature interleukin‐1β (m‐IL‐1β) and m‐IL‐18 in both mouse and human macrophages while reducing hyperlipidemia‐induced m‐IL‐1β and m‐IL‐18 production and atherosclerotic plaque size in mice (Tufanli *et al*, 2017). FMRP suppression also reduces m‐IL‐1β secreted from macrophages but without altering inflammasome activation (Appendix Fig S6A–F). Knocking out FMRP from macrophages has no effect on IL‐1β mRNA levels in the whole cell lysate or in the polysomes, suggesting against transcriptional or translational control over these cytokines’ production (Appendix Fig S6G and H). As expected, inhibition of IRE1 kinase also reduces m‐IL‐1β (Appendix Fig S6I–K). Intriguingly, IL‐1β can be secreted through ABCA1 and compete with cholesterol efflux through the same transporter (Tumurkhuu *et al*, 2018). Since the inhibition of IRE1 kinase‐FMRP axis leads to a marked upregulation of cholesterol efflux in macrophages (as shown in our study), it is plausible that increased demand for ABCA1 for cholesterol efflux could prevent IL‐1β secretion through this route.

Our finding that IRE1 phosphorylates FMRP leads to an important question: Why does the UPR signaling impinge on FMRP‐mediated translational suppression? While intensely debated, multiple mechanisms have been proposed for translational suppression by FMRP including polyribosome stalling, miRNA‐mediated translational silencing, and impairing translational initiation (Napoli *et al*, 2008; Edbauer *et al*, 2010; Darnell *et al*, 2011; Chen *et al*, 2014). Recently, phosphorylation‐dependent FMRP‐RNA phase separation has been suggested as a concept underlying these diverse regulatory mechanisms (Tsang *et al*, 2019). It is plausible that during ER stress, IRE1‐induced FMRP phosphorylation, licensed by S500 phosphorylation and then spreading to adjacent serines and threonines, enhances phase separation of FMRP and leads to the redistribution of FMRP‐bound RNA into cytoplasmic granules, where they will not be translated. This would, in effect, reduce the protein folding stress and improve ER homeostasis. Indeed, ER stress induces stress granule formation, which contain mRNAs, RBPs (including FMRP), 40S ribosomal subunits, and translational pre‐initiation factors (Buchan & Parker, 2009).

Finally, our findings demonstrate that IRE1 kinase‐specific inhibitor, AMG‐18, can phenocopy the beneficial impact of FMRP deficiency (such as enhanced cholesterol efflux and efferocytosis) in macrophages. Although we cannot definitively show that FMRP is the only mediator downstream of IRE1 kinase activity in regulating the proposed mechanisms, our data strongly support that this novel IRE1 kinase target’s expression in macrophages plays an important role in cholesterol efflux and apoptotic cell clearance and is an important contributor to atheroprotection offered by IRE1 kinase inhibition.

Our study provides mechanistic insight into the translational regulation of cholesterol efflux and efferocytosis by macrophages during ER stress, highlighting IRE1 and its effector FMRP as a promising molecular target for regulating atherosclerosis. The small‐molecule inhibitors of IRE1 kinase activity represent a translational opportunity in combating atherosclerosis.

## Materials and Methods

### General study design

Three or more independent replicates were performed for cell‐based experiments. Mice were randomly assigned to independent cohorts, and data analysis was performed blind. The only elimination criteria used for mouse studies was based on health and as advised by a veterinarian.

### Reagents and plasmids

WT‐Ire1‐pcDNA3, and Kinase‐dead IRE1 mutant (K599A)‐pcDNA3 plasmids were generated in Dr. Peter Walter’s laboratory (University of California, San Francisco). Fatty acid‐free bovine serum albumin was from Gold Biotechnology (A‐421–250). Lipofectamine 3000 (L3000001) and EDTA (15575020) were purchased from Invitrogen. Polyethylemine (PEI; molecular weight 25,000) was from Polysciences. Neon transfection system kit (MPK10096) was from Thermo Scientific and used with the Neon electroporation system, also from Thermo Scientific, according to previously published protocols for BMDM (Tufanli *et al*, 2017; Onat *et al*, 2019). l‐Glutamine, Dulbecco’s modified Eagle’s Medium (DMEM), penicillin/streptomycin (P/S), fetal bovine serum (FBS), Roswell Park Memorial Institute (RPMI)‐1640 medium, phosphate buffer saline (PBS), CellTrace™ CFSE cell proliferation kit (C34554), CellTrace™ Violet Cell Proliferation Kit (C34557), Fmr1 siRNA (AM16708), Control siRNA (negative control, AM4611), Revert Aid First strand cDNA synthesis kit (K1691), and Formaldehyde (31901) were from Thermo Scientific. Protease inhibitor cocktail (P8340), phosphatase inhibitor cocktail‐3 (P0044), Dimethyl sulfoxide (DMSO, D8418), Trypsin, Ampicillin, palmitic acid (PA; P0500), Oil Red O solution (O1391), Hematoxylin (HHS32), Lipoprotein‐deficient serum (S5519), CC mount (C9368), ECL™ Prime Western Blotting Detection Reagent (GERPN2232), fatty acid‐free BSA (A6003), Sucrose (S0389), and thioglycolate solution (70157) were purchased from Sigma. Dil‐labeled ac‐LDL (770201‐7) was from Kalen Biomedical. The IRE1 Kinase inhibitor, AMG‐18 were from Tocris. Primary antibodies used for immunoblotting were from the following companies: anti‐pIRE1 (phsopho‐S724; 124945), anti‐FMRP (ab17722), anti‐LRP1 (ab92544), anti‐α‐SMA (ab5694), Anti‐IL‐1 beta antibody (ab9722), Anti‐pro Caspase1 + p10 + p12 antibody [EPR16883] (ab179515), and anti‐Thiophosphate ester antibody (ab133473) were purchased from Abcam; anti‐pFMRP (phosphor‐S499; p1125‐499) from PhosphoSolutions; anti‐IRE1 (3294), anti‐LRP1 (64099) and anti‐FMRP (4317) from Cell‐Signaling; anti‐FMRP (NBP02‐01770), anti‐ABCA1 (NB400‐105), and anti‐ABCG1 (NB400‐132) from Novus Biologicals; anti‐β‐Actin‐horse radish peroxidase (47778) and Secondary IgG‐Goat (sc‐2354) from Santa Cruz Biotechnology; anti‐MOMA‐2 from Bio‐Rad; anti‐PE‐F4/80 (123110), anti‐FMRP (834601), Propidium Iodide Solution, PI (421301), Cell Staining Buffer (420201), and TruStain FcX (anti‐CD16/32, 101319) from Biolegend. Active ERN1 human recombinant protein (E31‐11G) was from Biotech. FMRP human recombinant protein (TP322699) was from Origene. Cholesterol Uptake Assay Kit (ab236212), cholesterol efflux assay kit (ab196985), Masson Trichrome (04‐010802), Fluoroshield mounting reagent with DAPI (ab104139), ATP‐γ‐S (ab138911), and p‐Nitrobenzyl mesylate (PNBM, ab138910) were purchased from Abcam. Aqua‐Mount (41799‐008) was from VWR. Cremophor EL (238470) and Cycloheximide (239763) were purchased from EMD Millipore. TUNEL (In situ Cell Death detection Kit, Fluorescenin; 11684795910) was purchased from Roshe. Hematoxylin and Eosin Stain Kit (H‐3502) was purchased from Vector Laboratories. Wako Diagnostics total cholesterol E kit (NC9138103) was purchased from Fujifilm Medical Systems USA 99902601. TRIsure (BIO‐38033) was purchased from Bioline. Power‐Up‐SYBR green (A25742) was purchased from Applied Biosystems. ^3^H‐cholesterol (NET139001MC) was purchased from Perkin Elmer. HBSS (14175095) was purchased from Gibco. Anti‐MERTK (AF‐591‐SP) antibody was purchased from R&D. Secondary IgG‐Rabbit (5220‐0337) and Secondary‐IgG‐Mouse (5220‐0341) were purchased from SeraCare. Lambda phosphatase (λ Phosphatase, PPase) enzyme (sc‐200312) was purchased from Santu Cruz.

### Mice studies and treatments

C57BL/6 (WT, Fmr1^+/+^), Fmr1^−/−^, and Apolipoprotein E‐deficient (Apoe^−/−^) mice were purchased from Jackson Laboratory. IRE1 conditional knock out (Ire1α^flox/flox^) mice were a kind gift from Dr. Takao Iwawaki (Kanazawa Medical University, Japan) and characterized before (Iwawaki *et al*, 2009). Ire1α^flox/flox^ mice were intercrossed from with LysM^cre^ mice purchased from Jackson Lab (004781) to obtain Ire1α^flox/flox^, LysM^cre^ mice (IRE1^−/−^), which had a myeloid‐specific Ire1α gene deletion. Fmr1 conditional knock out (Fmr1^flox/flox^) mice were a kind gift from Dr. David Nelson. Fmr1^flox/flox^ mice were inter‐crossed with LysM^cre^ mice purchased from Jackson Lab (004781) to obtain myeloid Fmr1‐deficient (myFmr1^−/−^) mice.

Starting at 8 weeks of age, Fmr1^+/+^, Fmr1^−/−^, myFmr1^+/+^, or myFmr1^−/−^ mice were injected with 1 × 10^10^ AAV_PCSK9 (AAV8‐D377Y‐mPCSK9, Vector BiolabsAAV‐268246) via tail vein, then fed with normal chow or high cholesterol/high fat atherosclerotic mouse diet from Envigo (TD.88137) for 6–16 weeks. Apoe^−/−^ mice were fed with WD (12 weeks) and intraperitoneally injected with vehicle (DMSO) or AMG‐18 (30 mg/kg) once or twice a day in the last 4 weeks of WD. C57BL/6 were injected with Tunicamycin (TM, 1 mg/kg) and with AMG‐18 (30 mg/kg) or vehicle (DMSO) in 20% vol/vol Cremophor EL saline solution, as described in (Tufanli *et al*, 2017). Eight hours later, peritoneal macrophages were isolated by thioglycolate elicitation for further analysis.

#### Husbandry conditions and study approval

Mice were kept under specific pathogen‐free conditions with food and water *ad libititum*. Both female and male mice were used for experiments. All animal experiments were performed according to protocols approved by the Experimental Animal Ethical Care Committees at Bilkent University, Ankara, Turkey or Cedars Sinai Medical Center, Los Angeles, USA or the University of Ottawa Animal Care Committee, Ottawa, ON K1N 6N5, Canada.

### Macrophage isolation

#### Peritoneal macrophages

3% thioglycolate solution was injected to mice intraperitoneally and peritoneal macrophages were collected 4 days after by washing the peritoneal cavity with ice‐cold PBS (10 ml) as described before (Zhang *et al*, 2008; Onat *et al*, 2019). Cells were centrifuged at 500 *g* for 5 min at 4°C and resuspended in RPMI medium in cell culture plates. Macrophages were incubated at 5% carbon dioxide incubator at 37°C for 30 min to attach and non‐adherent cells were removed along with media. Cells were rinsed with PBS and used for protein isolation or RNA isolation.

#### Bone Marrow‐Derived Macrophages (BMDM)

Bone marrows were collected form the tibia and femurs of mice into RPMI containing 1% Penicillin/streptomycin (P/S) cocktail as previously described (Tufanli *et al*, 2017). After filtering through a cell strainer (BD, 352350), cells were centrifuged at 500 *g* for 5 min and resuspended in RPMI enriched with 20% L929 cells conditioned medium, 10% heat‐inactivated fetal bovine serum (FBS), and 1% P/S cocktail, followed by growth on Petri dishes and differentiation to macrophages for 5–10 days.

### Cell lines

Fmr1^−/−^ mouse embryonic fibroblasts (MEF) were generated in Dr. David Nelson’s laboratory (Baylor College of Medicine, Houston, Texas). HEK293T, Jurkat (human T lymphocytes), and L‐929 (mouse fibroblasts) cells were obtained from ATCC. Cells were cultured in RPMI or DMEM supplemented with 10% heat‐inactivated FBS and %1 P/S cocktail. Cells were cultured in a humidified CO2 incubator at 37°C. All cells were regularly tested for mycoplasma contamination.

### Transfection

60–80% confluent HEK293T, WT or Fmr1^−/−^ MEF cells were transfected using Lipofectamine 3000 or Polyethylemine (PEI). BMDM and HEK293T cells were electroporated either with IRE1‐, Fmr1‐ (100 nM), or control‐siRNA using Neon electroporator (Thermo Scientific) as per specific conditions provided by the manufacturer and as described earlier (Tufanli *et al*, 2017). 24–36 h after transfection, cells were treated with PA or TG to induce ER stress.

### Palmitate (PA)/bovine serum albumin (BSA) complex preparation

PA was dissolved in absolute ethanol to yield a stock concentration of 500 mM and stored at −80°C. Stock PA was diluted to working concentration and suspended with %1 fatty acid‐free BSA in serum‐free RPMI growth medium by mixing at 55°C for 15 min as described before (Erbay *et al*, 2009).

### Western blot analysis

Cells were lysed in lysis buffer (50 mM HEPES pH:7,9, 100 mM NaCl, 10 mM EDTA,10 mM NaF, 4 mM NaPP, 1% Triton, 1 mM phenylmethanesulfonylfluoride (PMSF), 1× phosphatase inhibitor cocktail 3 and 1× (10 µM) protease inhibitor cocktail as described in (Çimen *et al*, 2016b). After centrifugation, clear lysates were mixed with sodium dodecyl sulfate (SDS) loading dye and heated at 95°C for 5 min before loading on SDS–polyacrylamide gel (SDS–PAGE) gels. After separation according to protein molecular weights on these gels, samples were transferred to polyvinylidene difluoride (PVDF) membrane. Blocking and antibody incubation of the membranes were carried out in tris‐buffered saline (TBS) buffer prepared with 0.1% Tween‐20 (v/v) and 5% (w/v) dry milk or BSA. ECL prime reagent was used to develop the membranes, and images were captured with ChemiDoc (BioRad).

#### Antibody dilutions

Anti‐pIRE1 1:2,000, anti‐FMRP 1:2,000, anti‐LRP1 1:5,000, anti‐IL‐1 beta 1:500, Anti‐pro Caspase1 + p10 + p12 1:2,000, anti‐Thiophosphate ester 1:5,000, anti‐pFMRP (phosphor‐S499) 1:2,000, anti‐IRE1 1:2,000, anti‐ABCA1 1:1,000, anti‐ABCG1 1:1,000, Anti‐MERTK 1:1,000, anti‐β‐Actin‐horse radish peroxidase 1:5,000, Secondary IgG‐Goat 1:10,000, Secondary IgG‐Rabbit 1:10,000, Secondary‐IgG‐Mouse 1:10,000.

### RNA isolation and quantitative reverse transcription polymerase chain reaction (qRT‐PCR)

Total RNA was isolated using TRIsure. RNA extractions were than reverse transcribed by using Revert Aid First strand cDNA synthesis kit to complementary deoxyribonucleic acid (cDNA) according to manufacturer’s protocol. Using specific primers, cDNAs were amplified on Rotor Gene (Qiagen). Power‐Up‐SYBR green (Applied Biosystems, A25742) was used for qRT‐PCR reaction. The following PCR primers were used for mRNA expression analysis:

mmu‐Fmr1‐F 5′ CCGAACAGATAATCGTCCACG 3′
mmu‐Fmr1‐R 5′ ACGCTGTCTGGCTTTTCCTTC 3′
mmu‐Abca1‐F 5′ AAAACCGCAGACATCCTTCAG 3′
mmu‐Abca1‐R 5′ CATACCGAAACTCGTTCACCC 3′
mmu‐Abcg1‐F 5′ GGTCCTGACACATCTGCGAA 3′
mmu‐Abcg1‐R 5′ CAGGACCTTCTTGGCTTCGT 3′
mmu‐Mertk‐F 5′ CAGGGCCTTTACCAGGGAGA 3′
mmu‐Mertk‐R 5′ TGTGTGCTGGATGTGATCTTC 3′
mmu‐Lrp1‐F 5′ GCCTACACCTGGAGAGATAGC 3′
mmu‐Lrp1‐R 5′ GGCAACTTACGAGCAGGCT 3′
mmu‐Cd36‐F 5′ GTGCTCTCCCTTGATTCTGC 3′
mmu‐Cd36‐R 5′ CTGCACCAATAACAGCTCCA 3′
mmu‐Cd47‐F 5′ TGGTGGGAAACTACACTTGCG 3′
mmu‐Cd47‐R 5′ CGTGCGGTTTTTCAGCTCTAT 3′
mmu‐Calr‐F 5′ GCAGACCCTGCCATCTATTTC 3′
mmu‐Calr‐R 5′ TCGGACTTATGTTTGGATTCGAC 3′
mmu‐Rac1‐F 5′ ATGCAGGCCATCAAGTGTG 3′
mmu‐Rac1‐R 5′ TAGGAGAGGGGACGCAATCT 3′
mmu‐IL‐1β ‐F 5′ CAACCAACAAGTGATATTCTCCATG 3′
mmu‐IL‐1β ‐R 5′ GATCCACACTCTCCAGCTGCA 3′
mmu‐Gapdh‐F 5′ ATTCAACGGCACAGTCAAGG 3′
mmu‐Gapdh‐R 5′ TGGATGCAGGGATGATGTTC 3′

The following primers were used to introduce site‐directed mutagenesis on FMRP plasmids:

S500A‐F 5′ GCATCAAATGCTGCTGAAGCAGAAGCTGACCACAGAGAC 3′
S500A‐R‐ 5′ GTCTCTGTGGTCAGCTTCTGCTTCAGCAGCATTTGATGC 3′
S500‐T502‐S504A‐F 5′ GCATCAAATGCTGCTGAAGCAGAAGCTGACCACAGAGAC 3′
S500‐T502‐S504A‐R 5′ GTCTCTGTGGTCAGCTTCTGCTTCAGCAGCATTTGATGC 3′

### Identifying phosphorylation sites on hFMRP using Mass spectrometry

Two *in vitro* kinase reactions of hFMRP and ERN1, worth 4.5 µg protein each were methanol‐chloroform precipitated (Wessel & Flügge, 1984). Dried pellets were dissolved in either [1] 8 M urea/100 mM triethylammonium bicarbonate (TEAB, Thermo Scientific 90114), pH 8.5, or [2] 100 mM ammonium acetate (Sigma‐Aldrich A1542), with or without 8 M urea. Proteins were reduced with 5 mM tris(2‐carboxyethyl) phosphine hydrochloride (TCEP‐HCl, Thermo Scientific C4709) and alkylated with 10 mM 2‐chloroacetamide (Sigma‐Aldrich 22790). Proteins dissolved in urea/TEAB were digested at 37°C in 0.8 M urea/100 mM TEAB, pH 8.5, sequentially with 500 ng Trypsin (Promega V5117) for 17 h, followed by 500 ng Endoproteinase GluC (NEB P8100S) for 4.5 h and quenched with formic acid, 5% final concentration, while proteins dissolved in urea/TEAB or urea/ammonium acetate were digested with 200 ng Proteinase K (Sigma‐Aldrich P2308) at 37°C for 30 min and heat quenched at 90°C for 15 min (similar reactions in ammonium acetate without urea were performed for 30 min or 15 min followed by 16 h digestion with trypsin) (Baboo *et al*, 2021). The digest was injected directly onto a 20 cm, 100 µm ID column packed with BEH 1.7 µm C18 resin (Waters 186005225). Samples were separated at a flow rate of 400 nl/min on an nLC 1000 (Thermo LC120). Buffer A and B were 0.1% formic acid in 5% acetonitrile and 0.1% formic acid in 80% acetonitrile, respectively. A gradient of 1–25% B over 110 min, an increase to 40% B over next 20 min, an increase to 90% B over another 10 min, and a hold at 90% B for the final 10 min were used for a total run time of 140 min. The column was re‐equilibrated with 20 µl of buffer A prior to the injection of sample. Peptides were eluted directly from the tip of the column and nano‐sprayed into the mass spectrometer by application of 2.8 kV voltage at back of the column. The Orbitrap Fusion Lumos (Thermo) was operated in data‐dependent mode. Full MS1 scans were collected in the Orbitrap at 120K resolution with a mass range of 400–1,500 *m/z* and an AGC target of 4e5. The cycle time was set to 3 s, and within these 3 s, the most abundant ions per scan were selected for CID MS/MS in the ion trap with an AGC target of 2e4 and minimum intensity of 5,000. Maximum fill times were set to 50 and 35 ms for MS and MS/MS scans, respectively. Quadrupole isolation at 1.6 *m/z* was used, monoisotopic precursor selection was enabled, charge states of 2–7 were selected, and dynamic exclusion was used with an exclusion duration of 5 s. Samples were also analyzed with HCD fragmentation (35 NCE) and detection at 7,500 resolution.

Protein and peptide identification were done with Integrated Proteomics Pipeline—IP2 (Integrated Proteomics Applications). Tandem mass spectra were extracted from raw files using RawConverter (He *et al*, 2015) and searched with ProLuCID (Xu *et al*, 2015) against a concatenated database comprising of amino acid sequences from vendors for FMRP, hERN1, and Endoproteinase GluC, UniProt reference proteome of *Escherichia coli* K12 (UP000000625) Homo sapiens (UP000005640). The search space included all fully tryptic and half‐tryptic peptide candidates (no enzyme specificity for sample treated with Proteinase K). Carbamidomethylation (+57.02146) was considered a static modification on cysteine, and phosphorylation (+79.966331) was considered a differential modification on serine/threonine/tyrosine. Data were searched with 50 ppm precursor ion tolerance and 500 ppm fragment ion tolerance. Identified proteins were filtered to using DTASelect (Tab b *et al*, 2002) and utilizing a target‐decoy database search strategy to control the false discovery rate at 1%, at the spectrum level (Peng *et al*, 2003). A minimum of 1 peptide per protein and 1 tryptic end per peptide (no tryptic ends in case of Proteinase K treatment) were required and precursor delta mass cut‐off was fixed at 10 ppm. Localization scores were assigned to identified sites of phosphorylation using A‐Score (Beausoleil *et al*, 2006).

### Co‐immunoprecipitation and kinase assay

#### Co‐immunoprecipitation

HEK293T cells were co‐transfected with IRE1 and FMRP plasmids for 24 h followed by TG (600 nM) or TM (1 µg/ml) for 2 h. Equal amounts of protein lysates were precipitated with specific antibodies (anti‐IRE1 1:250 and anti‐FMRP 1:250) at 4°C overnight on a rocker. Protein magnetic beads were added to each sample and incubated at 4°C for overnight. Immunoprecipitates were then analyzed by Western blot.

#### Kinase assay

HEK293T cells were transfected with either with WT‐ or KD‐IRE1 plasmids for 24 h, followed by TG (600 nM) for 2 h for IRE1 activation. Equal amounts of protein lysates were then precipitated with specific IRE1 antibody‐coated magnetic beads at 4°C overnight on a rocker. Immunoprecipitates were incubated at 30°C for 45 min in kinase assay buffer (SignalChem K01‐09) with specific ATP analogue (ATP‐γ‐S, 100 µM) and purified hFMRP protein. p‐Nitrobenzyl mesylate (PNBM) and 0.5 M ETDA solution were added to reaction after 45 min and incubated for additional 2 h at RT to alkylate the kinase substrate. Samples were boiled with SDS–PAGE loading dye at 95°C for 5 min to release the proteins from magnetic beads. Beads were separated using magnetic rack and supernatants were analyzed by Western blot.

#### Kinase assay for phospho‐proteomics

Recombinant active IRE1 (500 ng) and FMRP (500 ng) proteins were incubated in kinase buffer at 30°C for 45 min with ATP‐γ‐S (100 µM). Samples were then incubated at 24°C for 1 h with PNBM (2.5 mM). Samples were boiled in SDS loading buffer at 95°C and loaded to SDS–PAGE. Anti‐thiophosphate ester antibody was used to detect alkylated kinase substrate.

### Polysome fractionation

Polysome fractionation protocol was adapted from Stastna *et al* (Stastna *et al*, 2018). Briefly, Fmr1^+/+^ and Fmr1^−/−^ BMDM were treated with PA (500 µM) for 6 h followed by cycloheximide (100 µg/ml) for 10 min prior to lysis with buffer (100 mM KCl, 20 mM Tris pH 7.5, 5 mM MgCl_2_, 0.4% NP‐40, 100 µg/ml cycloheximide, 0.1 U RNase inhibitor and protease inhibitor cocktail). Clear lysates were loaded to 10–50% sucrose gradient (in Beckman Coulter Thinwall, Ultra‐Clear tubes, 344059) and centrifuged (in Beckman LE‐80K) for 120 min at 28,4061 *g* at 4°C in a swinging bucket rotor (Beckman SW41) with no‐brake. Each gradient was collected as 17 fractions in microcentrifuge tubes with continuous monitoring of absorbance at 254 nm (Biologic LP (pump), Biorad 731‐8300; BioFrac, Biorad 741‐0002) and frozen immediately at −80°C for further analysis.

### Cholesterol efflux and foam cell formation assays

#### Cholesterol efflux assay

Efflux assay were performed according to manufacturer’s instructions (ab196985). Briefly, macrophages were pre‐loaded with fluorescently labeled cholesterol for 16 h in RPMI media including ACAT inhibitor (5 mg/ml), followed by incubation in efflux medium including cholesterol acceptors apolipoprotein A1 (APOA1; 25 µg/ml) or high‐density lipoprotein (HDL; 50 µg/ml) for 6 h. % Efflux was calculated as cholesterol signal in medium/cholesterol signal in medium and cell.

#### *In vitro* foam cell formation assay

BMDM were incubated with RPMI containing dil‐labeled ac‐LDL (25 µg/ml), 10% lipoprotein‐deficient serum, and 20% l‐Glutamine for 24 h. After cholesterol loading, cells were rinsed with PBS and collected in 2% BSA in PBS. Flow cytometry was performed on a BD Fortessa using FACSDiva software with single stain compensation controls acquired on the same day.

#### *In vivo* foam cell formation assay

Fmr1^+/+^ and Fmr1^−/−^ mice were injected with a gain‐of‐function mutant (D377Y) of proprotein convertase subtilisin kexin 9 (PCSK9)‐encoding adeno‐associated virus (AAV_mPCSK9) and fed with 16 weeks of WD to induced hypercholesterolemia. Apoe^−/−^ mice were fed with 12 weeks of WD with 4 weeks of AMG‐18 (30 mg/kg, once a day) injection during the last 4 weeks of WD. The peritoneal macrophages were collected and assessed for lipid accumulation by Oil‐Red O and Hematoxylin staining.

### Reverse cholesterol transport (RCT) assay

#### Preparation of radiolabel cholesterol

Ag‐LDL (50 µg/ml, made in house with endotoxin‐free LDL isolated from human plasma) and [^3^H]‐cholesterol (5 µCi/ml) were incubated for 1 h at 37°C in a sterile endotoxin free bottle.

#### RCT assay

Fmr1^+/+^ and Fmr1^−/−^ BMDM were incubated with radiolabeled ag‐LDL for 30 h followed by warm HBSS wash and equilibration in 2 mg/ml fatty acid‐free BSA overnight. Cells were washed twice in ice cold HBSS and incubated with EDTA (5 mM) for 20 min at 4°C and spun down at 200 *g* for 5 min. Cells were resuspended in ice cold DMEM and injected into C57BL6N mice subcutaneously in the scruff of the neck. Blood was collected at 24 h via the saphenous vein and at 48 h via cardiac puncture of anesthetized mice. Plasma was used for liquid scintillation counting. At 48 h, livers were removed for scintillation counting. Feces were collected over a 48‐h period, and total feces radioactivity was measured. All [^3^H]‐tracer measurements are expressed relative to the injected amount.

### *In vitro* and *in vivo* efferocytosis

#### Induction of apoptosis and labeling of Jurkat cells

Jurkat cells were fluorescently labeled with CellTrace CSFE or Violet (2 µM) in PBS for 20 min. Cells were then washed ones with PBS and seeded in conditioned DMEM medium followed by irradiation under a 254 nm UV lamp for 5 min. Cells were incubated under normal cell culture conditions for 3–4 h. Apoptosis was confirmed by Anexin V^+^ staining (minimum 85% Annexin V^+^ cells). The apoptotic cells (ACs) were centrifuged at 500 *g* for 5 min and resuspended in conditioned DMEM for experiments.

#### *In vitro* efferocytosis

Bone marrow‐derived macrophages were plated in six‐well dishes at a density of 0.5 × 10^6^ cells per well. CSFE‐labeled ACs were incubated with the macrophages for 2–4 min at a 5:1 AC:macrophage ratio followed by washing three times with PBS. Some groups of macrophages were then incubated for another 2 h in normal cell culture media, followed by the addition of Violet‐labeled ACs. After 2 h, macrophages were washed three times with PBS to remove unbound ACs, and then the macrophages were fixed with 4% formaldehyde for 20 min, rinsed three times with PBS, blocked by TruStain FcX™ (anti‐mouse CD16/32) for 10 min and then stained with PE‐F4/80 o/n. The percentage of PE‐F4/80^+^ and CFSE^+^ double positive cells to total PE‐F4/80^+^ cells was reported as % efferocytosis and PE‐F4/80^+^, CFSE^+^ and Violet^+^ triple positive cells to PE‐F4/80^+^ and CFSE^+^ double positive cells was reported as % continuous efferocytosis.

#### *In vivo* efferocytosis

Fmr1^+/+^ or Fmr1^−/−^ mice were fed with WD for 16 weeks and injected with 1 × 10^6^ CFSE‐labeled ACs and 1.5 h later subsequently peritoneal lavages were collected and stained for PE‐F4/80^+^ resident macrophages. The percentage of PE‐F4/80^+^ and CFSE^+^ double positive cells to total PE‐F4/80^+^ cells was reported as % efferocytosis. Another group of C57BL/6 was injected with AMG‐18 (30 mg/kg) or vehicle (DMSO). After 8 h both groups were interperitoneally injected with CFSE‐labeled ACs and 1.5 h later peritoneal lavages were collected and cultured for 30 min to allow cells to attach. Macrophages were washed three times with PBS to remove unbound ACs, and then the macrophages were fixed with 4% formaldehyde for 20 min, rinsed three times with PBS, blocked by TruStain FcX™ (anti‐mouse CD16/32) for 10 min, and then stained with PE‐F4/80 o/n. The percentage of F4/80^+^ and CFSE^+^ double positive cells to total F4/80^+^ cells was reported as % efferocytosis.

### *En face* Oil‐Red O staining

Aortas opened longitudinally were rinsed with 60% isopropanol for 1 min, stained with Oil‐Red O solution for 20 min, and then distained in 60% isopropanol for 1 min and rinsed in PBS. The lesion area was quantitated as percent of Oil‐Red O staining area in total aorta area.

### Immunohistochemistry

7‐μm‐thick aortic root cryosections (from OCT embedded heart tissue) were stained with antibodies for: anti‐MOMA‐2 (1:500) and anti‐α‐SMA (1:500) and images were captured with fluorescent microscope. Cryosections were stained with Masson’s Trichrome, TUNEL, Hematoxylin and Eosin (H&E) according to manufacturer’s instructions. Cryosections were stained with H&E for morphometric lesion analysis. The total lesion area and necrotic area were quantified as previously described from 4 sequential sections (60 µM apart, beginning at the base of the aortic root) as previously described (Çimen *et al*, 2016b). Foam cell area was calculated from Oil‐Red O stained 4 sequential sections (60 µM apart, beginning at the base of the aortic root) and collagen content from Masson’s Trichrome stained sections using ImageJ as previously described (Çimen *et al*, 2016b).

The fluorescent immunostainings were carried out on cryosections that were fixed in cold acetone for 10 min, blocked in goat serum/BSA/PBS as previously described. All stained sections were mounted with fluoroshield mounting reagent with DAPI. Fluorescent signal calculations: (i) TUNEL staining: the sections were double stained with MOMA‐2 to mark the macrophage‐enriched area. The Mean Fluorescent Intensity (MFI) corresponding to primary antibody signal was calculated from the MOMA‐2‐positive area. The background fluorescence of the non‐stained area inside the lesion was subtracted from the total MFI corresponding to each signal (ii) α‐SMA staining: α‐SMA positive area was calculated from the plaque area. The background fluorescence of the non‐stained area inside the lesion was subtracted from the total MFI corresponding to each signal. Data were quantified as total MFI signal compared with baseline (Çimen *et al*, 2016b; Tufanli *et al*, 2017).

### Apoptosis detection by flow cytometry

Apoptosis was induced after treatments by PA (500 µM) treatment for 12 h. Fc receptors were blocked by pre‐incubating cells with 0.25 µg of TruStain FcX™ PLUS (anti‐mouse CD16/32) Antibody per 10^6^ cells for 5–10 min on ice. Cells were then incubated with PI on ice for 20 min in the dark followed by 2× with 2 ml of cell staining buffer. Cells were resuspended in 500 µl of cell staining buffer and analyzed on a BD Fortessa using FACSDiva software with single stain compensation controls acquired on the same day. Data were analyzed using FlowJo analysis software (FlowJo, LLC).

### Flow cytometric analysis of peripheral blood

100 µl of blood was collected in EDTA‐coated tubes and red blood cells were removed by incubation (3×) in Ammonium‐Chloride‐Potassium (ACK, Thermo Fisher A1049201) solution for 5 min at room temperature. Peripheral blood mononuclear cells were then resuspended in FACS buffers (2% BSA in PBS) and incubated for 20 min on ice with the following antibodies: anti‐CD45‐Pac. Blue (clone 30‐F11), CD3e‐PE (Clone 145‐2C11), CD11b‐APC (Clone M1/70), CD19‐BV650 (Clone 6D5), Ly6C‐PE/Dazzle (Clone HK1.4), and Ly6G‐PerCP Cy5.5 (1A8) in 1:100 dilution ratio. Stained samples were washed once and resuspended in FACS buffer containing DAPI (4 µg/ml). Flow cytometry was performed on a BD Fortessa using FACSDiva software with single stain compensation controls acquired on the same day. Data were analyzed using FlowJo analysis software (FlowJo, LLC). All antibodies were purchased from Biolegend (San Diego, CA) and used at the manufacturer’s recommended concentrations.

### Plasma lipids and lipoprotein analysis

Plasma was analyzed by FPLC in the Department of Internal Medicine/Lipid Science, Wake Forest University School of Medicine Winston‐Salem, NC 27019 as described (Çimen *et al*, 2016b). The total cholesterol and triglyceride measurement were performed using WAKO Cholesterol E kit according to the manufacturer’s instructions.

### Statistics

Results are reported as mean ± SEM and statistical significance was determined with Unpaired *t*‐test with Welch’s or Mann–Whitney correction test by GraphPad Software, LLC.

## Author contributions

**Zehra Yildirim:** Data curation; Formal analysis; Validation; Investigation; Visualization; Methodology; Writing—original draft; Writing—review & editing. **Sabyasachi Baboo:** Formal analysis; Investigation; Methodology; Writing—review & editing. **Syed Muhammad Hamid:** Formal analysis; Investigation; Methodology. **Asli Ekin Dogan:** Formal analysis; Investigation; Visualization; Methodology. **Ozlem Tufanli:** Formal analysis; Investigation; Methodology. **Sabrina Robichaud:** Formal analysis; Investigation; Methodology. **Christina Emerton:** Formal analysis; Investigation; Methodology. **Jolene K Diedrich:** Formal analysis; Investigation; Methodology. **Hasan Vatandaslar:** Formal analysis; Investigation; Methodology. **Fotis Nikolos:** Formal analysis; Investigation; Methodology. **Yanghong Gu:** Investigation; Methodology. **Takao Iwawaki:** Methodology. **Elizabeth J Tarling:** Formal analysis; Supervision; Funding acquisition; Investigation; Methodology; Writing—review & editing. **Mireille Ouimet:** Formal analysis; Supervision; Investigation; Methodology; Writing—review & editing. **David Nelson:** Formal analysis; Supervision; Investigation; Methodology; Writing—review & editing. **John R Yates III:** Data curation; Formal analysis; Supervision; Investigation; Writing—review & editing. **Peter Walter:** Formal analysis; Investigation; Writing—review & editing. **Ebru Erbay:** Conceptualization; Formal analysis; Supervision; Funding acquisition; Investigation; Writing—original draft; Project administration; Writing—review & editing.

## Disclosure and competing interests statement

PW is an inventor on U.S. Patent 9708247 held by the Regents of the University of California that describes ISRIB and its analogs. Rights to the invention have been licensed by UCSF to Calico. EE, SMH, OT, and PW will become employees of Altos Labs. All other authors declare that they have no competing interests.

## Supporting information



Appendix

Source Data for Appendix

Source Data for Figure 1

Source Data for Figure 2

Source Data for Figure 3

Source Data for Figure 4

Source Data for Figure 5

Source Data for Figure 6

## Data Availability

All data are available in the main text or the supplementary materials. Research materials used in the article can be requested from authors. The mass spectrometry proteomics data have been deposited to the ProteomeXchange Consortium (Deutsch *et al*, 2020) via the PRIDE (Perez‐Riverol *et al*, 2016, 2019) partner repository with the dataset identifier PXD030594 (http://www.ebi.ac.uk/pride/archive/projects/PXD030594).
