# Supplementary material for: Intercepting IRE1 kinase‐FMRP signaling prevents atherosclerosis progression
Source: EMBO Mol Med. 2022 Feb 22;14(4):e15344. doi: 10.15252/emmm.202115344 (PMC8988208; doi:10.15252/emmm.202115344)
Supplement: Supplementary file 1 — Appendix [file EMMM-14-e15344-s007.pdf]

Supplementary Materials for

Title: Intercepting IRE1 Kinase-FMRP Signaling Prevents Atherosclerosis Progression

Authors: Zehra Yildirim[1,2], Sabyasachi Baboo[3], Syed M. Hamid[1], Asli E. Dogan[1,2], Ozlem Tufanli[4], Sabrina Robichaud[5], Christina Emerton[5], Jolene K. Diedrich[3], Hasan Vatandaslar[6], Fotis Nikolos[7], Yanghong Gu[8], Takao Iwawaki[9], Elizabeth Tarling[10], Mireille Ouimet[5], David L. Nelson[8], John R. Yates III[3], Peter Walter[10], Ebru Erbay[1,11]*

This PDF file includes:

Figures and Figure Legends for Appendix Fig.S1-S6

Appendix Figure S1

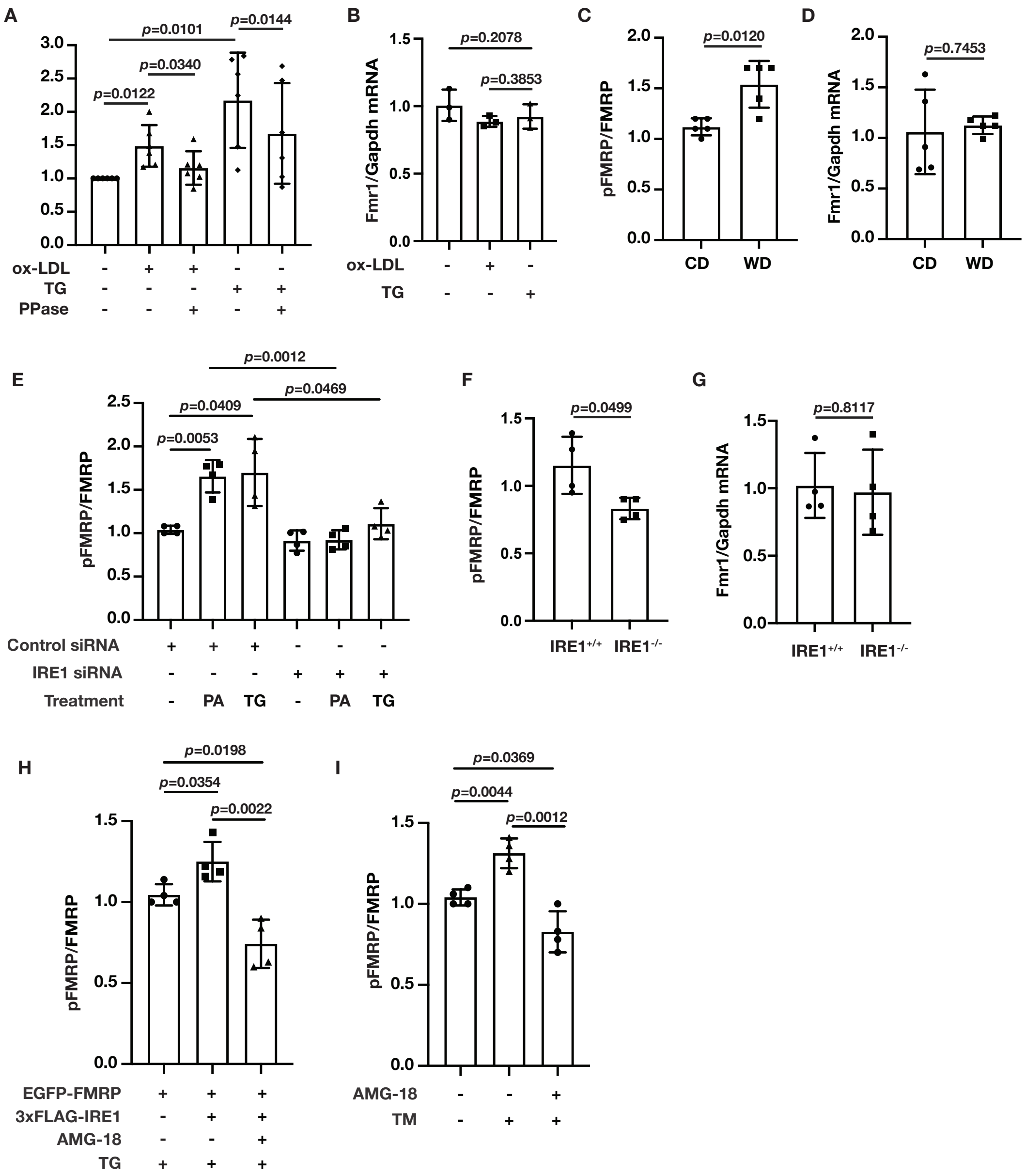

J

```
        10         20         30         40         50         60         70         80         90        100
MEELVVEVRG SNGAFYKAFV KDVHEDSITV AFENNWQPDR QIPFHDVRFP PPVGYNKDIN ESDEVEVYSR ANEKEPCCWW LAKVRMIKGE FYVIEYAACD

       110        120        130        140        150        160        170        180        190        200
ATYNEIVTIE RLRSVNPNKP ATKDTFHKIK LDVPEDLRQM CAKEAAHKDF KKAVGAFSVT YDPENYQLVI LSINEVTSKR AHMLIDMHFR SLRTKLSLIM

       210        220        230        240        250        260        270        280        290        300
RNEEASKQLE SSRQLASRFH EQFIVREDLM GLAIGTHGAN IQQARKVPGV TAIDLDEDTC TFHIYGEDQD AVKKARSFLE FAEDVIQVPR NLVGKVIGKN

       310        320        330        340        350        360        370        380        390        400
                                                                  p
GKLIQEIVDK SGVVRVRIEA ENEKNVPQEE EIMPPNSLPS NNSRVGPNAP EEKKHLDIKE NSTHFSQPNS TKVQRVLVAS SVVAGESQKP ELKAWQGMVP
                                                                 362

       410        420        430        440        450        460        470        480        490        500
                                                                                                   p  p  p
FVFVGTKDSI ANATVLLDYH LNYLKEVDQL RLERLQIDEQ LRQIGASSRP PPNRTDKEKS YVTDDGQGMG RGSRPYRNRG HGRRGPGYTS GTNSEASNAS
                                                                                                        500

       510        520        530        540        550        560        570        580        590        600
 p p                                                                                              p p
ETESDHRDEL SDWSLAPTEE ERESFLRRGD GRRRGGGGRG QGGRGRGGGF KGNDDHSRTD NRPRNPREAK GRTTDGSLQI RVDCNNERSV HTKTLQNTSS
                                                                                                 592

       610        620        630        640        650        660
EGSRLRTGKD RNQKKEKPDS VDGQQPLVNG VPTRTRPLEQ KLISEEDLAA NDILDYKDDD DKV
                                 __________C-Myc/DDK-Tag__________
```

Note on the marks: in the original, "p" marks the phosphorylated residues. The lone mark in block 361–370 stands over S362. Reading the three marks in block 491–500 as S494, S497 and S500, and the two in block 501–510 as T502 and S504, is an inference from the positions in panel K. The two marks in block 591–600 stand over T592 and T594.

K

AA	Sequence	z	XCorr	DeltaCN	[M+H]$^+$	A-Score
*p*S362	HLDIKEN*pS*THFSQPNSTK	3	2.8194	0.3306	2162.9890	14.23
	KHLDIKEN pSTHFSQPNSTK	3	3.2526	0.3569	2291.0808	33.7
	KHLDIKEN*pS*THFSQPNSTK	4	3.4197	0.2304	2291.0813	34.46
*p*S494	RGPGYTSGTN*pS*EASNA	2	3.0995	0.4448	1648.6614	13.27
*p*S497	RGPGYTSGTNSEA*pS*NASETESDHRDELSDWSLAPTEEERE	4	3.457	0.3924	4476.8633	7.81
*p*S500	*pS*ETESDHRDELSDW	2	3.1722	0.3710	1785.6604	22.85
	NA*pS*ETESDHRDELSDW	2	3.1225	0.3763	1970.7406	30.93
**>**	**NA*pS*ETESDHRDELSDW**	**2**	**4.0257**	**0.4280**	**1970.7393**	**39.56**
	NA*pS*ETESDHRDELSDW	2	3.3907	0.3348	1970.7386	33.68
*p*T502	RGPGYTSGTNSEASNASE*pT*ESDHRDELSDWSLAPTEEERE	4	6.2067	0.5107	4476.8564	4.6
*p*S504	SETE*pS*DHRDELSDW	2	3.431	0.3944	1785.6588	15.5
*p*T592	SVH*pT*KTLQNTSSEGSR	3	3.996	0.2936	1811.8281	6.17
	SVHpTKTLQNTSSEGSR	3	4.4606	0.3795	1811.8282	7.53
	SVH*pT*KTLQNTSSEGSR	3	3.5417	0.3859	1811.8276	11.79
*p*T594	SVHTK*pT*LQNTSSEGSR	3	3.1378	0.3386	1811.8282	10.36

Appendix Figure S1. Supplemental figure related to Figure 1.

A Western blot quantifications for pFMRP/FMRP ratio in Fig 1C.

B qRT-PCR analysis of Fmr1 mRNA from the samples in Fig 1C (n=4 biological replicates).

C Western blot quantifications for pFMRP/FMRP ratio in Fig 1D.

D qRT-PCR analysis for Fmr1 mRNA from the samples in Fig 1D (n=3 biological replicates).

E Western blot quantifications for pFMRP/FMRP ratio in Fig 1E.

F Western blot quantifications for pFMRP/FMRP ratio in Fig 1F.

G qRT-PCR analysis for Fmr1 mRNA from the samples in Fig 1F (n=6 biological replicates).

H Western blot quantifications for pFMRP/FMRP ratio in Fig 1G.

I Western blot quantifications for pFMRP/FMRP ratio in Fig 1H.

J pFMRP sequence covered (83%) with two different digestion strategies – Proteinase K (red text) and Trypsin-GluC (highlighted yellow) using LC-MS/MS in samples from Fig 1H.

K LC-MS/MS identified phosphorylated peptides from the samples in Fig 1H; with sites of phosphorylation (AA), charge state (z), identification parameters (XCorr and DeltaCN – greater values indicate higher confidence by peptide-spectrum match/PSM), measured precursor mass ([M+H]+), and a confidence score for localization of phosphorylation on an amino acid (A-Score, value > 19 and > 13, represent > 99% and > 95% confidence, respectively); the peptide marked with (>) was evaluated further.

Data information: Data are mean ± SEM. Unpaired *t*-test with Welch's correction.

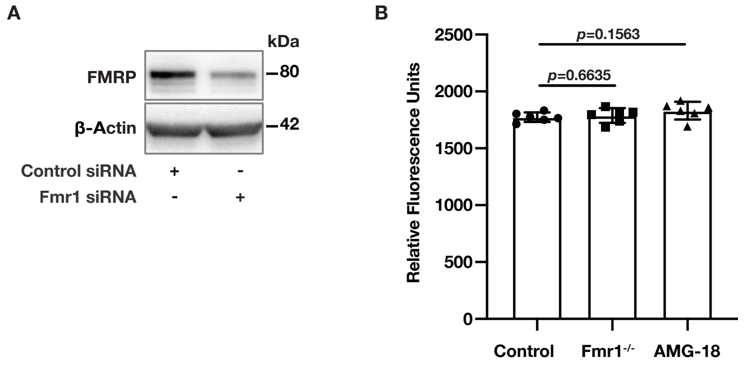

Appendix Figure S2. Supplemental figure related to Figure 2.

A Protein lysates from macrophages used in Fig 2C were analyzed by western blotting using specific antibodies for FMRP and β-Actin.

B Fmr1$^{-/-}$ or AMG-18 pre-treated cells (10 µM, 1 hour) were treated with fluorescently labeled cholesterol for 4 hours (n=6 biological replicates).

Data information: Data are mean ± SEM. Unpaired *t*-test with Welch's correction.

Appendix Figure S3

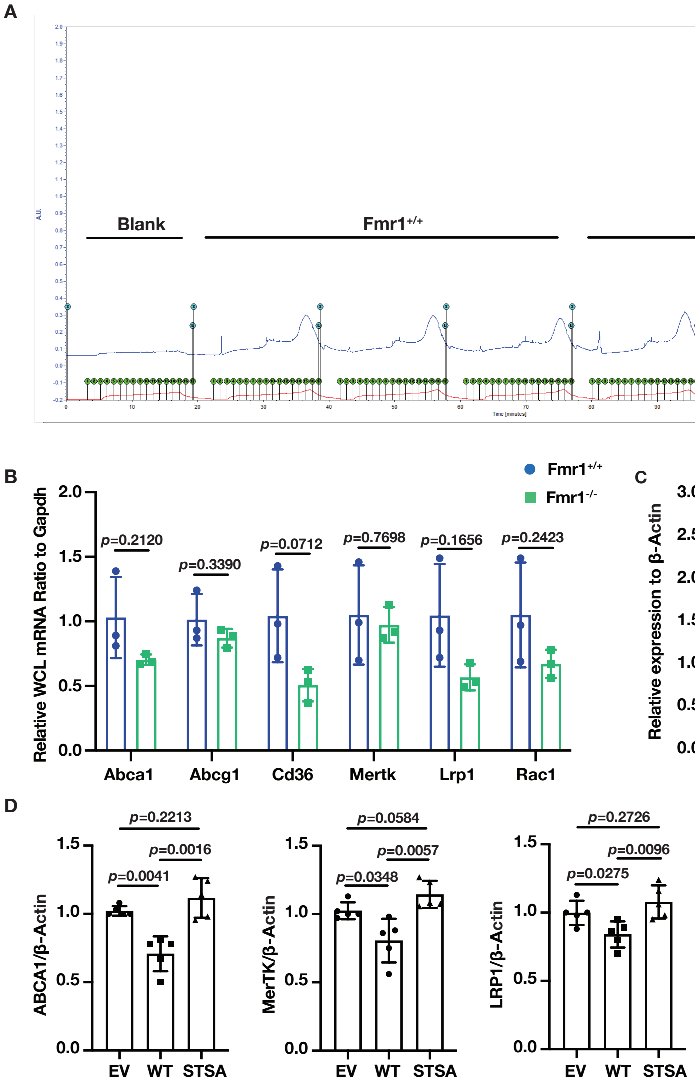

Appendix Figure S3. Supplemental figure related to Figure 4.

A mRNA distribution of fractions from Fmr1$^{+/+}$ and Fmr1$^{-/-}$ RNA samples after sucrose gradient.

B qRT-PCR analysis of Abca1, Abcg1, Mertk, Lrp1, Cd36 and Rac1 in total mRNA levels from same samples used in polysome fractions (n=3 biological replicates).

C Western blot quantifications for ABCA1, ABCG1, MerTK and LRP1 in Fig 4C. The fold change of protein expression level was calculated relative to β-Actin.

D Western blot quantifications for ABCA1, MerTK and LRP1 in Fig 4D. The fold change of protein expression level was calculated relative to β-Actin.

Data information: Data are mean ± SEM. Unpaired t-test with Welch's correction.

Appendix Figure S4

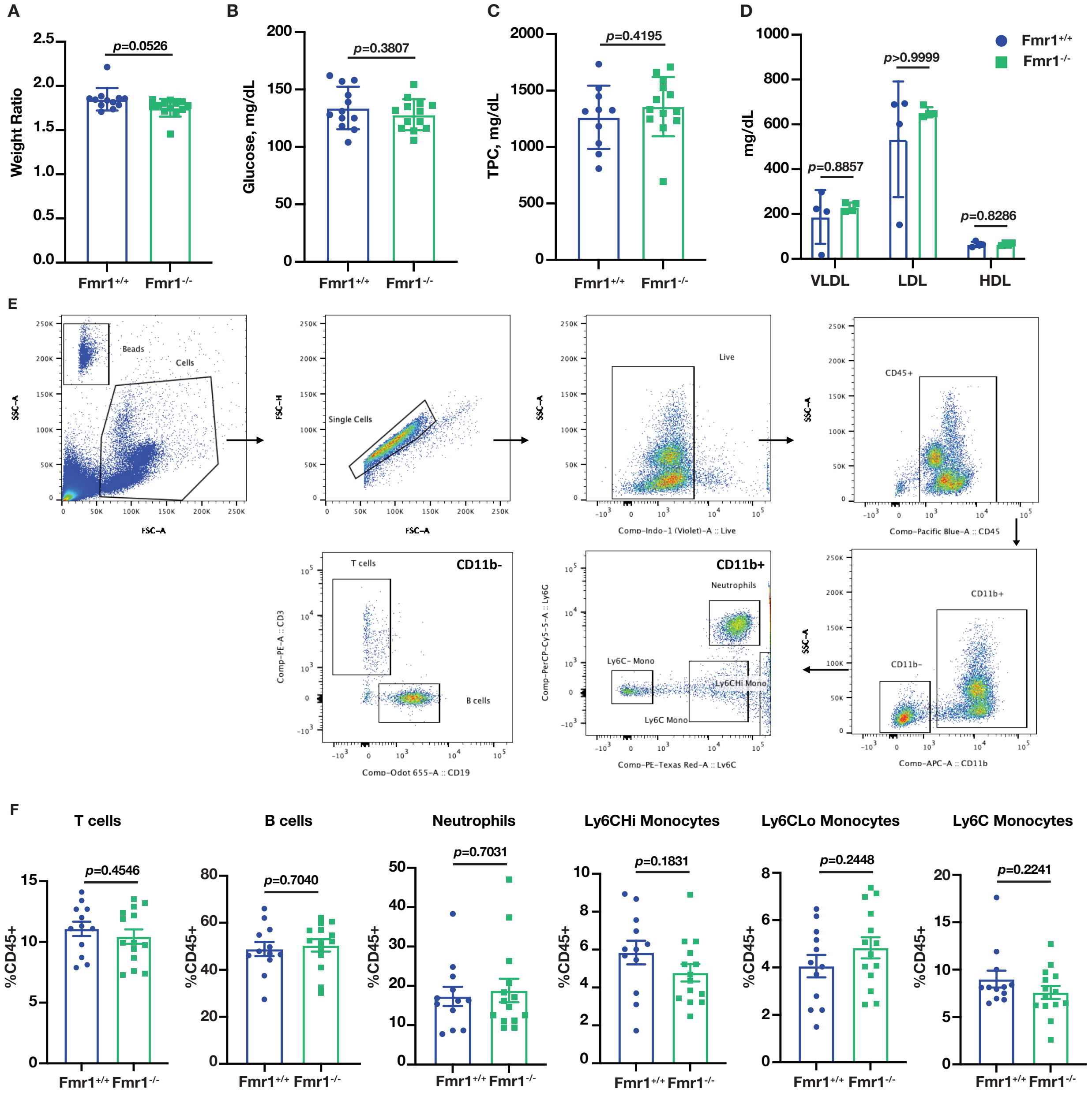

Appendix Figure S4_cont.

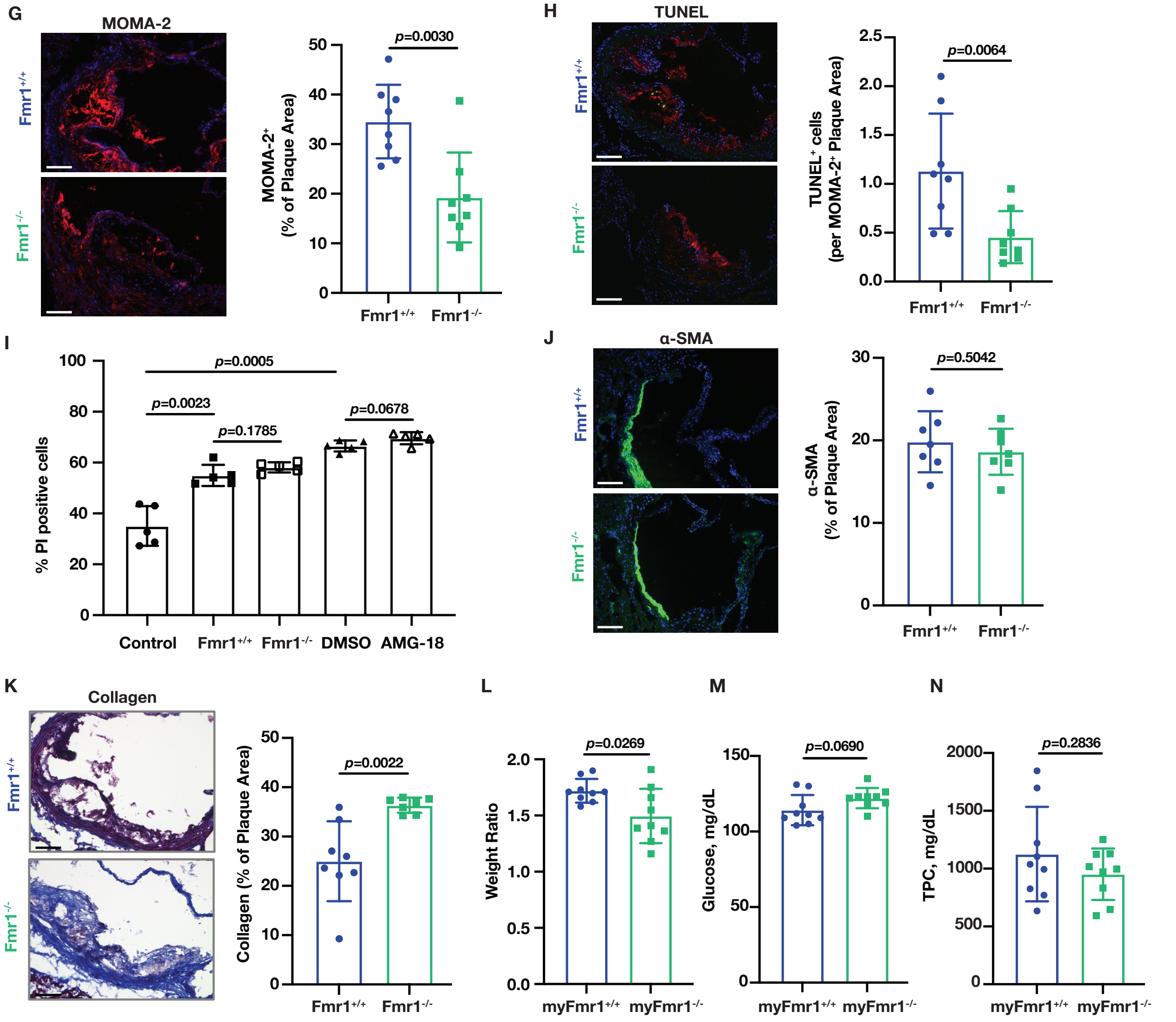

Appendix Figure S4. Supplemental figure related to Fig 5.

A The ratio of mouse weight at the beginning of the experiment over the weight at the end of the experiment in Fig 5A (n=12-13 mice per group).

B-D Plasma measurements for (B) glucose (n=12-13 mice per group), (C) cholesterol (n=12-13 mice per group) and (D) lipoproteins (n=4 mice per group) from mice in Fig 5A.

E Gating strategy for the flow cytometry analysis of mouse peripheral blood.

F Abundance of B cells, T cells and monocytes in the peripheral blood as % of total $CD45^{+}$ cells from the $Fmr1^{+/+}$ and $Fmr1^{-/-}$ mice in Fig.5A (n=12-13 mice per group).

G Macrophage area was calculated from MOMA-2 (red)-stained aortic root sections as % of $MOMA2^{+}$ stained area to total plaque area (n=8 mice per group; Scale bar = 100 µm).

H Apoptosis was calculated from the number of $TUNEL^{+}$ cells (green) in the MOMA-2-stained (red) plaque area (n=8 mice per group; Scale bar = 100 µm).

I $Fmr1^{+/+}$ and $Fmr1^{-/-}$ BMDMs or AMG-18 pre-treated cells (10 µM, 1 hour) were treated with PA (500 µM) treatment for 12 hours and then stained with Propidium iodide (PI) (n=5 biological replicates).

J % Smooth muscle actin (α-SMA) was calculated from aortic root sections stained with α-SMA (green) as % $\alpha\text{-SMA}^{+}$ area to the total plaque area (n=8 mice per group, Scale bar 100 µm).

K % collagen area was calculated from Masson's Trichrome staining as the percentage of collagen (blue) area in total plaque area (n=8 mice per group; Scale bar = 100 µm).

L The ratio of mouse weight at the beginning of the experiment over the weight at the end of the experiment in Fig. 5F (n=9 mice per group).

M-N Plasma measurements for (M) glucose (n=9 mice per group) and (N) cholesterol (n=9 mice per group) from mice in Fig 5F.

Data information: Data are mean ± SEM; Mann Whitney U test.

Appendix Figure S5

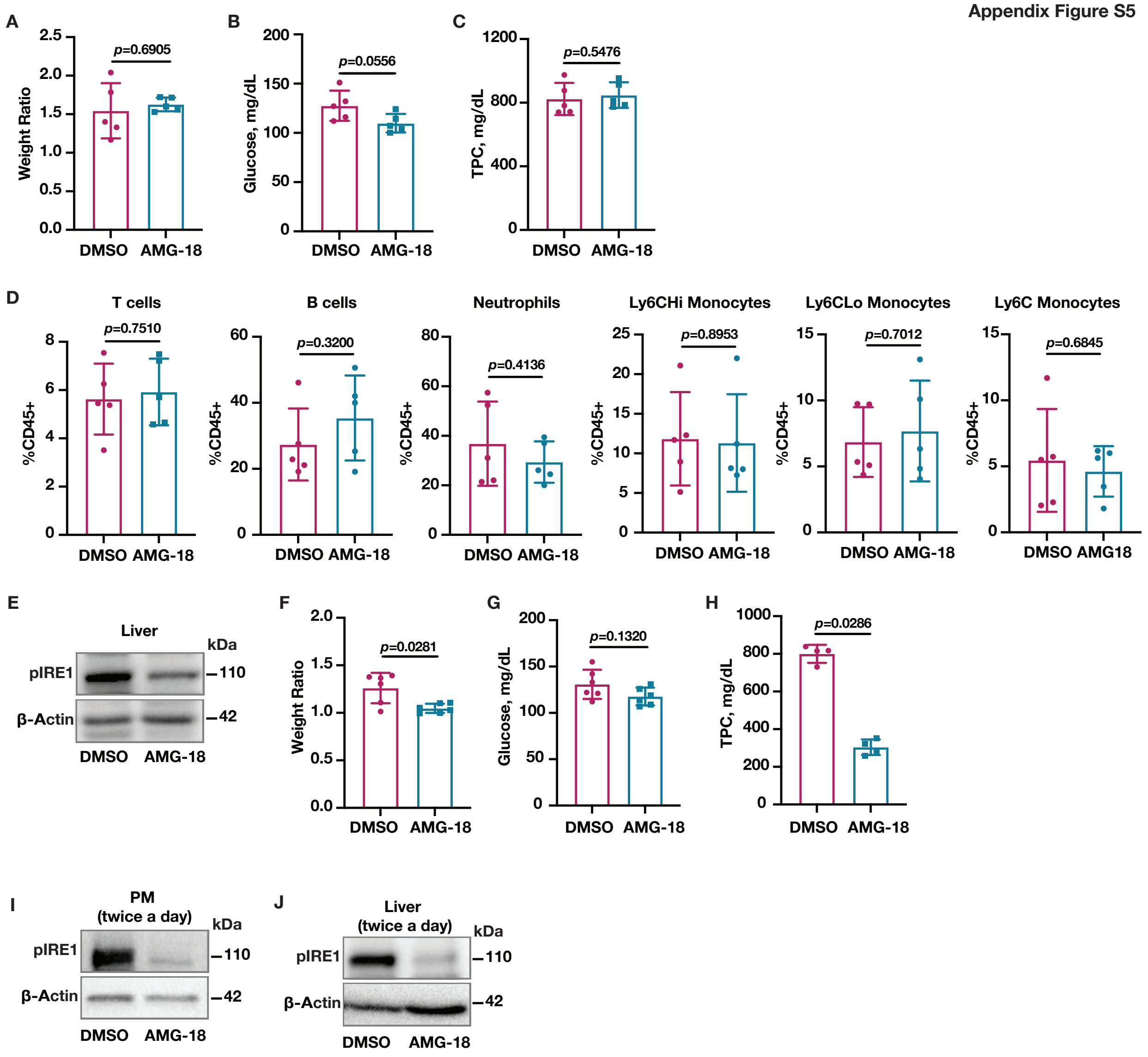

Appendix Figure S5. Supplemental figure related to Fig 6.

A The ratio of mouse weight at the beginning of the experiment over the weight at the end of the experiment in Fig. 6A (n=5 mice per group).

B-D Plasma measurements for (B) glucose (n=5 mice per group) and (C) cholesterol (n=5 mice per group) from mice in Fig. 6A. (D) Abundance of B cells, T cells and monocytes in the peripheral blood as % of total $CD45^+$ cells from the vehicle (DMSO) or AMG-18 (30 mg/kg) once a day injected mice in Fig. 6A (n=5 mice per group).

C Protein lysates of liver from mice in Fig. 6A. Proteins were analyzed by western blotting using specific antibodies for pIRE1 and β-Actin (n=5 mice per group).

F The ratio of mouse weight at the beginning of the experiment over the weight at the end of the experiment in Fig. 6F (n=6 mice per group).

G-H Plasma measurements for (G) glucose (n=6 mice per group) and (H) cholesterol (n=6 mice per group) from Fig 6F.

I-J Protein lysates of (I) thioglycolate-elicited PM and (J) liver from mice in Fig 6F. Proteins were analyzed by western blotting using specific antibodies for pIRE1 and β-Actin (n=6 mice per group).

Data information: Data are mean ± SEM; Mann Whitney U test.

Appendix Figure S6

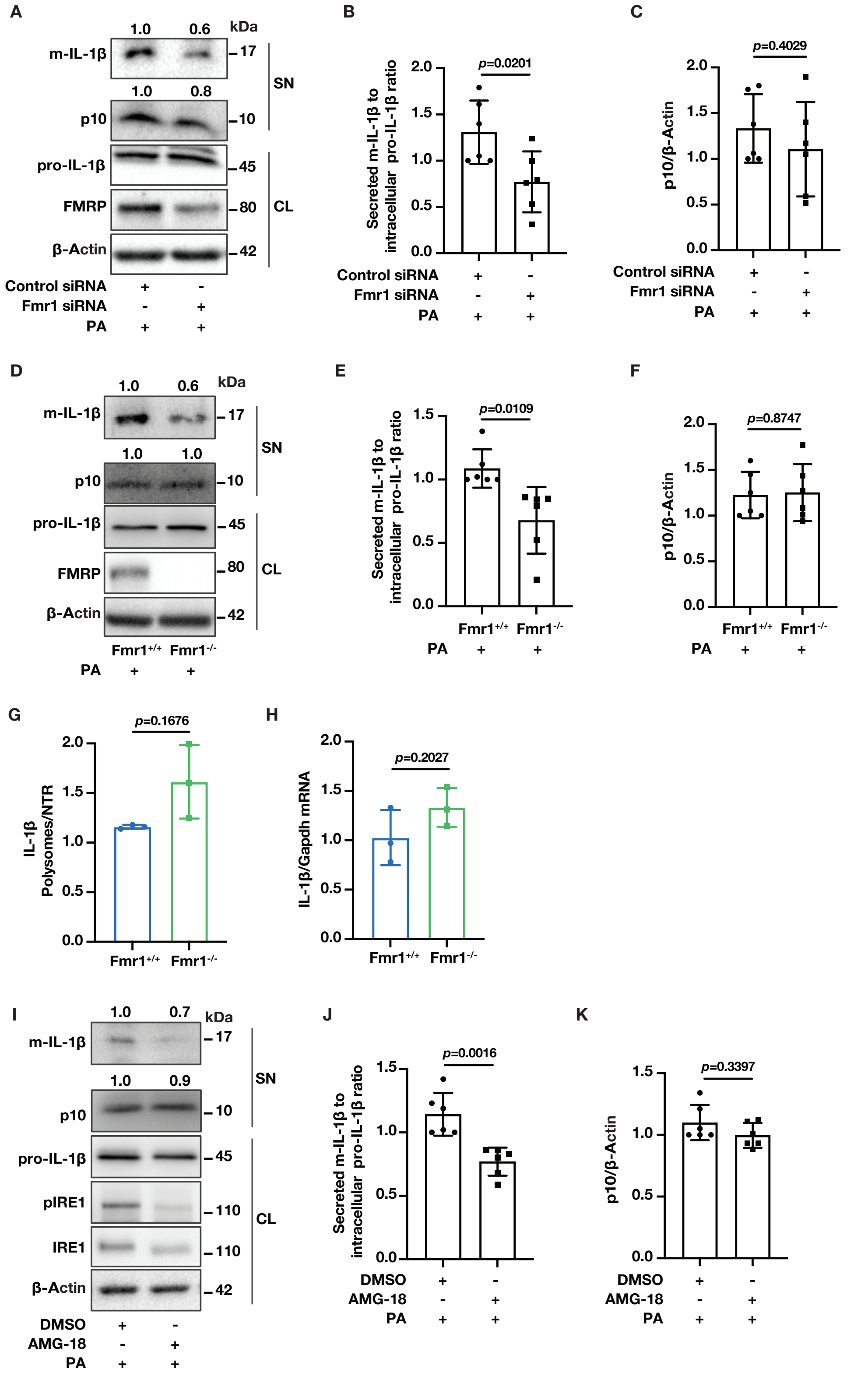

Appendix Figure S6. IRE1 Kinase domain and FMRP regulates IL-1β secretion in macrophages.

A BMDMs were transfected with Fmr1- or control-siRNA and 24 hours after transfection cells were primed with LPS (200 µM) for 3 hours followed by PA (500 µM) treatment for 16 hours (n=6 biological replicates).

B Quantification of secreted m-IL-1β to intracellular pro-IL-1β ratio normalized to β-Actin in Appendix Figure S6A.

C Quantification of secreted p10 (caspase 1) to β-Actin in Appendix Figure S6A.

D $Fmr1^{+/+}$ and $Fmr1^{-/-}$ BMDMs were primed with LPS (200 µM) for 3 hours followed by PA (500 µM) treatment for 16 hours (n=6 biological replicates).

E Quantification of secreted m-IL-1β to intracellular pro-IL-1β ratio normalized to β-Actin in Appendix Figure S6D.

F Quantification of secreted p10 (caspase 1) to β-Actin in Appendix Figure S6D.

G The ratio of the pro-IL-1β mRNA in polysome to NTR fraction (n=3 biological replicates).

H qRT-PCR analysis of pro-IL-1β in total mRNA levels from same samples used in polysome fractions (n=3 biological replicates).

I BMDM cells were pre-treated with AMG-18 (10 µM) for 1 hour and the primed with LPS (200 µM) for 3 hours followed by PA (500 µM) treatment for 16 hours (n=6 biological replicates).

J Quantification of secreted m-IL-1β to intracellular pro-IL-1β ratio normalized to β-Actin in Appendix Figure S6I.

K Quantification of secreted p10 (caspase 1) to β-Actin in Appendix Figure S6I.

Data information: A representative blot is shown. In A and I data are cumulative results of 2 independent experiments. In D data are cumulative results of 3 independent experiments. Supernatants were analyzed by western blotting using specific antibody for IL-1β and caspase-1 and protein lysates were analyzed by western blotting using specific antibodies for FMRP, pIRE1, IRE1 and β-Actin and fold inductions are depicted above the blots. Data are mean ± SEM. Unpaired *t*-test with Welch's correction.
