# Supplementary figures and images for: Intercepting IRE1 kinase‐FMRP signaling prevents atherosclerosis progression
Source: EMBO Mol Med. 2022 Feb 22;14(4):e15344. doi: 10.15252/emmm.202115344 (PMC8988208; doi:10.15252/emmm.202115344)

Fig.1B

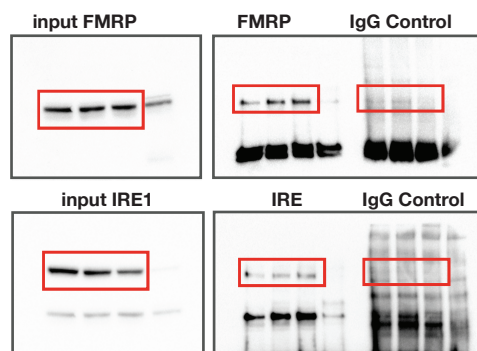

Fig.1C

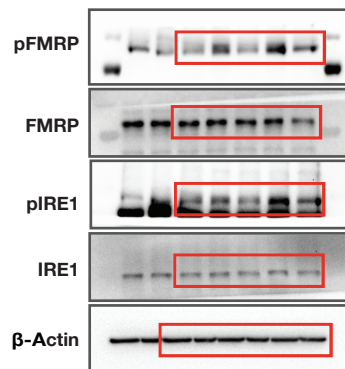

Fig.1D

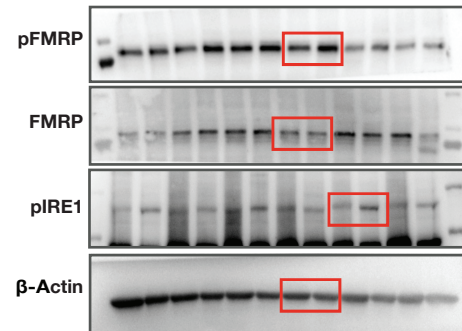

Fig.1 Source data

Fig.1G

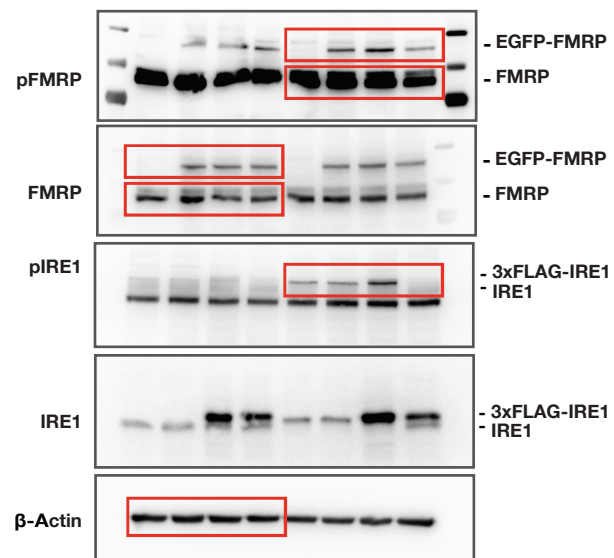

Fig.1F

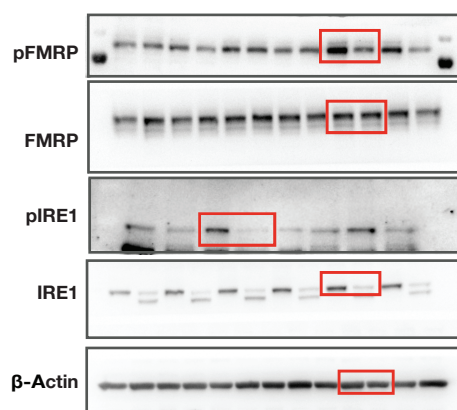

Fig.1E

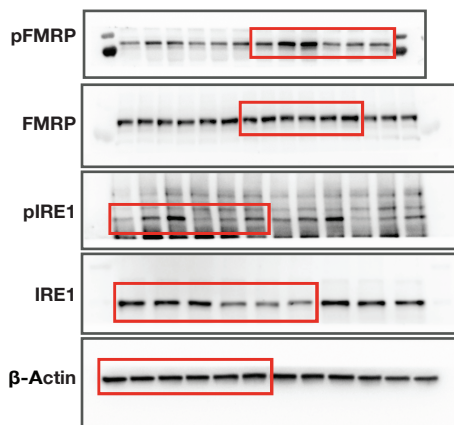

Fig.1H

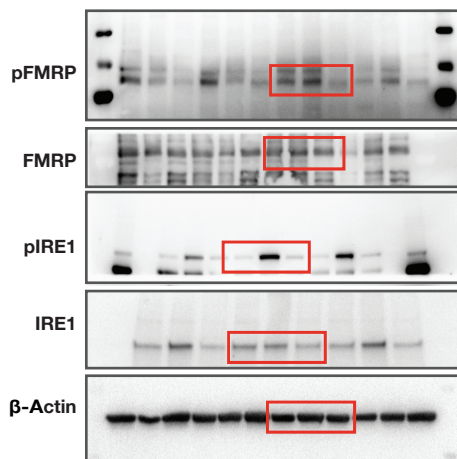

Fig.1I

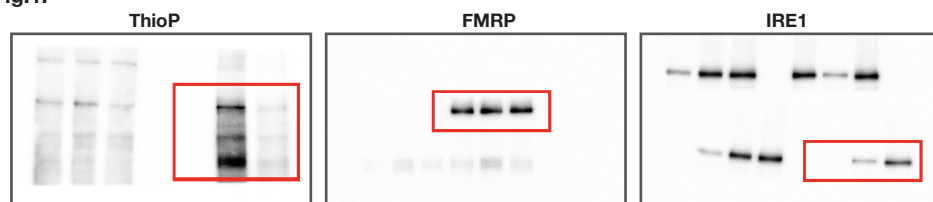

Fig.1J

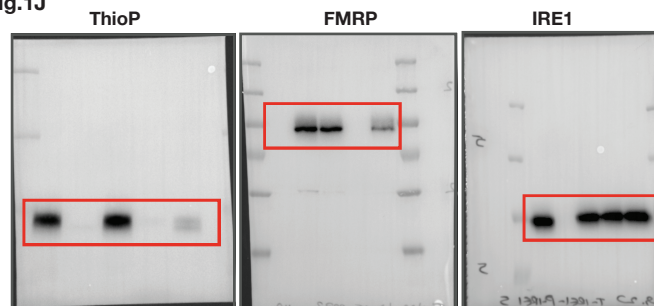

Fig.1K

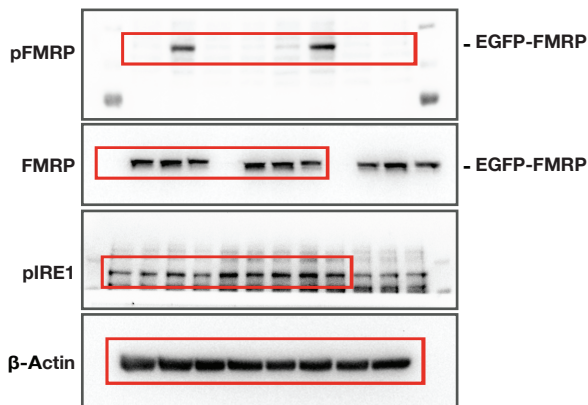

Supplement: Supplementary file 3 — Source Data for Figure 1 [file EMMM-14-e15344-s005.pdf]

Fig.1C

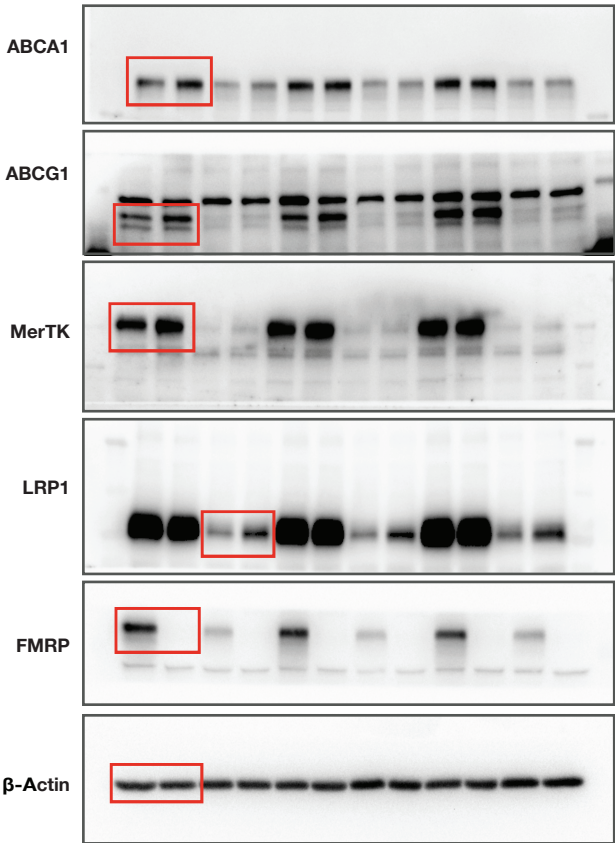

Fig.1D

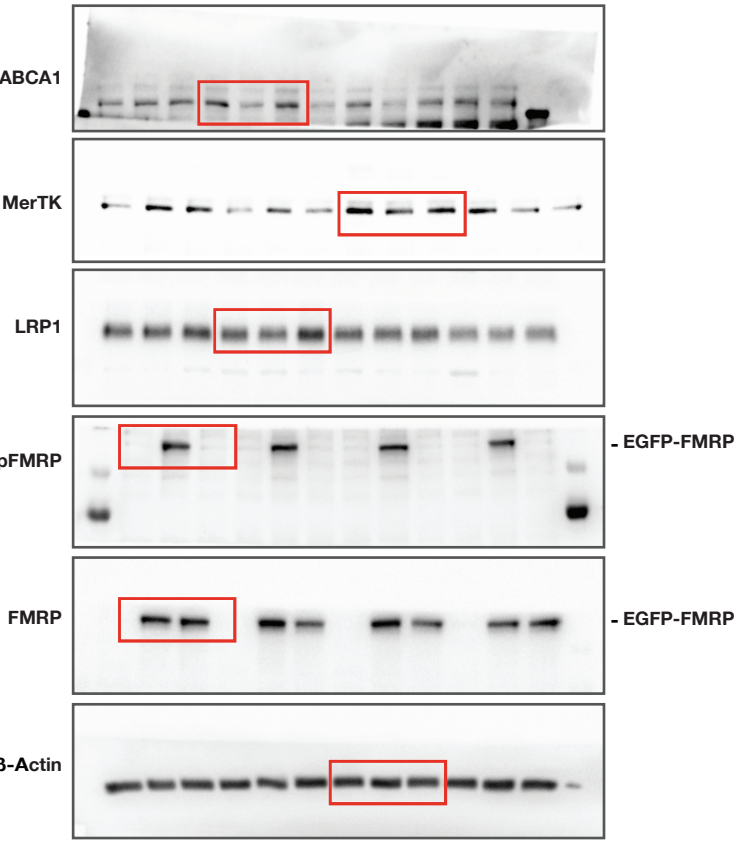

Supplement: Supplementary file 6 — Source Data for Figure 4 [file EMMM-14-e15344-s006.zip › EMM-2021-15344_SourceDataForFigure4.pdf]
