# Supplementary material for: Intercepting IRE1 kinase‐FMRP signaling prevents atherosclerosis progression
Source: EMBO Mol Med. 2022 Feb 22;14(4):e15344. doi: 10.15252/emmm.202115344 (PMC8988208; doi:10.15252/emmm.202115344)
Supplement: Supplementary file 9 [file EMMM-14-e15344-s003.pdf]

EMBO Molecular Medicine

Intercepting IRE1 Kinase-FMRP Signaling Prevents Atherosclerosis Progression

Zehra Yildirim, Sabyasachi Baboo, Syed Muhammad Hamid, Asli Dogan, Ozlem Tufanli, Sabrina Robichaud, Christina Emerton, Jolene Diedrich, Hasan Vatandaslar, Fotis Nikolos, Yanghong Gu, Takao Iwawaki, Elizabeth Tarling, Mireille Ouimet, David Nelson, John Yates III, Peter Walter, and Ebru Erbay
DOI: 10.15252/emmm.202115344

Corresponding author: Ebru Erbay (ebru.erbay@cshs.org)

Review Timeline:

Submission Date:	27th Oct 21
Editorial Decision:	30th Nov 21
Revision Received:	3rd Jan 22
Editorial Decision:	27th Jan 22
Revision Received:	2nd Feb 22
Accepted:	4th Feb 22

Editor: Lise Roth

Transaction Report:

(Note: With the exception of the correction of typographical or spelling errors that could be a source of ambiguity, letters and reports are not edited. Depending on transfer agreements, referee reports obtained elsewhere may or may not be included in this compilation. Referee reports are anonymous unless the Referee chooses to sign their reports.)

30th Nov 2021

Dear Dr. Erbay,

Thank you for the submission of your manuscript to EMBO Molecular Medicine. We have now received feedback from two of the three reviewers who agreed to evaluate your manuscript. Given that referee #1 has not yet returned his/her report despite several chasers, and that both referees #2 and #3 provide similar recommendations, we prefer to make a decision now in order to avoid further delay. Should referee #1 provide a report, we will send it to you, with the understanding that we will not ask you extensive experiments in addition to the ones required in the enclosed reports from referee #2 and #3.

As you will see from the reports below, the referees acknowledge the interest of the study and are supporting publication of your work pending appropriate revisions.

Addressing the reviewers' concerns in full will be necessary for further considering the manuscript in our journal, and acceptance of the manuscript will entail a second round of review. EMBO Molecular Medicine encourages a single round of revision only and therefore, acceptance or rejection of the manuscript will depend on the completeness of your responses included in the next, final version of the manuscript. For this reason, and to save you from any frustrations in the end, I would strongly advise against returning an incomplete revision.

Revised manuscripts should be submitted within three months of a request for revision; they will otherwise be treated as new submissions, except under exceptional circumstances in which a short extension is obtained from the editor.

When submitting your revised manuscript, please carefully review the instructions that follow below. We perform an initial quality control of all revised manuscripts before re-review; failure to include requested items will delay the evaluation of your revision.

We require:

1) A .docx formatted version of the manuscript text (including legends for main figures, EV figures and tables). Please make sure that the changes are highlighted to be clearly visible.

2) Individual production quality figure files as .eps, .tif, .jpg (one file per figure). For guidance, download the 'Figure Guide PDF' (https://www.embopress.org/page/journal/17574684/authorguide#figureformat).

3) A .docx formatted letter INCLUDING the reviewers' reports and your detailed point-by-point responses to their comments. As part of the EMBO Press transparent editorial process, the point-by-point response is part of the Review Process File (RPF), which will be published alongside your paper.

4) A complete author checklist, which you can download from our author guidelines (https://www.embopress.org/page/journal/17574684/authorguide#submissionofrevisions). Please insert information in the checklist that is also reflected in the manuscript. The completed author checklist will also be part of the RPF.

5) For data quantification: please specify the name of the statistical test used to generate error bars and P values, the number (n) of independent experiments (specify technical or biological replicates) underlying each data point and the test used to calculate p-values in each figure legend. The figure legends should contain a basic description of n, P and the test applied. Graphs must include a description of the bars and the error bars (s.d., s.e.m.).

6) We would also encourage you to include the source data for figure panels that show essential data. Numerical data should be provided as individual .xls or .csv files (including a tab describing the data). For blots or microscopy, uncropped images should be submitted (using a zip archive if multiple images need to be supplied for one panel). Additional information on source data and instruction on how to label the files are available at
.

7) Our journal encourages inclusion of *data citations in the reference list* to directly cite datasets that were re-used and obtained from public databases. Data citations in the article text are distinct from normal bibliographical citations and should directly link to the database records from which the data can be accessed. In the main text, data citations are formatted as follows: "Data ref: Smith et al, 2001" or "Data ref: NCBI Sequence Read Archive PRJNA342805, 2017". In the Reference list, data citations must be labeled with "[DATASET]". A data reference must provide the database name, accession number/identifiers and a resolvable link to the landing page from which the data can be accessed at the end of the reference. Further instructions are available at .

8) We replaced Supplementary Information with Expanded View (EV) Figures and Tables that are collapsible/expandable online.

A maximum of 5 EV Figures can be typeset. EV Figures should be cited as 'Figure EV1, Figure EV2" etc... in the text and their respective legends should be included in the main text after the legends of regular figures.
- For the figures that you do NOT wish to display as Expanded View figures, they should be bundled together with their legends in a single PDF file called *Appendix*, which should start with a short Table of Content. Appendix figures should be referred to in the main text as: "Appendix Figure S1, Appendix Figure S2" etc.
- Additional Tables/Datasets should be labeled and referred to as Table EV1, Dataset EV1, etc. Legends have to be provided in a separate tab in case of .xls files. Alternatively, the legend can be supplied as a separate text file (README) and zipped together with the Table/Dataset file.
See detailed instructions here:
.

9) For more information: There is space at the end of each article to list relevant web links for further consultation by our readers. Could you identify some relevant ones and provide such information as well? Some examples are patient associations, relevant databases, OMIM/proteins/genes links, author's websites, etc...

10) Every published paper now includes a 'Synopsis' to further enhance discoverability. Synopses are displayed on the journal webpage and are freely accessible to all readers. They include a short stand first (maximum of 300 characters, including space) as well as 2-5 one-sentences bullet points that summarizes the paper. Please write the bullet points to summarize the key NEW findings. They should be designed to be complementary to the abstract - i.e. not repeat the same text. We encourage inclusion of key acronyms and quantitative information (maximum of 30 words / bullet point). Please use the passive voice. Please attach these in a separate file or send them by email, we will incorporate them accordingly.

Please also suggest a striking image or visual abstract to illustrate your article as a PNG file 550 px wide x 300-600 px high.

11) As part of the EMBO Publications transparent editorial process initiative (see our Editorial at http://embomolmed.embopress.org/content/2/9/329), EMBO Molecular Medicine will publish online a Review Process File (RPF) to accompany accepted manuscripts.
In the event of acceptance, this file will be published in conjunction with your paper and will include the anonymous referee reports, your point-by-point response and all pertinent correspondence relating to the manuscript. Let us know whether you agree with the publication of the RPF and as here, if you want to remove or not any figures from it prior to publication.
Please note that the Authors checklist will be published at the end of the RPF.

EMBO Molecular Medicine has a "scooping protection" policy, whereby similar findings that are published by others during review or revision are not a criterion for rejection. Should you decide to submit a revised version, I do ask that you get in touch after three months if you have not completed it, to update us on the status.

I look forward to receiving your revised manuscript.

Yours sincerely,

Lise Roth

Lise Roth, PhD
Editor
EMBO Molecular Medicine

***** Reviewer's comments *****

Referee #2 (Comments on Novelty/Model System for Author):

The authors present a clear and systematic rationalization and evaluation of the role of FMRP in macrophages and in atherosclerosis development.

Referee #2 (Remarks for Author):

In this manuscript, Yildirim and colleagues identify a novel function of FMRP protein in macrophages, which is well-known for its crucial in normal brain development. The authors present clear data showing that the ER stress sensor IRE-1 phosphorylates FMRP protein, which results in suppression of macrophage cholesterol efflux and apoptotic cell clearance. As FMRP is an RNA binding protein, the authors show that FMRP phosphorylation enhances the translational suppression of major cholesterol transporters and efferocytosis receptors, including ABCA1, ABCG1, MERTK and LRP1. Furthermore, using established mouse models of atherosclerosis, the authors show that FMRP deficiency or systemic IRE-1 kinase inhibition lowers en face aortic lesion development and inhibits necrotic core formation in the aortic roots. The results in this study are of good quality and in the interest of the field as ER stress contributes many aspects of obesity-induced metabolic perturbations, including atherosclerosis. The authors present strong data to support their proposed mechanism, and some aspects of the manuscript could be improved by considering some suggestions listed below.

1- Previous work from the same group has shown that inhibiting IRE1's both kinase and endoribonuclease activities in macrophages suppresses hyperlipidemia-induced IL-1β and IL-18 production, which contributes to the beneficial effect of IRE-1 inhibition in lowering atherosclerosis formation in ApoE-/- mice. Have the authors examined the levels of IL-1β and/or IL-18 in FMRP-/- or AMG-18 treated macrophages?
2- The authors show lower TUNEL-positivity and plaque necrosis in both FMRP-deficient and AMG-18 treated ApoE-/- mice. As lower TUNEL positivity in lesions reflects both enhanced efferocytosis and apoptosis suppression, have the authors attempted to measure whether ER stress-induced macrophage apoptosis is decreased in FMRP-/- and/or AMG-18-treated macrophages ex vivo?
3- Can the authors show or discuss whether FMRP is the only mediator downstream of IRE1 in regulating macrophage cholesterol efflux and/or apoptotic cell clearance?

Referee #3 (Comments on Novelty/Model System for Author):

The current study investigates the role of FMRP in cholesterol efflux and efferocytosis both in cell culture experiments and in vivo models in mice, including an atherosclerosis model. This is largely an unexplored area, and the investigators use a variety of experimental approaches to support their conclusions, which strengthens the study considerably.

Referee #3 (Remarks for Author):

The current study investigates the role of FMRP in cholesterol efflux, efferocytosis both in cell culture experiments and in vivo models in mice. The results demonstrate that ER stress-induced activation of IRE1 leads to FMRP phosphorylation and suppression of macrophage cholesterol efflux and efferocytosis. Further, the study demonstrates that FMRP-deficiency and pharmacological inhibition of IRE1 kinase activity enhances cholesterol efflux and efferocytosis. The final experiments examine FMRP-deficiency (global) as well as in myeloid cells as well as IRE1 inhibition on the progression of atherosclerosis in various mouse models.

Overall, this is an important study that is carefully done with numerous experiments to confirm that largely confirm the conclusions reached by the authors. A few items need to be addressed that would strengthen the paper:

1. There is some concern about certain aspects of data analysis. For example, it's not clear how the immunoblot data which is prevalent throughout the study were analyzed. In almost all cases it appears the control was assigned a value of 1.0. As an example, the data in S1F, H, and I, the IRE+/+ results (pFMRP/FMRP) are always set to 1.0. Obviously, there is variation in these samples, which would impact the significance. The same problem is true for the data in S3C and S3D.

2. It does not look like very many residential macrophages in the control group (DMSO) are oil-red O positive in Fig 2B.

3. In Figs 2C,D the authors refer to "cholesterol accumulation" and Fig 2E refer to "reduced foam cell formation". The experiments are measuring %dil-acLDL internalized; I would recommend that they refer to the actual event they are measuring to be accurate in their descriptions.

4. Based on the results of experiments in Fig 2C,D, on page 6, line 217 the authors state that "We reasoned that this observation could be related to an increase in cholesterol export (RCT; due to increased translation of cholesterol exporters) from Fmr1-/- macrophages." While this appears to be the case, the measured data show decreased levels of dil-ac-LDL in cells. This could also be impacted by reduction in receptors involved in uptake as well. The authors might consider mentioning this possibility.

5. Fig 4D, differences between EV WT or STSA are extremely small. Once again, there is a question about data analysis. Were

the data normalized to EV (all EV values are 1.0). Variations in EV would certainly alter statistical analysis.

Minor suggestions:
1. On Page 4, line 58 "Endogenous FMRP and IRE1, migrated faster than EGFP-FMRP and FLAG-IRE1, respectively." Clarify - migrated faster upon SDS-PAGE?

2. Page 5, line 207 "Next, we fed Apoe-/- mice with a WD (12 weeks) and injected them daily? with the IRE1 kinase inhibitor" Not sure why ? is present

Referee #2 (Comments on Novelty/Model System for Author):

The authors present a clear and systematic rationalization and evaluation of the role of FMRP in macrophages and in atherosclerosis development.

Referee #2 (Remarks for Author):

In this manuscript, Yildirim and colleagues identify a novel function of FMRP protein in macrophages, which is well-known for its crucial in normal brain development. The authors present clear data showing that the ER stress sensor IRE-1 phosphorylates FMRP protein, which results in suppression of macrophage cholesterol efflux and apoptotic cell clearance. As FMRP is an RNA binding protein, the authors show that FMRP phosphorylation enhances the translational suppression of major cholesterol transporters and efferocytosis receptors, including ABCA1, ABCG1, MERTK and LRP1. Furthermore, using established mouse models of atherosclerosis, the authors show that FMRP deficiency or systemic IRE-1 kinase inhibition lowers en face aortic lesion development and inhibits necrotic core formation in the aortic roots. The results in this study are of good quality and in the interest of the field as ER stress contributes many aspects of obesity-induced metabolic perturbations, including atherosclerosis. The authors present strong data to support their proposed mechanism, and some aspects of the manuscript could be improved by considering some suggestions listed below.

Response: We thank the reviewer for recognizing the novelty of our findings and the good quality of our data. We appreciate these encouraging remarks and the reviewer's constructive feedback, which we aspired to build upon in our revised manuscript.

Question 1: Previous work from the same group has shown that inhibiting IRE1's both kinase and endoribonuclease activities in macrophages suppresses hyperlipidemia-induced IL-1β and IL-18 production, which contributes to the beneficial effect of IRE-1 inhibition in lowering atherosclerosis formation in ApoE-/- mice. Have the authors examined the levels of IL-1β and/or IL-18 in FMRP-/- or AMG-18 treated macrophages?

Response 1: We thank the reviewer for pointing out our group's prior published findings that described IRE1's endoribonuclease activity inhibition contributes to both hyperlipidemia-induced inflammation and atherosclerosis formation. In this study, for the first time, we attempted to investigate IRE1's kinase activity and novel substrate, FMRP's role in atherosclerosis. Indeed, hypercholesterolemia and inflammation are

intertwined and both drive atherogenesis. As suggested by the reviewer, we investigated the impact of FMRP deficiency on lipid-induced inflammation, specifically IL-1β, in macrophages. As seen below (A and B), FMRP loss of function (in both Fmr1 knock down that were transfected with Fmr1-specific siRNA and FMRP knock out bone marrow-derived mouse macrophages (BMDM)), abrogated lipid-induced m-IL-1β secretion. However, knocking out FMRP had no impact on Fmr1 mRNA associated with the polysomes or in the whole cell lysates (C and D). FMRP loss of function also did not appear to alter lipid-induced inflammasome activation as assessed by active caspase-1 in the conditioned medium of these macrophages (A and B).

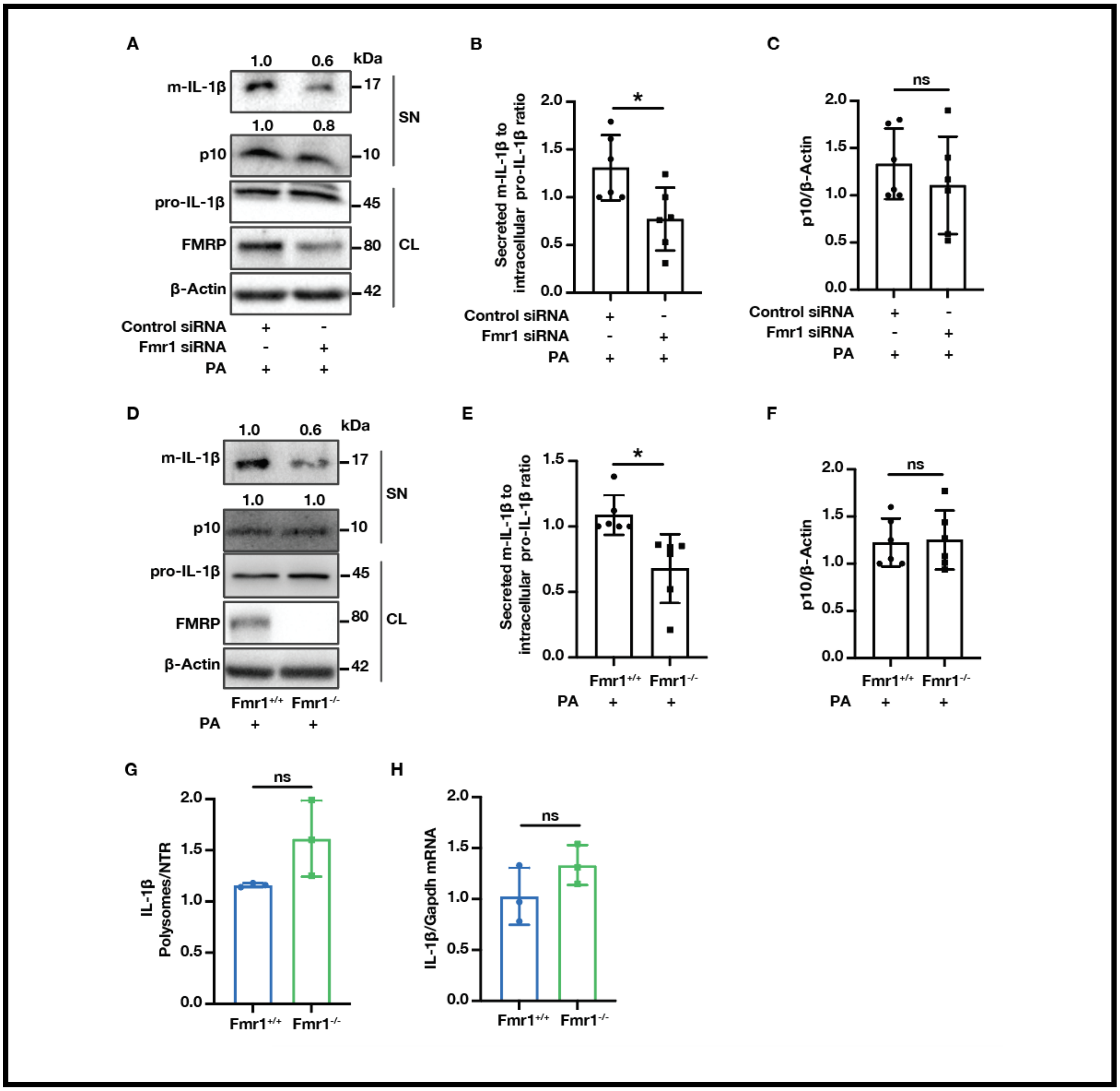

Appendix Figure S6. **IRE1 Kinase domain and FMRP regulates IL-1β secretion in macrophages.**

(A) BMDMs were transfected with Fmr1- or control-siRNA and 24 hours after transfection cells were primed with LPS (200 µM) for 3 hours followed by PA (500 µM) treatment for 16 hours (n=6). (B) Quantification of secreted m-IL-1β to intracellular pro-IL-1β ratio normalized to β-Actin in Appendix Figure S6A. (C) Quantification of secreted p10 (caspase 1) to β-Actin in Appendix Figure S6A. (D) $Fmr1^{+/+}$ and $Fmr1^{-/-}$ BMDMs were primed with LPS (200 µM) for 3 hours followed by PA (500 µM) treatment for 16 hours (n=6). (E) Quantification of secreted m-IL-1β to intracellular pro-IL-1β ratio normalized to β-Actin in Appendix Figure S6D. (F) Quantification of secreted p10 (caspase 1) to β-Actin in Appendix Figure S6D. (G) The ratio of the pro-IL-1β mRNA in polysome to NTR fraction (n=3). **(H)** qRT-PCR analysis of pro-IL-1β in total mRNA levels from same samples used in polysome fractions (n=3). Supernatants were analyzed by western blotting using specific antibody for IL-1β and caspase-1 and protein lysates were analyzed by western blotting using specific antibodies for FMRP, pIRE1, IRE1 and β-Actin.

As suggested by the reviewer, we also treated bone marrow derived mouse macrophages (BMDM) with AMG-18, which reduced lipid-induced mature IL-1β (m-IL-1β) secretion (seen in E).

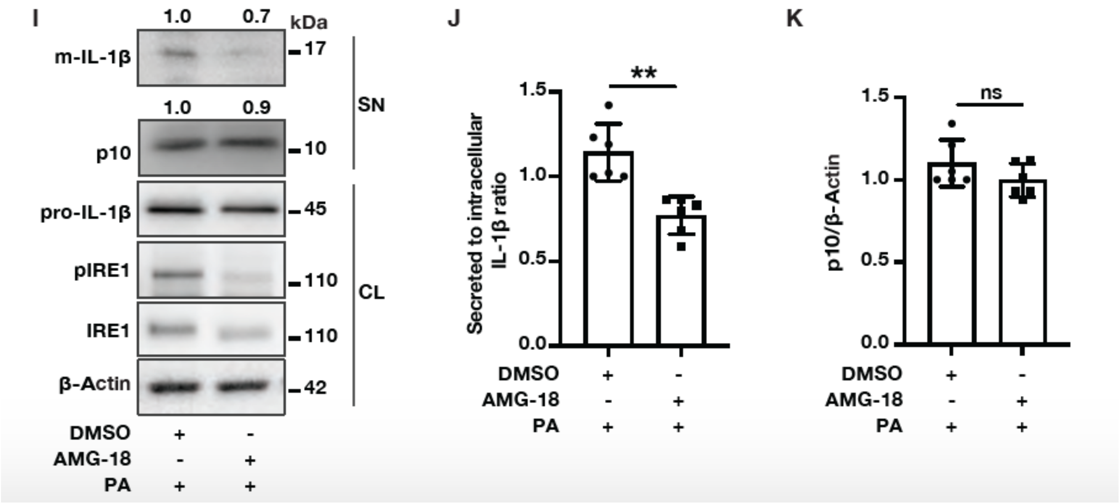

Appendix Figure S6. **IRE1 Kinase domain and FMRP regulates IL-1β secretion in macrophages.**

(I) BMDM cells were pre-treated with AMG-18 (10 µM) for 1 hour and the primed with LPS (200 µM) for 3 hours followed by PA (500 µM) treatment for 16 hours (n=6). (J) Quantification of secreted m-IL-1β to intracellular pro-IL-1β ratio normalized to β-Actin in Appendix Figure S6I. (K) Quantification of secreted p10 (caspase 1) to β-Actin in Appendix Figure S6I. Supernatants were analyzed by western blotting using specific antibody for IL-1β and caspase-1 and protein lysates were analyzed by western blotting using specific antibodies for FMRP, pIRE1, IRE1 and β-Actin.

Finally, we added the following in to our discussion while referring to the above figures as the new Appendix Fig S6A-S6K: "*In an earlier study, we had shown that small molecule inhibitors that are specific for IRE1's RNase activity prevented lipid-induced inflammasome activation and secretion of mature interleukin- 1β (mIL-1β) and mIL-18 in both mouse and human macrophages while reducing hyperlipidemia-induced m-IL-1β and m-IL-18 production and atherosclerotic plaque size in mice (Tufanli et al., 2017). FMRP suppression also reduces mIL-1β secreted from macrophages but without altering inflammasome activation (Appendix Fig S6A-S6F). Knocking out FMRP from macrophages has no effect on IL-1β mRNA levels in the whole cell lysate or in the polysomes, suggesting against transcriptional or translational control over these cytokines' production (Appendix Fig S6G and S6H). As expected, inhibition of IRE1 kinase also reduces mIL-1β (Appendix Fig S6I-S6K). Intriguingly, IL-1β can be secreted through ABCA1 and compete with cholesterol efflux through the same transporter (Tumurkhuu et al, 2018). Since the inhibition of IRE1 kinase-FMRP axis leads to a marked upregulation of cholesterol efflux in macrophages (as shown in our study), it is plausible that increased demand for ABCA1 for cholesterol efflux could prevent IL-1□ secretion through this route.*"

<u>Question 2:</u> The authors show lower TUNEL-positivity and plaque necrosis in both FMRP-deficient and AMG-18 treated ApoE-/- mice. As lower TUNEL positivity in lesions reflects both enhanced efferocytosis and apoptosis suppression, have the authors attempted to measure whether ER stress-induced macrophage apoptosis is decreased in FMRP-/- and/or AMG-18-treated macrophages ex vivo?

<u>Response 2:</u> We share the concern for this possibility with the reviewer and as suggested by the reviewer, we assessed apoptosis in macrophages. As seen below, we observed that both FMRP genetic deletion and IRE1 kinase inhibition did not alter apoptosis (as measured by PI positive cells using flow cytometry). This finding supports the notion that enhanced efferocytosis is the primary consequence of inhibiting IRE1-FMRP pathway. The below graph (as Appendix Figure S4I) and the relevant results discussion was added to our revised manuscript: "*We further investigated whether apoptosis is altered in* $Fmr1^{-/-}$ *and AMG-18 treated macrophages. There was no significant change between the groups (Appendix Fig 4I), supporting the notion that the primary consequence of inhibiting IRE1-FMRP signaling is efficient clearance of apoptotic cells through increasing efferocytosis capacity.*"

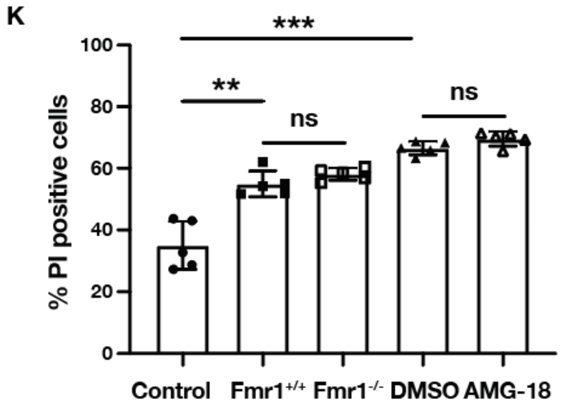

Appendix Figure S4. (I) Fmr1$^{+/+}$ and Fmr1$^{-/-}$ BMDMs or AMG-18 pre-treated cells (10 µM, 1 hour) were treated with PA (500 µM) treatment for 12 hours and then stained with Propidium iodide (PI) (n=5). Data are mean ± SEM. Unpaired t-test with Welch's correction. *P≤ 0.05, **P≤ 0.01, ***P≤ 0.001.

Question 3: Can the authors show or discuss whether FMRP is the only mediator downstream of IRE1 in regulating macrophage cholesterol efflux and/or apoptotic cell clearance?

Response 3: We added these points to our discussion in the revised manuscript: *"Although we cannot definitively show that FMRP is the only mediator downstream of IRE1 kinase activity in regulating the proposed mechanisms, our data strongly supports that this novel IRE1 kinase target's expression in macrophages plays an important role in cholesterol efflux and apoptotic cell clearance and is an important contributor to atheroprotection offered by IRE1 kinase inhibition. Further investigation is required to uncover other potential IRE1 kinase substrates and their roles in macrophages and atherosclerosis".*

Referee #3 (Comments on Novelty/Model System for Author):

The current study investigates the role of FMRP in cholesterol efflux and efferocytosis both in cell culture experiments and in vivo models in mice, including an atherosclerosis model. This is largely an unexplored area, and the investigators use a variety of experimental approaches to support their conclusions, which strengthens the study considerably.

Referee #3 (Remarks for Author):

The current study investigates the role of FMRP in cholesterol efflux, efferocytosis both in cell culture experiments and in vivo models in mice. The results demonstrate that ER stress-induced activation of IRE1 leads to FMRP phosphorylation and suppression of macrophage cholesterol efflux and efferocytosis. Further, the study demonstrates that FMRP-deficiency and pharmacological inhibition of IRE1 kinase activity enhances cholesterol efflux and efferocytosis. The final experiments examine FMRP-deficiency (global) as well as in myeloid cells as well as IRE1 inhibition on the progression of atherosclerosis in various mouse models.

Overall, this is an important study that is carefully done with numerous experiments to confirm that largely confirm the conclusions reached by the authors. A few items need to be addressed that would strengthen the paper:

Response: We thank the reviewer for his/her enthusiastic evaluation of our study's novel findings and for the reviewer's constructive feedback that encouraged us to improve our manuscript.

Question 1: There is some concern about certain aspects of data analysis. For example, it's not clear how the immunoblot data which is prevalent throughout the study were analyzed. In almost all cases it appears the control was assigned a value of 1.0. As an example, the data in S1F, H, and I, the IRE+/+ results (pFMRP/FMRP) are always set to 1.0. Obviously, there is variation in these samples, which would impact the significance. The same problem is true for the data in S3C and S3D.

Response 1: We thank the reviewer for giving us an opportunity to clarify how the western blot data analysis was performed. In order to assess the reviewer's concern, we updated our quantifications (specifically for figures with quantified blots such as (Figure 1C, 1D, 1E, 1F, 1G, 1H, Figure 4C, 4D) by assigning only one control to 1.0 per gel/blot (if multiple controls were run on the same gel) and calculated relative fold change for the rest of the controls and samples run on the same gel/blot. This standard approach takes into account that our experimental sets were **(a)** performed on different dates and could be impacted by variations in cell numbers and cell cycle in the absence of synchronization, or **(b)** run on gels and blotted/developed on different days where band signal intensity and background may have been influenced by antibody affinity/concentration or developing reagent/conditions on different days. The quantification methods and statistical test results are provided as source data files for each graph throughout the paper. Below is the detailed explanation for fold change calculation and a sample calculation for Figure 1D.

We used a standard approach utilized by many labs to analyze the changes on FMRP phosphorylation across multiple experimental set ups (plated, treated, lysed on different days) or gels/blots run/developed on different days. We first determined the **ratio for P-FMRP/ FMRP band intensity** for all controls and samples. We set the value for **the control with the lowest ratio as "1.0"** and determined the **fold change for the other controls and treatment samples on the same gel/blot**. If there were additional experimental sets run on different gels, we did the same calculation for each gel/blot. After this we combined all the experimental sets by first determining **the average of the control samples in all sets/gels/blots and assigning this average with the value of 1.0.** Then, we took the average (of the fold change) for a certain treatment group determined the average mean value for the fold change.

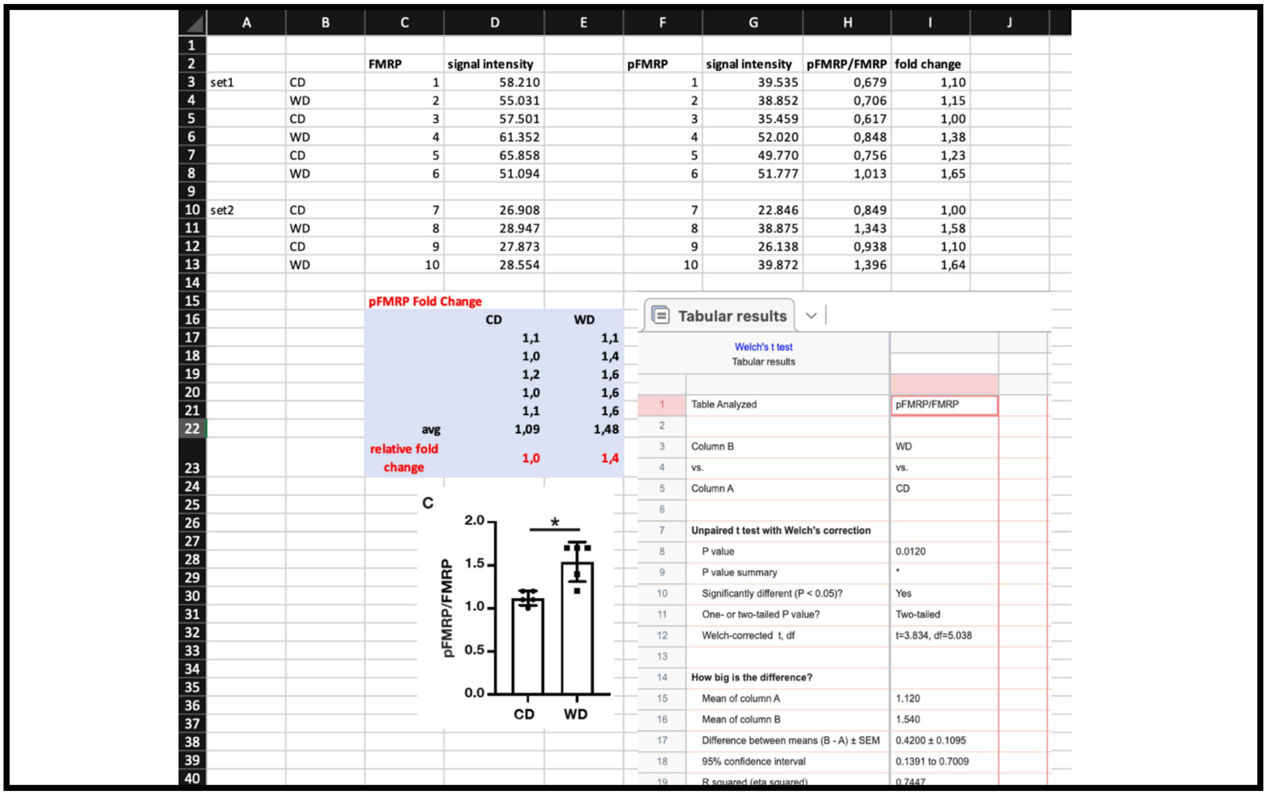

	A	B	C	D	E	F	G	H	I
2			FMRP	signal intensity		pFMRP	signal intensity	pFMRP/FMRP	fold change
3	set1	CD	1	58.210		1	39.535	0,679	1,10
4		WD	2	55.031		2	38.852	0,706	1,15
5		CD	3	57.501		3	35.459	0,617	1,00
6		WD	4	61.352		4	52.020	0,848	1,38
7		CD	5	65.858		5	49.770	0,756	1,23
8		WD	6	51.094		6	51.777	1,013	1,65
10	set2	CD	7	26.908		7	22.846	0,849	1,00
11		WD	8	28.947		8	38.875	1,343	1,58
12		CD	9	27.873		9	26.138	0,938	1,10
13		WD	10	28.554		10	39.872	1,396	1,64

pFMRP Fold Change		CD	WD
		1,1	1,1
		1,0	1,4
		1,2	1,6
		1,0	1,6
		1,1	1,6
	avg	1,09	1,48
relative fold change		1,0	1,4

Tabular results

Welch's t test Tabular results		
1	Table Analyzed	pFMRP/FMRP
2		
3	Column B	WD
4	vs.	vs.
5	Column A	CD
6		
7	Unpaired t test with Welch's correction	
8	P value	0.0120
9	P value summary	*
10	Significantly different (P < 0.05)?	Yes
11	One- or two-tailed P value?	Two-tailed
12	Welch-corrected t, df	t=3.834, df=5.038
13		
14	How big is the difference?	
15	Mean of column A	1.120
16	Mean of column B	1.540
17	Difference between means (B - A) ± SEM	0.4200 ± 0.1095
18	95% confidence interval	0.1391 to 0.7009
19	R squared (eta squared)	0.7447

One exception to this calculation was Figure 1C (that was quantified in Appendix Figure S1A): In this figure, each experimental set with one control/set was already run on separate gels and developed as separate blots. Here, each gel/blot had a single control sample per gel/blot and each time the control was set at 1.0 value (therefore, all controls are still value 1.0) and treatments were calculated as fold change within each gel/blot.

<u>Question 2.</u> It does not look like very many residential macrophages in the control group (DMSO) are oil-red O positive in Fig 2B.

<u>Response 2:</u> We agree with the reviewer and replaced the figures for both groups with higher quality and better representative images as shown below and in revised Fig.2B.

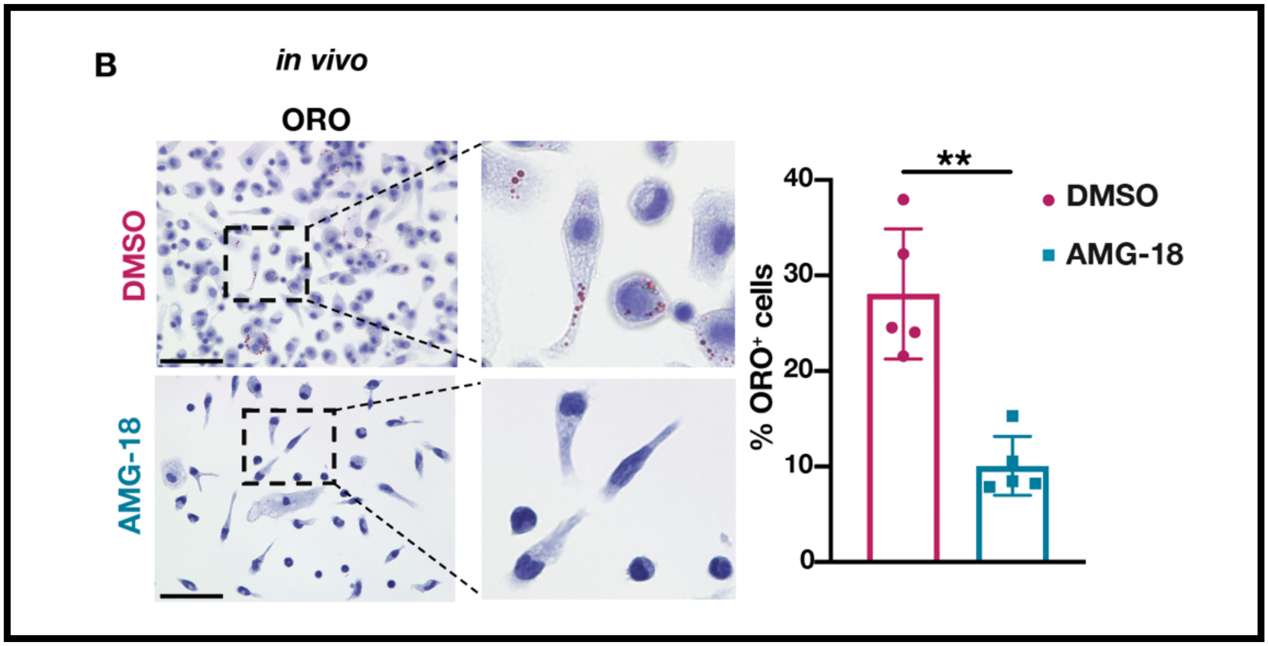

Figure 2. (B) Apoe-/- mice were fed with WD (12 weeks) and injected with vehicle (DMSO) or AMG-18 (30 mg/kg/day) in the last 4 weeks of WD. Residential PM were stained with ORO and imaged (n=5; Scale bar = 50 µm).

<u>Question 3:</u> In Figs 2C, D the authors refer to "cholesterol accumulation" and Fig 2E refer to "reduced foam cell formation". The experiments are measuring % dil-acLDL internalized; I would recommend that they refer to the actual event they are measuring to be accurate in their descriptions.

<u>Response 3:</u> We agree with the reviewer and accordingly, revised our description of the outcome by only referring to the outcome of these experiments in Figures 2C, 2D, and 2E as these treatments *"reduced % dil-acLDL internalized."*

<u>Question 4</u>: Based on the results of experiments in Fig 2C,D, on page 6, line 217 the authors state that "We reasoned that this observation could be related to an increase in cholesterol export (RCT; due to increased translation of cholesterol exporters) from Fmr1-/- macrophages." While this appears to be the case, the

measured data show decreased levels of diI-ac-LDL in cells. This could also be impacted by reduction in receptors involved in uptake as well. The authors might consider mentioning this possibility.

Response 4: We thank the reviewer for this great feedback. As seen in Appendix Figure S2A, neither FMRP knockdown nor IRE1 kinase inhibition alters cholesterol uptake in macrophages. This result supports the conclusions that decreased foam cell formation is due to increased cholesterol export in both FMRP-deficient and IRE1 kinase-inhibited macrophages. We added the below graph to Fig. S2 and explained in the revised manuscript that "*Reduced foam cell formation could be explained with less cholesterol uptake, however, neither FMRP knock down nor IRE1 kinase inhibition altered cholesterol uptake in macrophages (Appendix Fig S2B). We reasoned that this observation is most likely related to an increase in cholesterol export (RCT; due to increased translation of cholesterol exporters) from Fmr1-/- macrophages. Indeed, FMRP deficiency led to an increase in cholesterol efflux coupled to its loading onto the cholesterol carriers, apolipoprotein-A1 (APOA1) and HDL (Fig 2F), and, likewise, the IRE1 kinase inhibitor enhanced cholesterol efflux (Fig 2G). Thus, ER stress-associated reduction in cholesterol efflux is dependent on both IRE1 kinase activity and FMRP.*" in our revised manuscript.

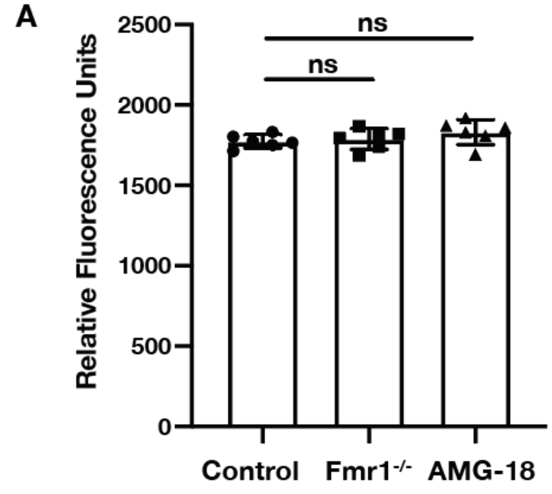

Appendix Figure S2. (A) Fmr1$^{-/-}$ or AMG-18 pre-treated cells (10 µM, 1 hour) were treated with fluorescently labeled cholesterol for 4 hours (n=6). Data are mean ± SEM. Unpaired t-test with Welch's correction. *P≤ 0.05, **P≤ 0.01, ***P≤ 0.001.

Question 5: Fig 4D, differences between EV WT or STSA are extremely small. Once again, there is a

question about data analysis. Were the data normalized to EV (all EV values are 1.0). Variations in EV would certainly alter statistical analysis.

Response 5: Please, see our response to question 1 for a detailed explanation on our data analysis from Western blots. As advised by the reviewer, we updated our calculations for fold change of FMRP target proteins, which occurs in the range of 25-35% when WT_FMRP is compared to STSA_FMRP mutant as seen below (and in the revised Fig.4D and Appendix Fig.S3D):

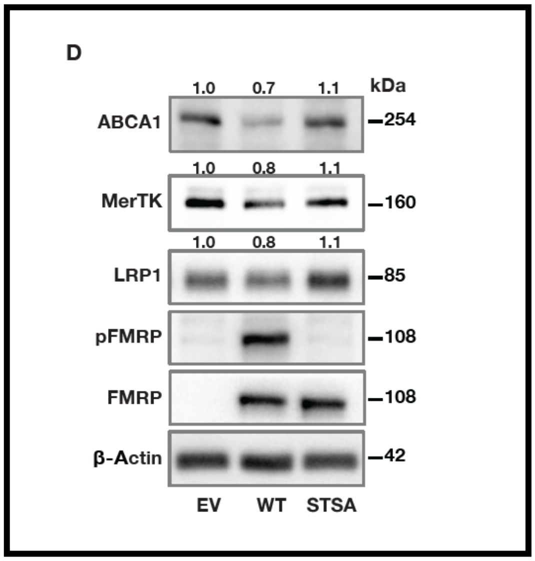

Figure 4. (D) Fmr1-/- MEF cells were transfected with EV, WT-FMRP or STSA-FMRP plasmids followed by PA treatment (500 µM; 6 hours). Protein lysates were analyzed by western blotting using specific antibodies for ABCA1, MerTK, LRP1, pFMRP, FMRP and b-Actin (n=5).

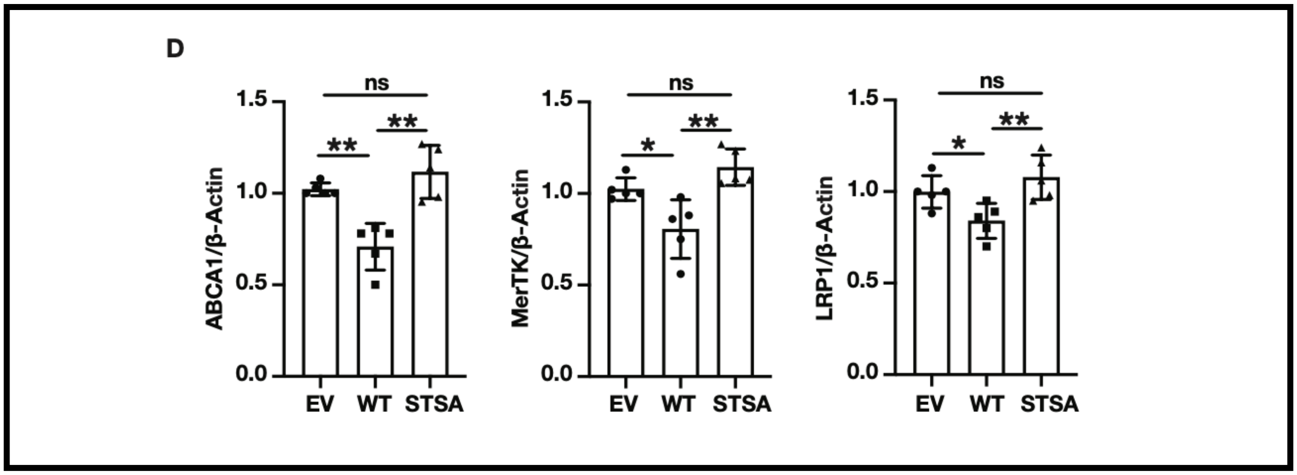

Appendix Figure S3. (D) Western blot quantifications for ABCA1, MerTK and LRP1 in Fig. 4D. The fold change of protein expression level was calculated relative to β-Actin. Data are mean ± SEM. Unpaired t-test with Welch's correction. *P≤ 0.05, **P≤ 0.01, ***P≤ 0.001.

Minor suggestions:

Question 1: On Page 4, line 58 "Endogenous FMRP and IRE1, migrated faster than EGFP-FMRP and FLAG-IRE1, respectively." Clarify - migrated faster upon SDS-PAGE?

Response 1: We revised the description of the experiment as *"Endogenous FMRP and IRE1, migrated faster on SDS-PAGE than epitope tagged EGFP-FMRP and FLAG-IRE1, respectively".*

Question 2: Page 5, line 207 "Next, we fed Apoe-/- mice with a WD (12 weeks) and injected them daily with the IRE1 kinase inhibitor" Not sure why ? is present

Response 2: We thank the reviewer for pointing out this typing mistake. We deleted the question mark (?) in our revised manuscript.

27th Jan 2022

Dear Dr. Erbay,

Thank you for the submission of your revised manuscript to EMBO Molecular Medicine. We have now received the enclosed reports from the referees. As you will see, they are fully supportive of publication, and I am therefore pleased to inform you that we will be able to accept your manuscript once the following editorial points will be addressed:

1/ Main manuscript text:
- Please address the queries from our data editors in track changes mode in the main manuscript file labelled 'Data edited MS file'. Please use this file for any further modification (in track changes mode).
- We can accommodate a maximum of 5 keywords, please adjust accordingly.
- Material and methods:
o Please provide the antibodies dilutions.
o Please indicate the gender of the mice, as well as the housing and husbandry conditions.
o Please indicate whether the cells were authenticated (if applicable) and tested for mycoplasma contamination.
- The 'Data Availability' section should be placed after the Materials and Methods. Kindly note that the Data Availability Section is restricted to new primary datasets generated in this study. (Primary datasets produced in this study need to be deposited in an appropriate public database, and the accession numbers and database listed under 'Data Availability'.) In case you have no data that requires deposition in a public database, please state so in this section.
- Please add funding information in the Acknowledgements section.
- We updated our journal's competing interests policy in January 2022 and request authors to consider both actual and perceived competing interests. Please review the policy https://www.embopress.org/competing-interests and update your competing interests if necessary.

2/ Figures and Appendix:
- Please provide in the figures or in their legends the exact p values, not a range. Some people found that to keep the figures clear, providing a supplemental table in the Appendix with all exact p-values was preferable. You are welcome to do this if you want to.
- Appendix: Merge the Appendix figures in one PDF file, with their legends and table of content. Please note that you have the possibility to have Expanded View (EV) Figures that are collapsible/expandable online. A maximum of 5 EV Figures can be typeset. EV Figures should be cited as 'Figure EV1, Figure EV2" etc... in the text and their respective legends should be included in the main text after the legends of regular figures.
- Thank you for providing Source Data. Please upload them as one PDF file per figure.

3/ Checklist:
- Manuscript number (top left): indicate EMM-2021-15344
- Section F18: if no large dataset was generated in this study (genomic or proteomic study, sequencing, etc), please indicate: This study includes no data deposited in external repositories.

4/ Thank you for providing a synopsis. I slightly shortened the text to fit our style and format, please let me know if you agree with the following:
Targeting IRE1 function and substrate(s) provides a novel therapeutic approach to atherosclerosis. We found a key role for IRE1-mediated FMRP phosphorylation that suppresses the expression of cholesterol transporters and efferocytosis receptors in macrophages and promotes atherosclerosis progression.

• FMRP is a novel IRE1 kinase substrate.

• Lipid-induced, IRE1-mediated FMRP phosphorylation leads to post-transcriptional suppression of cholesterol transporters and efferocytosis regulators.

• Ablation of IRE1 kinase activity or suppression of FMRP expression enhances efferocytosis and cholesterol transport in vitro and in vivo.

• IRE1 kinase inhibition by a small molecule inhibitor or genetic deletion of FMRP in macrophages alleviates atherosclerotic plaque formation.

Please upload your synopsis image as an independent tiff, jpeg or PNG file 550 px wide x 300-600 px high. The text should remain legible.

5/ As part of the EMBO Publications transparent editorial process initiative (see our Editorial at http://embomolmed.embopress.org/content/2/9/329), EMBO Molecular Medicine will publish online a Review Process File (RPF)

to accompany accepted manuscripts.
This file will be published in conjunction with your paper and will include the anonymous referee reports, your point-by-point response and all pertinent correspondence relating to the manuscript. Let us know whether you agree with the publication of the RPF and as here, if you want to remove or not any figures from it prior to publication.
Please note that the Authors checklist will be published at the end of the RPF.

I look forward to receiving your revised manuscript.

With kind regards,

Lise Roth

Lise Roth, PhD
Editor
EMBO Molecular Medicine

*Additional important information regarding Figures

Each figure should be given in a separate file and should have the following resolution:
Graphs 800-1,200 DPI
Photos 400-800 DPI
Colour (only CMYK) 300-400 DPI"

Figures are not edited by the production team. All lettering should be the same size and style; figure panels should be indicated by capital letters (A, B, C etc). Gridlines are not allowed except for log plots. Figures should be numbered in the order of their appearance in the text with Arabic numerals. Each Figure must have a separate legend and a caption is needed for each panel.

*Additional important information regarding figures and illustrations can be found at https://bit.ly/EMBOPressFigurePreparationGuideline. See also figure legend preparation guidelines: https://www.embopress.org/page/journal/17574684/authorguide#figureformat

The system will prompt you to fill in your funding and payment information. This will allow Wiley to send you a quote for the article processing charge (APC) in case of acceptance. This quote takes into account any reduction or fee waivers that you may be eligible for. Authors do not need to pay any fees before their manuscript is accepted and transferred to our publisher.

***** Reviewer's comments *****

Referee #2 (Remarks for Author):

Yildirim et al. have made substantial improvements to the manuscript and addressed all concerns.

Referee #3 (Comments on Novelty/Model System for Author):

The revised manuscript has appropriately addressed all of my prior concerns.

Referee #3 (Remarks for Author):

In the revision, the authors have appropriately addressed prior concerns.

The authors performed the requested changes.

Minor suggestions:

Question 1: On Page 4, line 58 "Endogenous FMRP and IRE1, migrated faster than EGFP-FMRP and FLAG-IRE1, respectively." Clarify - migrated faster upon SDS-PAGE?

Response 1: We revised the description of the experiment as *"Endogenous FMRP and IRE1, migrated faster on SDS-PAGE than epitope tagged EGFP-FMRP and FLAG-IRE1, respectively"*.

Question 2: Page 5, line 207 "Next, we fed Apoe-/- mice with a WD (12 weeks) and injected them daily with the IRE1 kinase inhibitor" Not sure why ? is present

Response 2: We thank the reviewer for pointing out this typing mistake. We deleted the question mark (?) in our revised manuscript.

4th Feb 2022

Dear Dr. Erbay,

Thank you for submitting your revised files. I am pleased to inform you that your manuscript is accepted for publication and is now being sent to our publisher to be included in the next available issue of EMBO Molecular Medicine.

Thank you for depositing the spectrometry proteomics data, please note that these datasets must be made public before online publication of the manuscript.

We would also like to remind you that as part of the EMBO Publications transparent editorial process initiative, EMBO Molecular Medicine will publish a Review Process File online to accompany accepted manuscripts. If you do NOT want the file to be published or would like to exclude figures, please immediately inform the editorial office via e-mail.

Please read below for additional IMPORTANT information regarding your article, its publication and the production process.

Congratulations on your interesting work!

With kind regards,

Lise Roth

Lise Roth, Ph.D
Scientific Editor
EMBO Molecular Medicine

EMBO PRESS

YOU MUST COMPLETE ALL CELLS WITH A PINK BACKGROUND ➔

PLEASE NOTE THAT THIS CHECKLIST WILL BE PUBLISHED ALONGSIDE YOUR PAPER

Corresponding Author Name: Ebru Erbay

Journal Submitted to: EMBO Molecular Medicine

Manuscript Number: EMM-2021-15344

Reporting Checklist For Life Sciences Articles (Rev. June 2017)

This checklist is used to ensure good reporting standards and to improve the reproducibility of published results. These guidelines are consistent with the Principles and Guidelines for Reporting Preclinical Research issued by the NIH in 2014. Please follow the journal's authorship guidelines in preparing your manuscript.

A- Figures

1. Data

The data shown in figures should satisfy the following conditions:

- ➔ the data were obtained and processed according to the field's best practice and are presented to reflect the results of the experiments in an accurate and unbiased manner.
- ➔ figure panels include only data points, measurements or observations that can be compared to each other in a scientifically meaningful way.
- ➔ graphs include clearly labeled error bars for independent experiments and sample sizes. Unless justified, error bars should not be shown for technical replicates.
- ➔ if n< 5, the individual data points from each experiment should be plotted and any statistical test employed should be justified
- ➔ Source Data should be included to report the data underlying graphs. Please follow the guidelines set out in the author ship guidelines on Data Presentation.

2. Captions

Each figure caption should contain the following information, for each panel where they are relevant:

- ➔ a specification of the experimental system investigated (eg cell line, species name).
- ➔ the assay(s) and method(s) used to carry out the reported observations and measurements
- ➔ an explicit mention of the biological and chemical entity(ies) that are being measured.
- ➔ an explicit mention of the biological and chemical entity(ies) that are altered/varied/perturbed in a controlled manner.

- ➔ the exact sample size (n) for each experimental group/condition, given as a number, not a range;
- ➔ a description of the sample collection allowing the reader to understand whether the samples represent technical or biological replicates (including how many animals, litters, cultures, etc.).
- ➔ a statement of how many times the experiment shown was independently replicated in the laboratory.
- ➔ definitions of statistical methods and measures:
  - common tests, such as t-test (please specify whether paired vs. unpaired), simple χ2 tests, Wilcoxon and Mann-Whitney tests, can be unambiguously identified by name only, but more complex techniques should be described in the methods section;
  - are tests one-sided or two-sided?
  - are there adjustments for multiple comparisons?
  - exact statistical test results, e.g., P values = x but not P values < x;
  - definition of 'center values' as median or average;
  - definition of error bars as s.d. or s.e.m.

Any descriptions too long for the figure legend should be included in the methods section and/or with the source data.

In the pink boxes below, please ensure that the answers to the following questions are reported in the manuscript itself. Every question should be answered. If the question is not relevant to your research, please write NA (non applicable). We encourage you to include a specific subsection in the methods section for statistics, reagents, animal models and human subjects.

USEFUL LINKS FOR COMPLETING THIS FORM

http://www.antibodypedia.com
http://1degreebio.org
http://www.equator-network.org/reporting-guidelines/improving-bioscience-research-repor

http://grants.nih.gov/grants/olaw/olaw.htm
http://www.mrc.ac.uk/Ourresearch/Ethicsresearchguidance/Useofanimals/index.htm
http://ClinicalTrials.gov
http://www.consort-statement.org
http://www.consort-statement.org/checklists/view/32-consort/66-title
http://www.equator-network.org/reporting-guidelines/reporting-recommendations-for-tumo
http://datadryad.org
http://figshare.com
http://www.ncbi.nlm.nih.gov/gap
http://www.ebi.ac.uk/ega
http://biomodels.net/
http://biomodels.net/miriam/
http://jjj.biochem.sun.ac.za
https://osp.od.nih.gov/biosafety-biosecurity-and-emerging-biotechnology/
http://www.selectagents.gov/

B- Statistics and general methods

Please fill out these boxes ➔ (Do not worry if you cannot see all your text once you press return)

1.a. How was the sample size chosen to ensure adequate power to detect a pre-specified effect size?	A pilot experiment or prior published studies from our group were used to perform power analysis to calculate the effectiveness size.
1.b. For animal studies, include a statement about sample size estimate even if no statistical methods were used.	Based on preliminary experiments, assuming a 50% alteration in atherogenic response with a pooled S.D. of 35%, we will have 90% power to detect diferences. (alpha=0.05 two-tailed) using a sample size of 7 animals.
2. Describe inclusion/exclusion criteria if samples or animals were excluded from the analysis. Were the criteria pre-established?	The only elimination criteria used for mouse studies was based on health and as advised by a veterinarian.
3. Were any steps taken to minimize the effects of subjective bias when allocating animals/samples to treatment (e.g. randomization procedure)? If yes, please describe.	Researchers were blinded to the identity of treatment and control groups.
For animal studies, include a statement about randomization even if no randomization was used.	Mice were randomly assigned to independent cohorts.
4.a. Were any steps taken to minimize the effects of subjective bias during group allocation or/and when assessing results (e.g. blinding of the investigator)? If yes please describe.	Quantification of results from in vivo experiments were conducted by researchers that were blinded to the identity of treatment and control groups.
4.b. For animal studies, include a statement about blinding even if no blinding was done	In vivo experiments were conducted by researchers that were blinded to the identity of treatment and control groups.
5. For every figure, are statistical tests justified as appropriate?	Yes.
Do the data meet the assumptions of the tests (e.g., normal distribution)? Describe any methods used to assess it.	For in vitro experiments with smaller sample size the Student's t-test was used with Welch correction. For in vivo experiments Student's t-test with Nonparametric Mann-Whitney test was used.
Is there an estimate of variation within each group of data?	Yes.
Is the variance similar between the groups that are being statistically compared?	No, Welch correction was applied to correct for differences in standard deviation between the groups.

C- Reagents

6. To show that antibodies were profiled for use in the system under study (assay and species), provide a citation, catalog number and/or clone number, supplementary information or reference to an antibody validation profile. e.g., Antibodypedia (see link list at top right), 1DegreeBio (see link list at top right).	anti-pIRE1 (phsopho-S724; 124945), anti-FMRP (ab17722), anti-LRP1 (ab92544), anti-MMP9 (ab38898), anti-α-SMA (ab5694) and anti-Thiophosphate ester antibody (ab133473) antibodies purchased from Abcam; anti-pFMRP (phosphor-S499; p1125-499) from PhosphoSolutions; anti-IRE1 (3294), anti-LRP1 (64099) and anti-FMRP (4317) from Cell-Signaling; anti-ABCA1 (NB400-105) and anti-ABCG1 (NB400-132) from Novus Biologicals; anti-β-Actin-horse radish peroxidase (47778) from Santa Cruz Biotechnology, anti-MOMA-2 from Bio-Rad; anti-FMRP (834601), anti-PE-F4/80 (123110), TruStain FcX (anti-CD16/32, 101319), anti-CD45-Pac. Blue (clone 30-F11), CD3e-PE (Clone 145-2C11), CD11b-APC (Clone M1/70), CD19-BV650 (Clone 6D5), Ly6C-PE/Dazzle (Clone HK1.4) and Ly6G-PerCP Cy5.5 (1A8) from Biolegend. All the antibodies are from commercial sources and have been validated according to data are available on the manufacturer's website or in prior studies.
7. Identify the source of cell lines and report if they were recently authenticated (e.g., by STR profiling) and tested for mycoplasma contamination.	Fmr1-/- mouse embryonic fibroblasts (MEF) were generated in Dr. David Nelson's laboratory (Baylor College of Medicine, Houston, Texas). Human embryonic kidney 293T (HEK293T), Jurkat (human T lymphocytes) and L-929 (mouse fibroblasts) cells were obtained from ATCC.

* for all hyperlinks, please see the table at the top right of the document

D- Animal Models

8. Report species, strain, gender, age of animals and genetic modification status where applicable. Please detail housing and husbandry conditions and the source of animals.	C57BL/6 (WT, Fmr1+/+), Fmr1-/- and Apolipoprotein E-deficient (Apoe-/-) mice were purchased from Jackson Laboratory. IRE1 conditional knock out (Ire1αflox/flox) mice were a kind gift from Dr. Takao Iwawaki (Kanazawa Medical University, Japan) and characterized before (80). Ire1αflox/flox mice were inter-crossed from with LysMcre mice purchased from Jackson Lab (004781) to obtain Ire1αflox/flox, LysMcre mice (IRE1-/-), which had a myeloid-specific Ire1α gene deletion. Fmr1 conditional knock out (Fmr1flox/flox) mice were a kind gift from dr. David Nelson. Fmr1flox/flox mice were inter-crossed with LysMcre mice purchased from Jackson Lab (004781) to obtain myeloid Fmr1-deficient (myFmr1-/-) mice.
9. For experiments involving live vertebrates, include a statement of compliance with ethical regulations and identify the committee(s) approving the experiments.	All animal experiments were performed according to protocols approved by the Experimental Animal Ethical Care Committees at Bilkent University, Ankara, Turkey or Cedars Sinai Medical Center, Los Angeles, USA or the University of Ottawa Animal Care Committee, Ottawa, ON K1N 6N5, Canada.
10. We recommend consulting the ARRIVE guidelines (see link list at top right) (PLoS Biol. 8(6), e1000412, 2010) to ensure that other relevant aspects of animal studies are adequately reported. See author guidelines, under 'Reporting Guidelines'. See also: NIH (see link list at top right) and MRC (see link list at top right) recommendations. Please confirm compliance.	We confirm compliance with the ARRIVE guidelines.

E- Human Subjects

11. Identify the committee(s) approving the study protocol.	NA
12. Include a statement confirming that informed consent was obtained from all subjects and that the experiments conformed to the principles set out in the WMA Declaration of Helsinki and the Department of Health and Human Services Belmont Report.	NA
13. For publication of patient photos, include a statement confirming that consent to publish was obtained.	NA
14. Report any restrictions on the availability (and/or on the use) of human data or samples.	NA
15. Report the clinical trial registration number (at ClinicalTrials.gov or equivalent), where applicable.	NA
16. For phase II and III randomized controlled trials, please refer to the CONSORT flow diagram (see link list at top right) and submit the CONSORT checklist (see link list at top right) with your submission. See author guidelines, under 'Reporting Guidelines'. Please confirm you have submitted this list.	NA
17. For tumor marker prognostic studies, we recommend that you follow the REMARK reporting guidelines (see link list at top right). See author guidelines, under 'Reporting Guidelines'. Please confirm you have followed these guidelines.	NA

F- Data Accessibility

18: Provide a "Data Availability" section at the end of the Materials & Methods, listing the accession codes for data generated in this study and deposited in a public database (e.g. RNA-Seq data: Gene Expression Omnibus GSE39462, Proteomics data: PRIDE PXD000208 etc.) Please refer to our author guidelines for 'Data Deposition'.<br><br>Data deposition in a public repository is mandatory for:<br>a. Protein, DNA and RNA sequences<br>b. Macromolecular structures<br>c. Crystallographic data for small molecules<br>d. Functional genomics data<br>e. Proteomics and molecular interactions	All data are available in the main text or the supplementary materials. Research materials used in the article can be requested from authors. The mass spectrometry proteomics data have been deposited to the ProteomeXchange Consortium via the PRIDE partner repository with the dataset identifier PXD030594 and 10.6019/PXD030594.
19. Deposition is strongly recommended for any datasets that are central and integral to the study; please consider the journal's data policy. If no structured public repository exists for a given data type, we encourage the provision of datasets in the manuscript as a Supplementary Document (see author guidelines under 'Expanded View' or in unstructured repositories such as Dryad (see link list at top right) or Figshare (see link list at top right).	NA
20. Access to human clinical and genomic datasets should be provided with as few restrictions as possible while respecting ethical obligations to the patients and relevant medical and legal issues. If practically possible and compatible with the individual consent agreement used in the study, such data should be deposited in one of the major public access-controlled repositories such as dbGAP (see link list at top right) or EGA (see link list at top right).	NA
21. Computational models that are central and integral to a study should be shared without restrictions and provided in a machine-readable form. The relevant accession numbers or links should be provided. When possible, standardized format (SBML, CellML) should be used instead of scripts (e.g. MATLAB). Authors are strongly encouraged to follow the MIRIAM guidelines (see link list at top right) and deposit their model in a public database such as Biomodels (see link list at top right) or JWS Online (see link list at top right). If computer source code is provided with the paper, it should be deposited in a public repository or included in supplementary information.	NA

G- Dual use research of concern

22. Could your study fall under dual use research restrictions? Please check biosecurity documents (see link list at top right) and list of select agents and toxins (APHIS/CDC) (see link list at top right). According to our biosecurity guidelines, provide a statement only if it could.	NA
