# Supplementary material for: Intercepting IRE1 kinase‐FMRP signaling prevents atherosclerosis progression
Source: EMBO Mol Med. 2022 Feb 22;14(4):e15344. doi: 10.15252/emmm.202115344 (PMC8988208; doi:10.15252/emmm.202115344)

Appendix Figure S6 Source Data

Appendix Fig. S6A

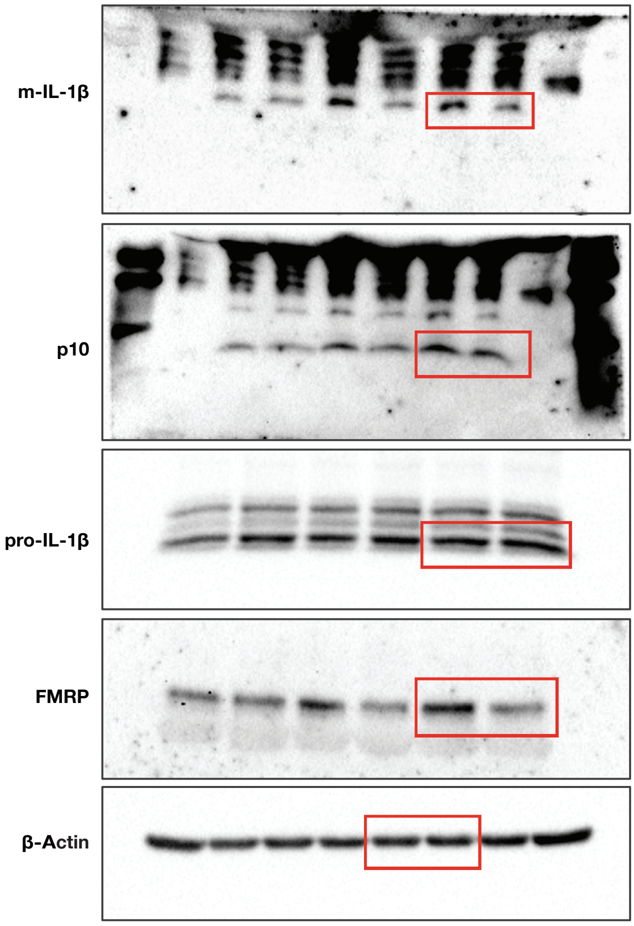

Appendix Fig. S6B

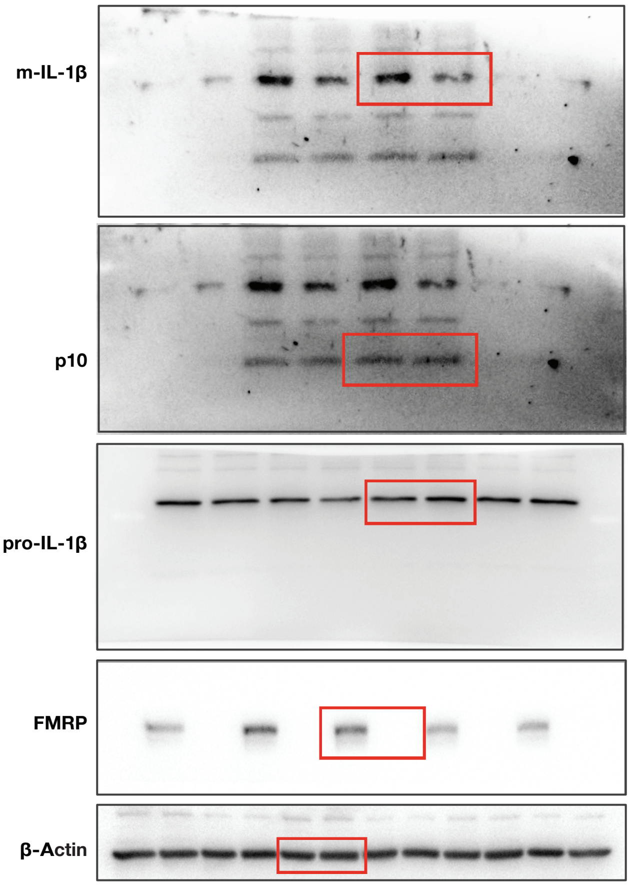

Appendix Fig. S6C

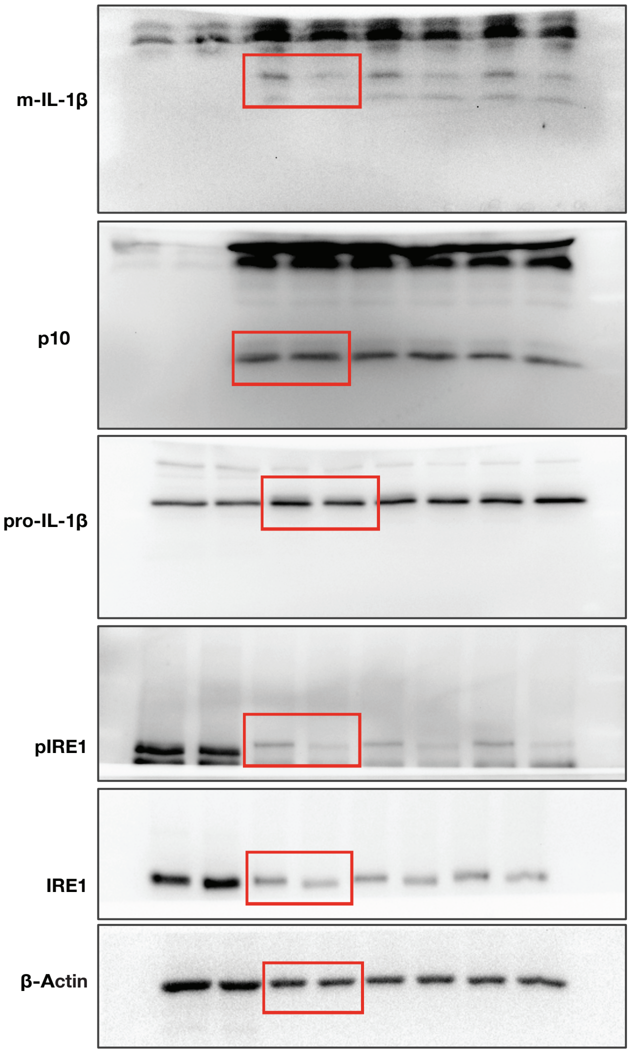

Supplement: Supplementary file 2 — Source Data for Appendix [file EMMM-14-e15344-s004.zip › EMM-2021-15344_SourceDataForAppendixFigureS6.zip!/EMM-2021-15344_SourceDataForAppendixFigureS6.pdf]
